# Variability of Gene Expression Identifies Transcriptional Regulators of Early Human Embryonic Development

**DOI:** 10.1371/journal.pgen.1005428

**Published:** 2015-08-19

**Authors:** Yu Hasegawa, Deanne Taylor, Dmitry A. Ovchinnikov, Ernst J. Wolvetang, Laurence de Torrenté, Jessica C. Mar

**Affiliations:** 1 Department of Systems and Computational Biology, Albert Einstein College of Medicine, Bronx, New York, United States of America; 2 Division of Life Science, Graduate School of Life Science, Hokkaido University, Sapporo, Hokkaido, Japan; 3 RMANJ Reproductive Medicine Associates of New Jersey, Morristown, New Jersey, United States of America; 4 Division of Reproductive Endocrinology, Department of Obstetrics, Gynecology, and Reproductive Science, Robert Wood Johnson Medical School, Rutgers University, New Brunswick, New Jersey, United States of America; 5 Australian Institute for Bioengineering and Nanotechnology, University of Queensland, Brisbane, Queensland, Australia; 6 Department of Epidemiology and Population Health, Albert Einstein College of Medicine, Bronx, New York, United States of America; Georgia Institute of Technology, UNITED STATES

## Abstract

An analysis of gene expression variability can provide an insightful window into how regulatory control is distributed across the transcriptome. In a single cell analysis, the inter-cellular variability of gene expression measures the consistency of transcript copy numbers observed between cells in the same population. Application of these ideas to the study of early human embryonic development may reveal important insights into the transcriptional programs controlling this process, based on which components are most tightly regulated. Using a published single cell RNA-seq data set of human embryos collected at four-cell, eight-cell, morula and blastocyst stages, we identified genes with the most stable, invariant expression across all four developmental stages. Stably-expressed genes were found to be enriched for those sharing indispensable features, including essentiality, haploinsufficiency, and ubiquitous expression. The stable genes were less likely to be associated with loss-of-function variant genes or human recessive disease genes affected by a DNA copy number variant deletion, suggesting that stable genes have a functional impact on the regulation of some of the basic cellular processes. Genes with low expression variability at early stages of development are involved in regulation of DNA methylation, responses to hypoxia and telomerase activity, whereas by the blastocyst stage, low-variability genes are enriched for metabolic processes as well as telomerase signaling. Based on changes in expression variability, we identified a putative set of gene expression markers of morulae and blastocyst stages. Experimental validation of a blastocyst-expressed variability marker demonstrated that *HDDC2* plays a role in the maintenance of pluripotency in human ES and iPS cells. Collectively our analyses identified new regulators involved in human embryonic development that would have otherwise been missed using methods that focus on assessment of the average expression levels; in doing so, we highlight the value of studying expression variability for single cell RNA-seq data.

## Author Summary

In order to function properly, cells express specific sets of genes that are regulated via complex transcriptional programs. During early stages of development, when an embryo consists of only a handful of cells, it is vital that these cells work together so that the embryo can develop into a healthy baby. Single cell studies allow us to understand how each cell contributes to ensuring proper regulation of the embryo, as well as identify the critical genes whose expression is important for development. While we understand that regulation of a gene occurs through the timing of when it is expressed and also the quantity of its expression, more recently we have come to recognize that the variability of a gene’s expression across single cells may also contribute to the viability of the organism. In this study, we analyzed the gene expression variability of human embryos at different developmental stages. We discovered distinctive patterns of variability across cells in the embryo; some genes had extremely stable expression, and others were variable but with increased homogeneity in expression at a particular developmental stage. We validated one of these stage-specific markers and found that it played a role in the maintenance of pluripotency of human pluripotent stem cells. Overall, these results can help unlock additional clues into understanding how embryonic development is regulated in humans.

## Introduction

The regulatory program that ensures that a human embryo can develop successfully starting from a single cell zygote is one of the most fascinating examples of systems-level genetic control. During development, individual cells must quickly respond to internal and external signals while the number of cells that make up an embryo increases at a rapid rate. How an embryo is able to coordinate signals cooperatively across all cells, while subsets of cells undergo diverse fate transitions to specific lineages remains an open question. Inherent in an embryo’s regulatory program is the need to balance flexibility with robustness to ensure that development can continue in spite of perturbations that may occur. Studying how individual cells alter their transcriptomes as an embryo transitions through each developmental stage presents an opportunity to understand the core of the regulatory program, and specifically how robustness is maintained throughout development.

Single cell technology has revolutionized almost every arena of the biological sciences but perhaps none more significantly than developmental biology. Profiling the transcriptomes of individual cells provides a means to disentangle heterogeneous properties that can identify small numbers of distinct or rare cells amongst a population of cells that otherwise appear identical based on a handful of markers [1]. As early as the 2000s, studies have demonstrated the limitations of inferences derived from bulk cell approaches, where transcriptomes from multiple cells are combined to create an ensemble representation of a generalized single cell [2–4]. This ensemble-based model, referred to as an “averaged cell” by Levsky and Singer [5] is unable to capture the variability inherent in gene expression in cell populations and therefore provides only a marginal insight into transcriptional regulation. Applying single cell profiling to understand developmental processes has been invaluable for pinpointing specific genes that direct cell fate transitions towards distinct lineages [6]. The ability to resolve heterogeneity in gene expression amongst cell populations has been useful in revising and identifying more specific cell types; and in the process, we are only beginning to appreciate just how diverse the transcriptional states underlying each developmental stage can be [7].

**Fig 1:**
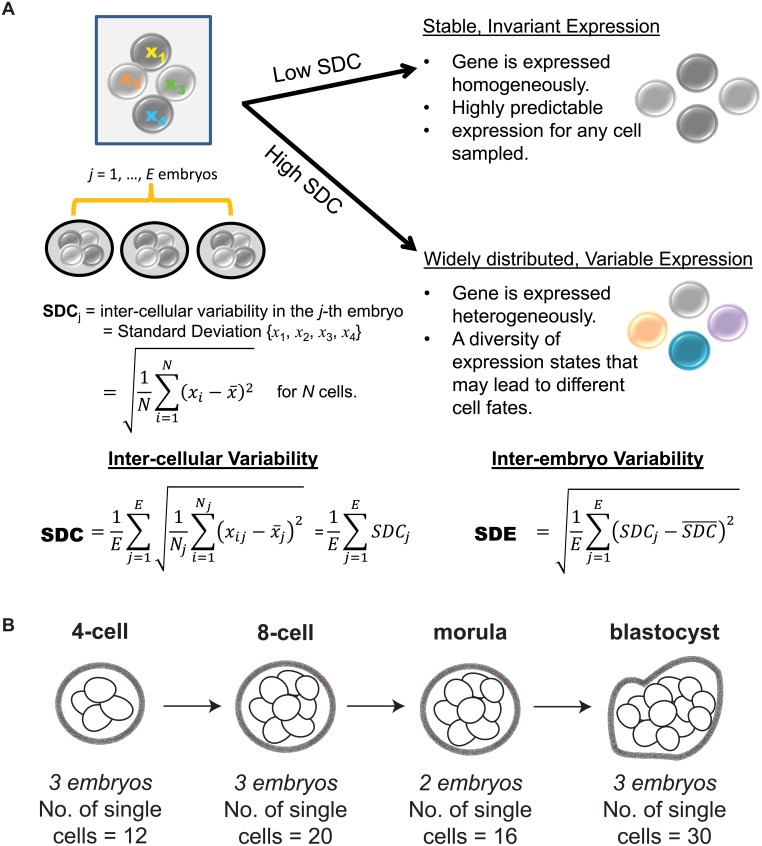
Investigating inter-cellular gene expression variability. **(A)** Interpreting measures of inter-cellular expression variability in a cell population and implications for understanding regulatory control. **(B)** Experimental design of the Yan data set.

Studying transcription at the single cell level has forced us to confront the fact that gene expression consists of both signal and noise [8–10]. While the recognition that transcription is a stochastic process predates single cell technology [11, 12], it is only recently that we have come to appreciate the insight that variability in gene expression can shed on understanding regulatory control [13–15]. In the context of single cells, inter-cellular variability represents a measure of consistency or dispersion of a specific signal amongst a cell population. As a byproduct, expression variability also inversely reflects our ability to predict or have confidence in the transcriptional state for a new cell. For instance, a gene with low expression variability has high generalizability for any future cells sampled at random and therefore may be valuable as a marker of that cell’s state. Conversely, a gene with high expression variability is one whose expression levels fluctuate widely across cells in the population, and this heterogeneity could be due to several factors that can provide information about its regulation, e.g. a difference in cell cycling, cell fate, or stochastic component of the regulatory program used by the cell [16–18] (see Fig 1A).

When characterizing a phenotype based on differential gene expression, the statistical methods that are typically used, such as a t-test or ANOVA, are based on deviations detected in average expression and assume constant levels of variability. While this is a sensible approach for identifying overall shifts, we acknowledge that studying dispersion or variability in expression is also important to understanding regulation in single cells [19, 20]. Single molecule studies performed at single cell resolution have demonstrated that gene expression is inherently stochastic, where even amongst isogenic cells, a gene’s expression level is not identically distributed [21]. Most of this stochasticity is because the production of a transcript requires the coordination of multiple components in a cell, some of which are present at very low concentrations. Gene expression can be modeled as a probabilistic event, and thus in a cell population, each cell contains a gene that is expressed in an all-or-nothing, binary manner according to a certain probability. This generates cell-to-cell heterogeneity in the population where some cells have genes whose expression is switched on while in other cells, expression is off [22]. Consequently, variability represents a very real component to understand gene expression, and by assuming constant variability and not studying this property directly, we may miss out on identifying key regulators in single cells [23, 24].

**Fig 2:**
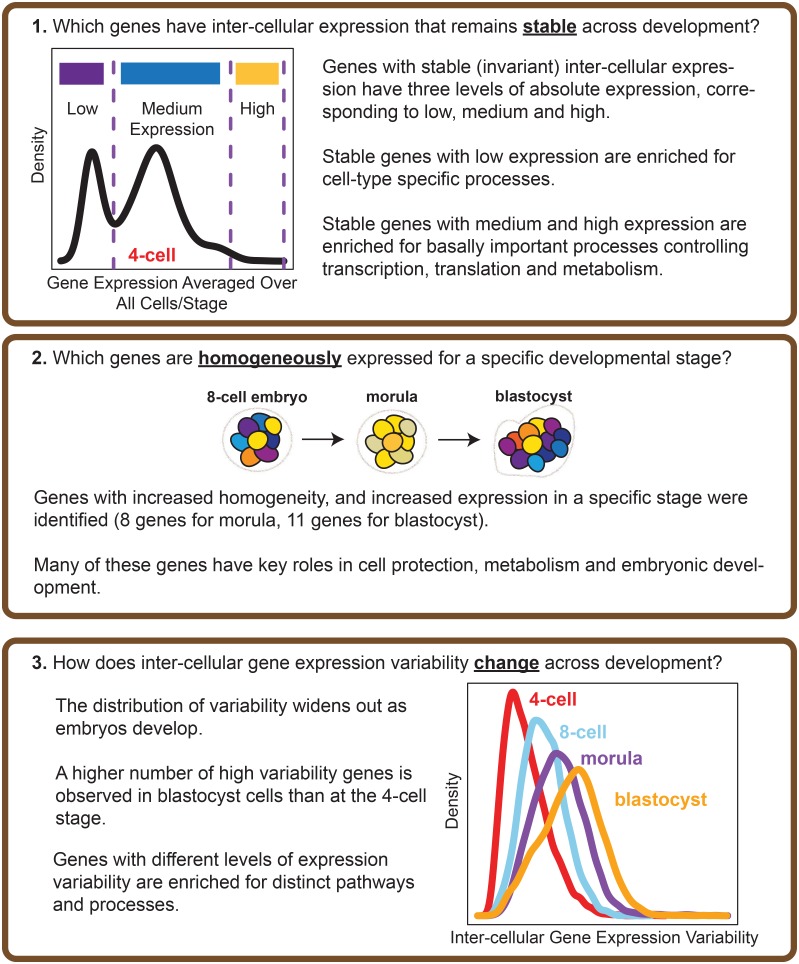
Overview of the analysis performed. The questions that our study seeks to address, and the main results obtained are highlighted.

Recently, Yan *et al*. [25] used single cell RNA-sequencing (RNA-seq) to elucidate the transcriptional landscape of preimplantation human embryos from distinct stages of development (Fig 1B). This study used RNA-seq to comprehensively profile individual cells that were derived from the same human embryo at early stages of development. Major classes of genes with key temporal changes over the course of development were identified, including novel lncRNAs regulators, potentially new protein-coding transcripts, as well as dynamic patterns of alternative splicing. The application of single cell profiling provided new insights into transcriptional regulation of human embryos. It is worth highlighting, however, that the analytical methods used by Yan *et al*. [25] are based on averaging expression from the cell populations. While this is adequate for identifying average trends, their study fails to use the valuable opportunity presented in this data set to understand how variability in gene expression is distributed at the cell-to-cell level. For single cell RNA-seq data, an analysis of expression variability is useful in identifying genes that are invariantly or heterogeneously expressed in cell populations, which in turn may provide insight into regulation. Here, we use the data set generated by Yan *et al*. [25] to demonstrate the additional utility that analyzing variability of gene expression can bring to our understanding of transcriptional control in human embryonic development (see Fig 2 for an overview of the analysis performed). Our analysis approach is composed of the following key steps: (1) identification of stable genes across development, (2) detection of stage-specific variability markers (3) identification of control states based on different levels of expression variability.

Our results show that certain genes do have extremely stable inter-cellular expression during development, and that this stability appears to play an important role in regulating the cell. Stable genes are expressed across a range of levels, and those with low expression are enriched for cell type-specific processes whereas those with high expression are involved in fundamentally important cellular processes like maintenance, and metabolism. We also identified genes that could function as markers of stage, as these genes are noticeably expressed with increased homogeneity for cells of a particular developmental stage. Overall, our findings point to variability in gene expression as a regulatory feature of developmental processes in human embryos.

## Results

### The single cell RNA-seq data set on human embryos was inspected to ensure appropriate quality control of the data

The embryos that were profiled in Yan *et al*. [25] were donated by women who had already undergone successful *in vitro* fertilization (IVF), delivered a healthy baby, and elected to donate the remaining high-quality embryos that had been cryopreserved. On average, the women were 30 years old, all had tubal-factor infertility, and all had partners with normal semen parameters. To be included in the profiling study, the embryos underwent extensive screening for good morphology and high quality. Quality assessments were performed using externally-derived, established scoring criteria for viability of the embryos [26], in conjunction with explicit definitions of embryonic stage (as outlined in the Materials and Methods section of Yan *et al*. [25]). To the best of our knowledge, we are confident that these embryos were viable.

**Fig 3:**
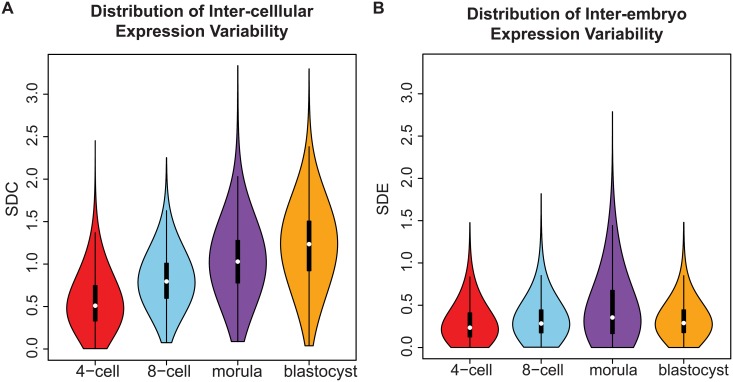
Distribution of gene expression variability during embryonic development. **(A)** The distribution reflects the inter-cellular expression variability for all genes in the transcriptome. An overall widening of the distribution is observed as embryos transition from the 4-cell to the blastocyst stage. As a result, there is an increase in the number of genes with higher levels of expression variability in the blastocyst stage than the 4-cell stage. **(B)** How the inter-cellular expression variability varies amongst the embryos is captured by the SDE. From the density distribution, we see that the embryos have stable profiles during all four stages of development.

We tested the data to ensure that an appropriate standard of data quality was met for studying gene expression variability. Stringent thresholds were used to discard genes that were expressed below a specific quality threshold in the majority of cells that were profiled (Materials and Methods, S1 Text). To measure inter-cellular expression variability, we adopted a statistic termed the SDC that represents the standard deviation (SD) of gene expression between cells of the same embryo that is averaged across multiple embryos (Materials and Methods, Fig 1A). The data were inspected to ensure that patterns of inter-cellular variability were not influenced by the number of cells contributing to each stage (see S1 Text). We also investigated the consistency of the inter-cellular variability measures between the embryos and observed that the transcriptomes of the embryos were relatively stable compared to each other (see Fig 3B). This observation lends support to treating the embryos as replicates to estimate the inter-cellular expression variability in our study (see S1 Text). We also evaluated the possibility that chromosomal copy number mosaicism could affect the variability analysis for those genes expressed on an aneuploid chromosome in a subset of cells. To check for chromosomal copy number effects on expression, we compared the normalized gene expression distribution for every chromosome (see S2 Text) and found no obvious aberrations in chromosome-wide expression levels [27].

### Using standard deviation as a statistic to study expression variability

The analysis of gene expression variability remains an area where guidelines for the best statistical practices are still maturing. For this reason, we investigated the utility of using two statistics, the SDC, based on the SD and the coefficient of variation (CV) for studying gene expression variability. The CV is a standardized measure of variability or dispersion that is calculated by taking the ratio of the SD and the average. It is a measure that has previously been used in studies of expression variability for microarray-based data [13, 14]. One advantage of the CV is that it addresses any potential correlation between the variability and average. Nevertheless, it can be more difficult to interpret what a CV value represents for an individual gene with respect to its average expression and expression variability. Another potential issue is that as a ratio of two statistics, the CV is also in theory subject to zero-inflation for genes that have very low levels of average expression. Such genes will be falsely assigned a higher level of variability as measured by the CV, independent of how dispersed the data for this gene really is.

A factor that often arises when studying expression variability is the assumption that the mean and variability of a gene are correlated. Based on our comparison of the CV and SD, the two statistics appear to handle the nature of this correlation quite differently (see S3 Text). We found that the CV had a higher negative correlation with the average expression than the SDC for all four stages. We also conducted simulation studies to test the ability of the two statistics to identify genes with three different levels of variability. We tested the SDC and CV on three different test cases and overall, the SDC appeared to be the better performing statistic with the ability to identify the correct number of variability states more decisively than the CV, and also in general, increased precision in classifying genes to their correct level of variability. We also repeated our analyses of expression variability to compare the results of our main findings when the CV statistic was used. We found that although there was an overlap in the stable genes identified, the two statistics still measure different properties of expression variability. We also noticed that within a specific developmental stage, genes with a low SDC and high average expression, were more likely to have a low CV value, however the converse was not true. Genes with a low CV had both average values and SDC values that spanned a wide spectrum (in some cases greater than the third quartile for SDC, and less than the first quartile for average expression). Therefore, for applications such as marker detection, using CV may be limited as it does not provide the kind of control that comes from setting specific filters on the SDC and average expression directly. Overall, our analyses, which are outlined in more detail in S3 Text, point to the SDC as being the more informative statistic to study gene expression variability for the Yan data set.

### Genes with stable expression are involved in critical processes related to cell survival, metabolism, specialized cell types, and can be classified into three distinct modes of absolute expression

**Fig 4:**
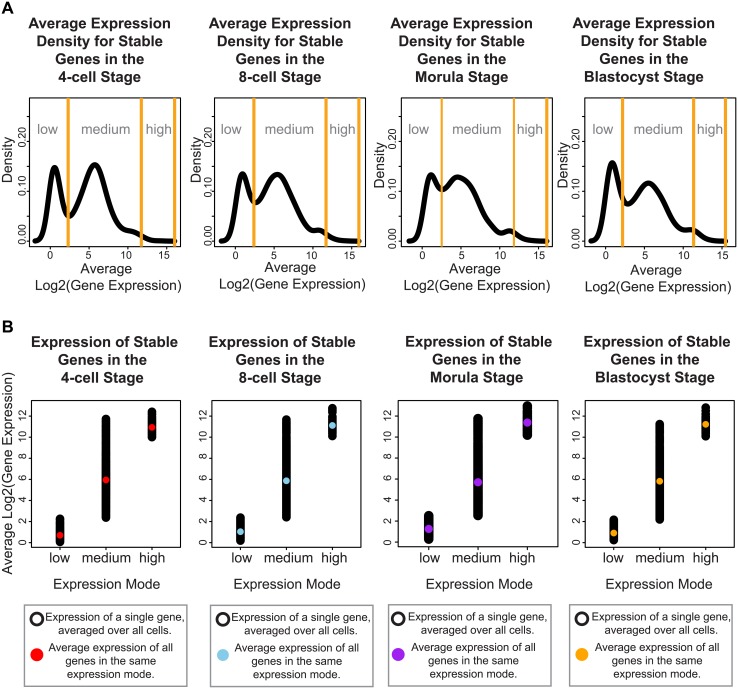
Stable genes can be classified into three distinct modes of expression activity. For each developmental stage, inspection of the absolute expression of the stable genes we identified, reveals that these genes fall into three expression modes spanning low, medium and high levels of expression. **(A)** The black lines denote the smoothed density of gene expression levels that have been averaged across all cells from a specific developmental stage. The yellow lines indicate the boundaries defining the three expression modes, these boundaries were calculated using a mixture model that clustered the genes into these three groups. **(B)** Single cell gene expression profiles for the stable genes for each developmental stage. These profiles have been plotted based on which expression mode they have been clustered into using the mixture model. The black dots represent the expression level of the stable genes in a single cell. The colored dot represents the average expression per expression mode.

Focusing on genes whose expression remains invariant amongst all cells across the developmental stages may reveal the subset of core regulators that are integrally important to the developing embryo. Genes expressed at very precise levels in a cell population may correspond to widespread regulators that are ubiquitously switched on to maintain homeostasis, or reflect specialized processes that must be systematically turned off until the embryo is ready. We used Levene’s test to determine those genes with a non-significant change in expression across development (adjusted P-value > 0.05), and clustered the SDC values of these genes using a Normal mixture model to identify the gene clusters with low, medium and high variability. We identified 955 genes whose variability in gene expression varied the least across the four stages (see Materials and Methods, S1 Table, S1 Fig) and this group was designated as the group of stable genes. Inspection of the stable genes revealed that they could be classified into three distinct modes of absolute expression; corresponding to low, medium, and high levels of expression (Fig 4).

To understand how these different modes may be contributing to developmental regulation, we identified pathways and functions through Ingenuity Pathway Analysis (IPA) that were significantly enriched in each of these three groups. Overall, we observed that stable genes with low levels of expression were involved in pathways regulating specialized cell types (see S2 Table). For all four stages, we saw terms that related to disease processes affecting a specific cell type, e.g. melanoma and chronic leukemia. For specific stages, we also observed enrichment of tissue-specific diseases that affect different organs, such as the kidney (renal cancer, 4-cell and 8-cell and blastocyst), endometrium (endometrium carcinoma, 4-cell, and blastocyst), head and neck (8-cell), and colon (blastocyst) (see S2 Table).

For the stable genes with medium levels of expression, 59% were found to be common to all four stages, suggesting that they form a core set of housekeeping-type genes that are expressed in a non-stage specific nature (S3 Table, see S2 Fig for single cell profiles of some of the stable genes with medium expression). This gene set was enriched for fundamentally important pathways, including those controlling transcription (cleavage and polyadenylation of pre-mRNA), translation (EIF2 signaling, regulation of eIF4 and p70S6K signaling), protein ubiquitination, mTOR signaling and DNA damage (cell cycle: G2/M damage checkpoint regulation) (see S3A Table). The medium-expressed group of stable genes were also enriched for signaling pathways involving key growth factors and receptors (e.g. ERK5 signaling, estrogen signaling, and ephrin signaling). We observed enrichment for processes related to structural remodeling (e.g. remodeling of epithelial adherens junctions, epithelial adherens junction signaling, and regulation of actin-based motility by Rho). These are important processes for the growing embryo, as critical inter-cellular communication is known to be transmitted via adherens junctions. Enrichment in functional terms obtained from IPA support these themes as well, where enriched key terms include “initiation of translation of mRNA”, “protein and expression of RNA” (see S3B Table). Significant terms related to infection were also observed (“infection of embryonic cell lines”, “epithelial cell lines”, “viral infection”, “HIV infection”, “infection by HIV-1”, “infection by RNA virus”), and these terms are likely to reflect the massive degree of cellular proliferation occurring in the embryo.

The stable genes with high levels of average expression were among the smallest subset identified. These genes overlapped significantly with pathways already detected in the stable gene set with medium levels of average expression. Specifically, these genes were enriched for EIF2 signaling, regulation of eIF4 and p70S6K signaling, and mTOR signaling at all stages (S4 Table). For the blastocyst stage, significant enrichment of processes involving cell cycle (G1/S checkpoint regulation) and NADH repair was observed.

### Distinguishing contributions from the maternal and zygotic transcriptomes based on stable gene expression

We used the list of stable genes that were expressed at the 4-cell stage to make inferences on which genes may be contributed by the maternal transcriptome. We identified genes that were highly expressed in the oocytes but lowly expressed in human embryonic stem cells (hESCs) and intersected this list of genes with the stable genes that had either medium or high average expression at the 4-cell stage embryos. We also identified genes that were likely to be part of the early zygote transcriptome by looking for stable genes that were either expressed at high or medium average levels at the 4-cell stage, but not highly expressed in the oocytes. We found 90 genes that were likely to be contributed by the maternal transcriptome (S5 Table). For the early zygote genes, we identified 626 genes that were active at the 4-cell stage, and 239 genes that were repressed or lowly-expressed (S6 and S7 Tables).

One of the genes likely to be contributed by the maternal transcriptome that we identified was *DPPA3*, a known maternal factor in the mouse that has a key role in embryonic development. *DPPA3* was actually the only gene that was highly expressed at the 4-cell stage (the remaining 89 genes had medium expression at the 4-cell stage). We also showed that these stable, early zygotic genes were significantly enriched in genes that were known to be targets for the human pluripotency transcription factors SOX2 and NANOG (P-value < 0.05) but not OCT4 [28]. The analyses and results are further described in S4 Text.

### Inferring functional consequences and importance of the genes with stable expression across embryonic development

While we hypothesize that many of the stably expressed genes serve an important regulatory function for human embryonic development, experimentally validating this hypothesis is a significant challenge. Instead, we can use the genome-wide catalogues that have been collected from studies of human disease genes, or genes that represent essential or robust elements of the genome to computationally infer the functional impact of the stable genes that we have identified, on the cell and embryonic development. We conducted a meta-analysis using seven gene catalogues that represent different aspects of essentiality or perturbation of the human genome. The catalogues that were used were the Genome-wide Association Study Catalog (GWAS Catalog) [29], the Online Mendelian Inheritance in Man (OMIM) Gene Map [30], a set of human orthologs of mouse essential genes [31], a set of the top 10% most ubiquitously expressed human genes [32], a set of human haploinsufficient genes [33], a set of human recessive disease genes that had a DNA copy number variant (CNV) deletion [34], and a set of loss-of-function genetic variants in human protein-coding genes [35]. We found that the stable genes were significantly associated with an enrichment of the essential genes and the top 10% of ubiquitously expressed genes (two-sided Fisher’s exact test P-value < 0.05, see S5 Text). The stable genes were marginally significant in overlap with the haploinsufficient genes and the OMIM Gene Map (P-value < 0.10). We also found a significant depletion of stable genes from the loss-of-function variants and the list of recessive disease genes affected by a CNV deletion (two-sided Fisher’s exact test P-value < 0.05). These results provided some evidence to support the claim that the stable genes had important functional consequences for regulation of the cell and by extension, we assume, the embryo. Stable genes were significantly enriched for genes that were essential or ubiquitously expressed, and less likely to be associated with loss-of-function variants or a known recessive disease gene affected by a CNV deletion.

### Identification of developmental stage-specific markers using changes in variability of gene expression

**Fig 5:**
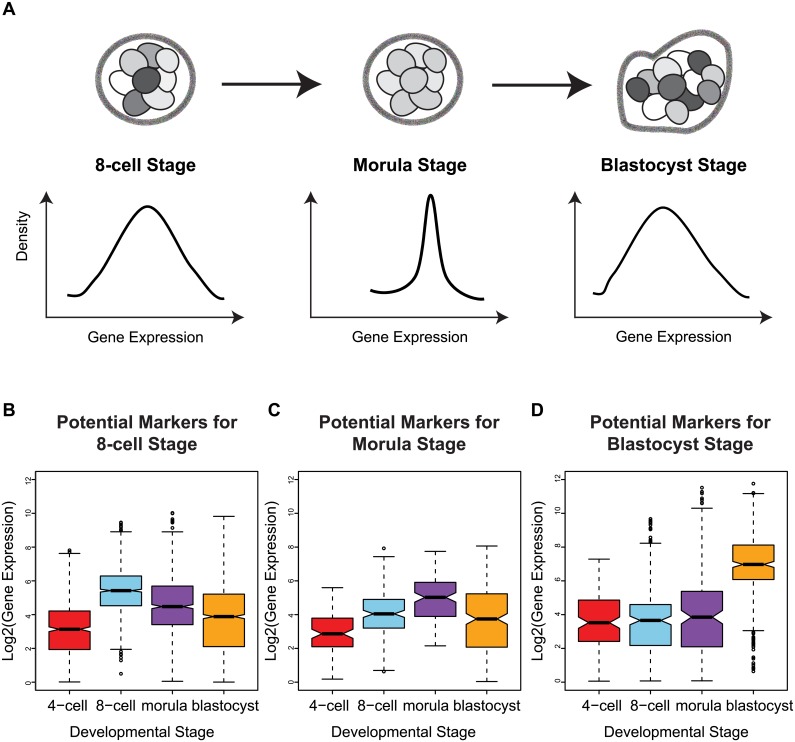
Stage-specific variability markers are based on changes in inter-cellular variability. **(A)** A schematic illustrating how a narrower distribution corresponds to greater homogeneity in the expression of a variability marker in a cell population (the shading indicates level of expression of a gene). **(B-D)** The distribution of expression for the variability markers identified for each developmental stage.

Variability is a statistical property that reflects how the distribution of gene expression in all cells is shrinking or expanding. Genes that have changes in variability at different stages of development may therefore shed light on important stage-specific regulators of the embryo. To identify such genes, we applied the following criteria (1) a gene must have a statistically significant change in expression variability across the four stages based on Levene’s test [36], (2) the minimum level of variable expression for a specific stage (all other stages had a higher SDC), (3) an average expression level at a specific stage that was higher than all other stages (see Fig 5B–5D). To avoid confusion with markers that are found using average-based approaches that are typically employed, we refer to genes satisfying the three criteria listed, as variability markers.

For morulae and blastocyst stages, we identified eight and eleven stage-specific variability markers, respectively (Table 1, Fig 5C and 5D). These genes share a common theme in functioning to ensure the proper development of the embryo, and they mainly play a role in embryonic development, cell protection, or metabolism. For example, mesoderm development candidate 2 (MESDC2), a morulae variability marker, functions as a key mesenchymal chaperone protein for Wnt co-receptors [37] (see S3 Fig). For blastocysts, an example is epithelial cell adhesion molecule (EPCAM), which has a critical role in the formation of trophectoderm, the outer layer of the blastocyst that will eventually become the placental interface [38, 39]. Some of these genes act specifically to protect the cell from free radicals and other toxins, such as blastocyst variability markers peroxiredoxin 6 (*PRDX6*) [40–42] and glutathione S-transferase pi 1 (*GSTP1*) [43]. For single cell expression profiles of the variability marker genes, and functional information from the literature, see S3 and S4 Figs for the morula set, and S5 and S6 Figs for the blastocyst set.

**Table 1:** Putative variability markers identified for each stage based on changes in gene expression variability. Gene names in bold and underlined indicate that these genes that were also identified by a standard ANOVA model.

Stage	Number of Markers	Genes
8-cell	55	*ACBD3*, *BAG5*, *BAZ1A*, *CEP350*, *CSTF3*, ***DIS3***, *DUSP12*, ***FAM122C***, *FBXO5*, *GLA*, *HNRNPF*, *HNRNPH2*, *IFRD1*, *ING1*, *KLF10*, *MFSD11*, *MOSPD1*, ***MRPL16***, *MSH6*, *NAT15*, *NDUFA1*, *NFYA*, *NT5C3*, *NUPL1*, *PANK2*, *PFDN5*, *PIGH*, *PLDN*, *POLR3K*, *PPP4R2*, *PPP6R3*, *PQBP1*, *PRPF4B*, *PTPN9*, *RBBP5*, *RBBP6*, ***REST***, *RFK*, *RLIM*, *RNF115*, *RPS6KA5*, *SENP5*, *SGOL2*, *SHQ1*, ***SNAPC1***, *SPTY2D1*, *STIL*, *TOP1*, *TRIM13*, *UBA1*, *UBE3A*, *UCK1*, *WASF1*, *ZCCHC6*, *ZNF622*
Morulae	8	*ARMC7*, *BMS1*, *CFL2*, *GFOD2*, *MESDC2*, *RPL23AP7*, *SKA1*, ***SNF8***
Blastocyst	11	***ACTN4***, *LAMTOR1*, ***CYP2S1***, ***EPCAM***, ***GSTP1***, ***HDDC2***, ***NDUFA12***, ***PRDX6***, *RPL17*, ***RPL19P12***, ***TMEM147***

When considering the functions that are served by the blastocyst marker genes, one theme was metabolism. In order to ascertain that this was not an artefact of the local environment that the human embryos were stored at, we looked at the expression patterns of the blastocyst marker genes in human induced pluripotent stem cell lines (iPSCs) and hESCs from three other studies [44–46] as well the hESCs that were profiled by Yan *et al*. [25]. For these variability marker genes, we saw high levels of expression relative to the global distribution of expression in all cell lines tested, providing some evidence to suggest that the patterns we observed for the blastocyst marker genes were not likely to be solely a product of the embryo’s environment (see S6 Text).

We also applied an ANOVA model to determine which variability markers could be identified using a standard approach (see S7 Text) and which were specific to our analysis based on gene expression variability. Some of the variability markers had statistically significant P-values from the ANOVA model, and these genes have been highlighted in bold and underlined in Table 1. The largest degree of overlap was observed for the blastocyst variability markers, where only two genes, *LAMTOR1* and *RPL17*, were uniquely detected based on our variability-based approach.

### Functional validation of *HDDC2* expression suggests *HDDC2* plays a role in the maintenance of pluripotency of human iPSCs and ESCs

**Fig 6:**
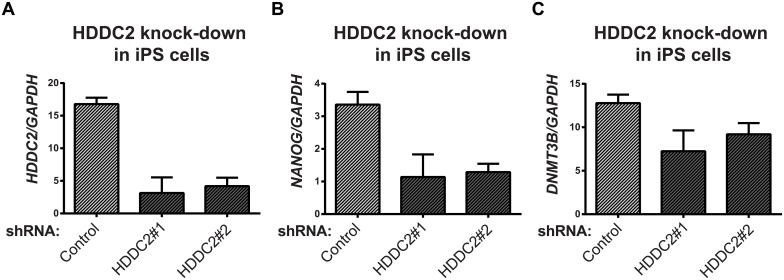
Effect of *HDDC2* shRNA-mediated knock-down. **(A)** Decrease in *HDDC2* mRNA levels after 2 days of constitutive expression of shRNAs (see S11–S13 Tables). **(B)** Corresponding drop in the expression of pluripotency markers *NANOG* and **(C)** *DNMT3B* suggests a role for *HDDC2* in the maintenance of pluripotency.

**Fig 7:**
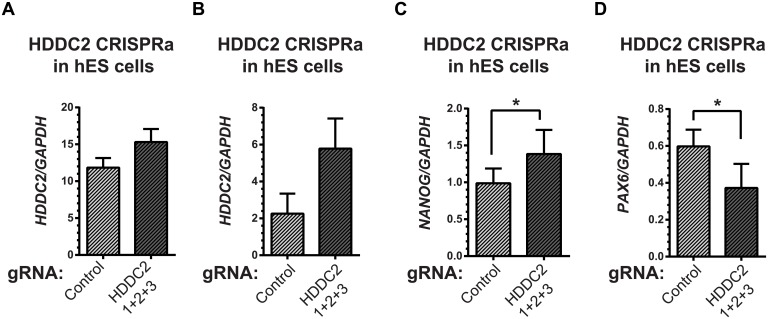
Activation of the endogenous *HDDC2* locus using an inducible Cas9-VP64 system attenuates neural differentiation of human pluripotent stem cells. **(A)** Upregulation of the *HDDC2* mRNA levels after 2 days of induction of activation in pluripotent hES cells. **(B-D)** Effect of Cas9-VP64-driven *HDDC2* up-regulation on gene expression during the early stages (day 3) of neural differentiation. We observed that artificially-maintained levels of *HDDC2* expression **(B)** resulted in more sustained *NANOG* expression **(C)** and lower induction of *PAX6* **(D)**, a definitive neuroectodermal marker.

To demonstrate the functional impact of the stage-specific variability markers, we validated one of the blastocyst variability markers, *HDDC2*, by shRNA-mediated knockdown in a human iPSC line. qPCR experiments confirmed that the knockdown of *HDDC2* mRNA caused a significant decrease in the expression of key pluripotency markers *DNMT3B* and *NANOG*, two genes that are critical for embryonic development and maintenance of pluripotency in iPSCs (see Fig 6). We also tested the impact of the up-regulation of the *HDDC2* locus on hESC differentiation using a hESC line with a stably-integrated inducible CRISPRa/Cas9-VP64 artificial transcriptional activator system. Over-expression of *HDDC2* attenuates the drop in expression of the pluripotency marker *NANOG* and induction of the neuroepithelial marker *PAX6* during the early stages of neural differentiation (see Fig 7). While these experiments cannot provide evidence for the effects of *HDDC2* on cell viability or embryonic development, the results from the *HDDC2* knockdown suggest that this gene plays a role in the maintenance of pluripotency. From the transcriptional effects caused by *HDDC2* over-expression on early neural differentiation, we can infer that *HDDC2* is able to either reinforce the persistence of the pluripotent phenotype, or is involved with specific interference of the neural differentiation process, or both.

### The embryonic transcriptome is under different levels of regulatory control during development

In early developmental processes, evidence supports the role of gene expression variability as a necessary initiating step that precedes lineage commitment in progenitor cells [8, 47, 48]. As embryos transition from the 4-cell to the blastocyst stage, the global distribution of inter-cellular variability shifts from lower to higher values (Fig 3A). Based on the shape of the density distribution, we see an increase in the overall number of genes that are expressed more heterogeneously between cells as the embryo differentiates. This result likely reflects the diversification of transcriptomes observed in cells that are undergoing the necessary cell fate changes to become distinct lineages and specialized cell types [49] but which also contain inherent stochasticity [50].

**Fig 8:**
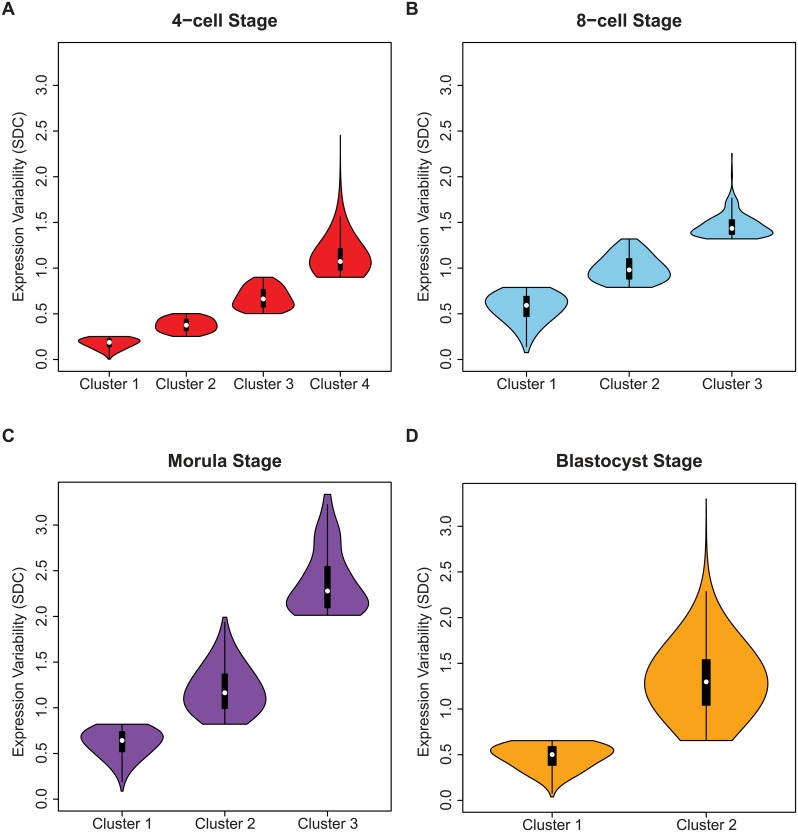
Identifying regulatory control states. Genes were assigned to clusters using a mixture model algorithm for each developmental stage based on levels of inter-cellular variability. The goal was to use this algorithm to quantify how many different clusters, or regulatory control states were present for each stage. The mixture models identified **(A)** four clusters for the 4-cell stage, (**B)** three clusters for the 8-cell and **(C)** morulae stages, and **(D)** two clusters for the blastocyst stage. In **(B-D)** the distribution of the inter-cellular expression variability are represented for each cluster or control state per stage.

The shape of the inter-cellular variability distribution demonstrates how at each developmental stage, the transcriptome is expressed with levels of precision that span a wide spectrum (Fig 3A). This is unsurprising given that during the 4-cell to the 8-cell stage, the embryo undergoes activation of the zygotic genome (ZGA) and a massive reprogramming of the transcriptome occurs [51, 52]. The stochastic nature of reprogramming has been observed in stem cells as well as the existence of alternative stem-cell states, and hence we expected to see higher levels of SDC [53]. Analyzing expression variability gives us a window into which components of the transcriptome are being used in cells of the developing embryo at different levels of precision. We are interested therefore in characterizing how precision changes as the embryo develops, and which genes are involved at either end of the regulatory control spectrum. We refer to these different levels of expression variability as control states. Mixture models were applied to identify within the data, the number of control states present at each stage and classify the subsets of genes observed under different levels of regulatory control (see Fig 8, Materials and Methods). The 4-cell stage had the largest number of control states, where each state could be interpreted as one of four distinct levels of variable expression (low, medium, high, very high) (Fig 8A). During development, we observed that these levels collapse down into simpler states, e.g. for the morula stage, there are three levels (see Fig 8C) and for the blastocyst stage, there are only two (Fig 8D).

### Stage-specific genes with different levels of inter-cellular variability are enriched for distinct pathways and processes

We investigated whether genes with the same level of regulatory control were enriched for certain pathways or processes using IPA software. Much like the variability markers, we observed consistent changes in pathways that appear to protect and ensure adequate gene regulation in the developing embryo (S8A–S8D Table). For example, four pathways were enriched for low-variability (most stable, mixture 1) genes, EIF2 signaling, regulation of eIF4 and p70S6K, mTOR signaling, and protein ubiquitination. This overlap was significant for all four developmental stages, and represents pathways that control important functions that are required by every living cell (for an overview of the important enriched terms occurring per stage, see S7 Fig).

For the 4-cell stage only, the low-variability genes were enriched for the “DNA Methylation and Transcriptional Repression Signaling pathway” (S8A Table). The enriched genes (*SIN3A*, *SAP18*, *SAP130*, *RBBP7*, *RBBP4*, *CHD4*) were involved in regulation of histone deacetylation, and DNA methylation (*DNMT1*). Proper regulation of histone deacetylation is known to be a requirement for embryonic development [54]. DNMT1 is involved in maintaining single-stranded methylation of newly replicated DNA, and its expression is activated by cell cycle-dependent transcription factors in the S phase [55]. We also observed enrichment of a key developmental pathway “Ephrin B signaling” for the 4-cell low-variability group but at no other stage. Genes enriching this pathway have been directly implicated in fish and mammalian embryo development, including Rho-associated, coiled-coil containing protein kinase 2 (*ROCK2*), beta catenin (*CTNNB1*) and ras homolog family member A (*RHOA*) [56–58].

Pathways associated with telomere extension by telomerase, and telomere signaling were statistically significant amongst variably-expressed genes at the 4-cell and the blastocyst stages (mixtures 3 and 2 respectively, S8A and S8D Table). The importance of telomerase to preimplantation embryos has previously been established via its link to reproductive potential [59]. At the 4-cell stage, telomeres are thought to reset during genome activation, which may explain the higher number of telomerase-associated genes that have variable expression at this stage. The six genes that were annotated to the telomerase extension pathway by IPA (*TERF2*, *TNKS*, *HNRNPA2B1*, *TERF2IP*, *TNKS2*, *POT1*, *XRCC5*, *RAD50*, and *NBN*) have roles in double-strand DNA break repair and damage response, where they are responsible for protecting the telomere extension process. Telomere DNA elongation has been observed to occur between the 8-cell and the blastocyst stages in both animal [60, 61] and human embryo studies [62]. It is likely that this corresponds to the second enrichment of variable genes observed at the blastocyst stage. Although successful telomerase elongation is critical to the embryo, variability in expression of genes associated with this process may be due to the slightly different rates at which cells are using and completing this process.

The transition from the morula to the blastocyst features the appearance of the first cell type specification with the organization of the trophectoderm. Integrin binding and activation is an essential element of blastocyst implantation and trophoblast differentiation [63–65]. Integrins are a class of heterodimeric transmembrane cell surface receptors that participate in cell-cell interactions and regulate signals for cell adhesion, growth and survival. We found that genes in the IPA pathway “Integrin Signaling” first appeared in the gene clusters for the morula stage, and continued to be significantly enriched in the blastocyst stage, possibly indicating the variable process of cell differentiation within the morula before the visible segregation of the trophectoderm (S8C and S8D Table).

For the blastocyst stage, we find that low-variability genes were enriched for metabolic pathways, such as oxidative phosphorylation, oxidative stress, and glycolysis-related pathways (S8D Table). In fact, glycolysis and gluconeogenesis pathways are uniquely observed to occur for low-variability genes in the blastocyst stage. This may indicate the appearance of a more diverse regulatory program in blastocyst metabolism. Blastocysts are known to require a higher metabolic load due to the burst in developmental change that occurs at this stage. For example, the cytosolic form of malate dehydrogenase (*MDH1*), which is featured in both oxidative phosphorylation and gluconeogenesis pathways has been shown to be important for blastocyst development in mice [66]. On the other hand, by the blastocyst stage, because of the emergence of distinct lineages and cell types, cells across the embryo may begin to show signs of asynchronicity in cell cycling. We see this effect reflected in the statistically significant enrichment of cell-cycle related processes in variably-expressed genes at the blastocyst stage. Dependence on specific developmental and signal transduction pathways is also expected to be more heterogeneous at this stage owing to the distinct cell types that have different expression programs. Variably-expressed genes at the blastocyst stage were enriched for several signaling pathways, including ATM signaling, PI3K/AKT signaling, JAK/Stat signaling, ERK/MAPK signaling, and others (S8D Table).

### Understanding cell population heterogeneity by sampling subsets of cells

**Fig 9:**
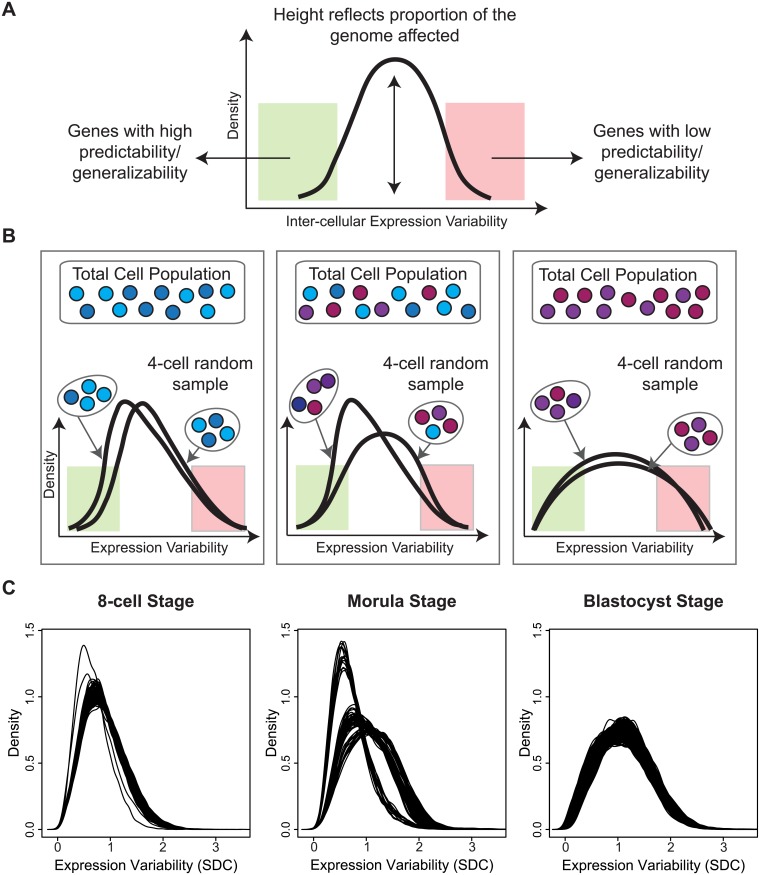
A snapshot of cellular heterogeneity across embryonic development. **(A)** The variability profile for a group of cells identifies both the regions and proportions of the transcriptome that have different levels of expression variability. **(B)** A schematic diagram showing how different densities can arise depending on how heterogeneous the underlying cell population is. (**C)** Observed densities for the 8-cell, morula and blastocyst stages where each line corresponds to a 4-cell combination selected from the total cells available in each developmental stage.

While our analysis so far has concentrated on identifying the gene-specific measures that vary in a cell population (see Fig 9A), it is worthwhile to highlight those cells that are the most aberrant or consistent in the population, with respect to global gene expression. Sampling a fixed number of cells and analyzing patterns of variability allowed us to look at the overall heterogeneity of the cell population based on the global gene expression variability distributions. Inspection of the density plots obtained for each 4-cell sample revealed interesting properties that provide insight into the transcriptional noise occurring at a specific development stage (Fig 9B). Multiple draws of cells from the population can highlight how good of a representation any single cell sampled at random is likely to be. For situations where it is not possible to sample every cell in an organism or tissue, this kind of analysis gives us the ability to assess which parts of the transcriptome are more generalizable and stable (Fig 9B), and conversely, which cells may be unusual or aberrant.

For the 8-cell stage, we observed that all 4-cell combinations produce overlapping densities and suggest that most cells are relatively similar to each other in the embryo at this stage of development with respect to global variability (Fig 9C). The shape of the densities also indicate that most of the transcriptome is expressed with lower variability. For the morulae stage, it is clear that there are three dominant sub-clusters of 4-cell combinations and that this stage has the greatest degree of cellular diversity. For the blastocyst stage, this diversity appears to subside, and cells are more homogeneous with respect to their overall variability profile. The density for the blastocyst combinations suggest that although there is reduced cellular heterogeneity compared to the morulae stage, there are more genes expressed at higher levels of inter-cellular variability. These densities may reflect a transition signature of the morula cells. In blastocysts, there are at least two major tissue types (trophectoderm, inner cell mass) whereas the morulae are still in an undifferentiated state where cells are only starting to transition into two different types. The blastocyst, on the other hand, is where we see the first differentiation events occurring.

### Validation of key results using two mouse embryo developmental studies

One limitation of the human embryo data set was that it featured relatively small numbers of embryos, and hence to test the robustness of our results, we looked to two separate studies that were performed in mouse as a source of data to validate our key findings (see S8 Text). The first study from Guo *et al*. [7] generated gene expression data for 48 genes in five to seven embryos per stage using the Fluidigm Biomark System 48.48 Dynamic Arrays. We selected the data from the 4-cell, 8-cell, 16-cell, 32-cell and 64-cell stages as these paralleled the most closely with the human developmental stages that were used in our analysis of the human embryos. Using the Guo data set we were able to verify the existence of a subset of stably expressed genes that had invariant expression across the development of the embryo, and these genes showed low, medium and high levels of average expression. We also detected sets of variability markers that displayed changes in average expression and expression variability that appeared to delineate a specific stage of development. Although only 48 genes were profiled in this data set, we saw that the distribution of expression variability for the total set of genes adopts wider values as the mouse embryos developed. Even with a higher number of mouse embryos, the distribution of inter-embryo variability remained relatively constant as we had observed with the human data.

In the second mouse study, Deng *et al*. [67] applied Smart-seq and Smart-seq2 single cell RNA-sequencing to profile the transcriptomes of cells taken from early stage embryos. Their experimental design was similar to that of the Yan human embryo data, but with a higher number of embryos and cells. We used the 4-cell, 8-cell, 16-cell and late blastocyst stages from their study to compare our results. Our analysis of the Deng data set also yielded results that aligned with those observed from our study of the human embryos. Moreover, because the number of genes in both data sets was more similar, we were able to compare the overlap between the lists of stable and variable genes that were identified in the mouse and human embryos. The overlap was statistically significant (two-sided Fisher’s exact test P-value < 10^−23^, odds ratio estimate 3.744), even after permutation tests (see S8 Text). We tested the gene lists from the human-mouse comparisons for enrichment of biological pathways and processes using the MSigDB database [68] (based on Hallmark gene set terms and C4 computational gene set terms) to further investigate the nature of both the overlap between the human and mouse stable genes, but also the species-specific differences. Genes that were stable in both human and mouse embryos, were enriched most strongly for MYC targets and housekeeping genes. We found that the discordant genes (either stable in mouse but variable in human, or vice versa) were enriched for more disease-specific terms and processes. Two terms that were unique to the list of genes variable in human but stable in mouse, reflected gene sets that regulate pluripotency (the Wong Embryonic Stem Cell Core [69] and the Mueller PluriNet protein-protein interaction network [70]). Based on these results, genes that are discordant with respect to expression variability may play a role in human-specific (or mouse-specific) diseases, or be required for species-specific embryonic development and maintenance of pluripotency.

## Discussion

We have taken a novel approach to uncovering how the transcriptome is regulated in human embryos. The use of technologies enabling analysis with a single cell resolution enables an embryo to be modeled as a defined population of cells, and by studying variability between these cells directly, we can identify genes whose expression is either homogeneously or heterogeneously distributed in the cell population. Our analyses have highlighted key developmental pathways that show different degrees of regulatory control, and we have demonstrated that analyses of expression variability can provide functional insights into stage-specific markers. It is important to emphasize that all our inferences on regulatory control of the transcriptome are based on data collected from human embryos. This has an impact on improving our understanding of transcriptional regulation in human developmental processes. A recent study has revealed that even for mice, a species that is often believed to be a good model of human biology, the differences in transcriptomes from humans are larger than expected [71]. In embryology, there are many technical, ethical and scientific limitations that understandably make the use of non-human embryos more feasible for—omic studies; however, it is useful to keep in mind that the results of our study provide valuable insights into how the transcriptome is controlled and used during the development of an actual human embryo.

The greater diversity of control states observed for the 4-cell stage is likely to be a result of the transcriptome being in a continued state of flux and reorganization following zygotic genome activation (ZGA). As the embryo transitions away from the 4-cell stage, plasticity in global gene expression gives way to the commencement of the more specific differentiation programs. This phenomenon may overlap with what other studies have described as “waves of transcriptional activation” [52]. We see genes in the blastocyst stage segregate to one of two different control states (see Fig 8D), and from this, we speculate that control of the transcriptome has polarized into low and high levels of precision, with fewer intermediately-variable genes observed than in earlier developmental stages (see Fig 8A and 8B).

The decreased complexity of regulatory control observed across development is also consistent with our model of how ZGA is influenced by the number of completed cell cycles. Our general understanding is that repressors and activators have opposing activity during ZGA that changes as cells of the embryo transition through successive cell cycles [72]. During early embryogenesis, the expression activity of maternal repressors is high at first but as these cells divide further their concentration is diluted in the embryo and therefore this activity eventually decreases. Activators, on the other hand, are initially present at low levels and may require successive cell cycles before attaining a threshold level for successful activation. The interplay between activators and repressors gives rise to the resulting levels of mRNA copies, and associated degree of heterogeneity observed in the embryo as it develops.

We identified genes with stable expression for the 4-cell to blastocyst stages, and these may form the core set of genes required for embryonic development to occur successfully. The stable genes were expressed at one of three distinct modes that spanned low, medium and high levels of expression, and this is interesting to note because it suggests that invariance of expression may be part of regulating genes required at all expression levels, and is not only confined to genes that are highly activated or need to be silenced. The assumption that variability diminishes inversely with the absolute level of transcription is not consistent with our observations. The first two modes of expression (low and medium) could be interpreted as genes that are turned off and on, respectively. Stable genes with low expression may be the result of a gene that has been switched off and their non-zero expression is the result of background signal observed in the RNA-seq experiment. Alternatively, these genes may be weakly expressed due to other mechanisms, e.g. from leaky transcription, where initiation of transcription occurs but not enough of the necessary components from the remaining machinery are available to follow through. The third expression mode (stable genes with high expression) may operate as accelerators or as higher level activators that support or function in roles related to the stable genes with medium expression. The fact that the pathways with significant enrichment for these genes overlap with those enriched for the medium-expressed (Fig 4), stable genes supports this interpretation.

Identifying genes based on their lack of variability has some parallel to developing catalogues of housekeeping genes, a concept that has been a fixture in molecular biology for nearly fifty years now [73]. While housekeeping genes represent a practical use in standardization and quality control, they have also garnered interest from a systems biology perspective because they represent a core, minimal set of genes necessary to support the cell. With growing technology, we continue to revise our definition of housekeeping genes; however, as far as we are aware, none of these approaches evaluate changes in variability directly. Our approach therefore has cross-over utility for the purpose of identifying potentially new housekeeping-type genes in single cells. Similarly, our approach may also have utility for defining stage-specific control genes that can be used to normalize or calibrate expression data of other genes with more heterogeneous expression.

We compared the stable genes that showed low, medium and high expression across all stages (see Fig 4 and S1 Table) with two sources of housekeeping genes, the top 10% of ubiquitously expressed genes collected by de Jonge *et al*. [32], and a list of housekeeping genes for RNA-seq data collected by Eisenberg *et al*. [73]. For both sets of housekeeping genes, we saw considerable overlap between the stable genes that had medium or high levels of expression compared to very few lowly-expressed stable genes (S9 and S10 Tables).

Across species, the property that divergence occurs at early and late, but not the middle part of development trajectories is a phenomenon referred to as the hourglass effect [74]. Other studies have shown that developmentally important genes demonstrate remarkable sequence similarity across a wide range of organisms, pointing to shared mechanisms that have been evolutionarily conserved. It has also been shown that gene expression control follows a similar pattern of conservation or stability, and experimental evidence to that effect has been most recently reported by Gerstein *et al*. [75]. Using transcriptomes of distant species generated by the ENCODE and modENCODE consortia, genes exhibiting developmental stage-specific expression showed diminished inter-species expression variability in the middle stages of development but were heterogeneous before and after. A recent paper by Liu *et al*. [76] also analyzed the same human embryo data set generated by Yan *et al*. [25] where they built co-expression gene modules and performed evolutionary conservation analysis on gene sequences as well as transcriptional regions upstream of each gene. They concluded that genes from stage-specific co-expressed modules have increased selective pressure at the 8-cell stage versus earlier stages (zygote to the 4-cell stage) and later (8-cell to the late blastocyst stage). In our study, the variability marker genes that were identified for the 8-cell and the morula stages also follow a pattern of diminished variability at these intermediate development stages (see Fig 5B–5D). This observation corresponds to Liu *et al*.’s discovery of increased selective pressure in co-expressed module genes at the 8-cell stage. Higher evolutionary pressure represents an increase of evolutionary constraint. Both independently derived observations point to an ‘hourglass’ in the preimplantation embryo, with lower evolutionary constraint and higher variability flanking a period of higher evolutionary constraint and lower variability.

The stage-specific variability markers we have identified show promise as potential determinants of embryo stage, however, they also shed light on understanding embryogenesis. It is likely that these markers are consistently expressed for all cells of the embryo because they provide or support critical functions for the developing embryo. This hypothesis could be readily tested in the context of model organisms like zebrafish and Drosophila. For human cells, these markers may be useful in future studies for identifying healthy embryos based on transcriptional states. Some of these applications may be in laboratory embryology where analysis of the least variable components of the transcriptome may serve as a scaffold to create predictive signatures of the developmental stage and potential of a biopsied embryo’s cells, especially for expression effects in genetically modified embryos. In conjunction with other existing criteria, information based on expression variability may eventually be used as a screen during routine day 3–5 biopsy for preimplantation embryos in IVF and other applications to improve pregnancy rates of women using these procedures.

We also see potential clinical applications in genetic diagnosis for reproductive potential for human embryo pre-implantation. Embryos that are otherwise chromosomally healthy are known to have a significant risk of implantation failure [77]. While there are many reasons underlying implantation failure, one significant cause may be due to innate errors in development due to disordered regulatory programs in the developing embryos that may result from genetic, epigenetic and environmental factors. As trophectoderm biopsies taken on day 5 (blastocysts) are regularly used as material for preimplantation genetic diagnosis (PGD), part of the biopsy material could be used to isolate cells and test the transcriptional regulatory state of the embryo in the trophectoderm for both expression level and SDC. This effect could be studied in a clinical trial through genetic fingerprinting analysis, determining the origin of a single birth following a double embryo transfer into the same patient. The expression state of cells in the implanting embryos versus the non-implanting embryos can be quantified and compared among a cohort, including the SDC of genes that represent important biomarkers for embryo reproductive potential.

There is value to understanding the variability of genes as a marker of cell state and regulatory potential [19]. Other variability markers may also unlock intriguing clues into how blastocysts are regulated (see S5 and S6 Figs). NDUFA12 helps to generate ATP for the cell via oxidative phosphorylation in mitochondria [78]. Mutations in this gene are often the cause of mitochondrial oxidative phosphorylation diseases, suggesting that NDUFA12 is critical for maintaining healthy human cells, and for blastocysts, NDUFA12 may play an important role in supplying these cells with sufficient energy [79]. LAMTOR1 is a membrane protein whose function is currently only known in the context of late endosomes and lysosomes, which are organelles that the cell relies upon to perform waste disposal. LAMTOR1 is anchored to the surface of these organelles, and through other intermediates can activate the mTORC1/MAPK signaling pathways that lead to cell growth and control of energy homeostasis [80]. At this point, it is difficult to say what impact LAMTOR1 has on blastocysts; however, a mouse study has shown that LAMTOR1 is responsible for proper epidermal development by regulation of lysosome-mediated catabolism [81]. It is possible that these processes may affect other tissues in the blastocysts more generally, and hence we see this gene being flagged as a variability marker.

Another marker of interest, ACTN4 is part of the α-actinin family, a set of related proteins that bind a wide range of molecules, including actin, to regulate a host of important processes in the cell [82]. α-actinin appears to operate in cell type-specific roles in the human body. Specifically, ACTN4 has been found to be ubiquitously expressed and distinct from the other α-actinins in that it interacts with transcription factors [83]. While its connection to blastocysts is also unclear at this point in time, ACTN4 has been detected in higher levels in cells with higher motility [84] and may be linked to metastatic processes in cancer [85, 86]. Motility in cells of the blastocyst may be important for blastocyst activation and trophectoderm motility [87] as previously observed in mouse embryos.

We observed that some of the variability markers that were identified using our approach overlapped with markers that can be found with standard approaches such as an ANOVA model. One example was EPCAM, a blastocyst variability marker identified also by ANOVA (see S5 and S6 Figs). EPCAM acts as a cell surface protein that is often used as a stem cell marker [88] because of its association with elevated levels in undifferentiated human embryonic stem cells [89]. Depending on the cell type that the expression profile was captured in, and other biological factors, there may be genes showing distinct changes in variability and/or average expression and both statistics are informative. Decreases in variability may be indicative of increased criticality, whereas increases in average expression may suggest a general trend of the cell population.

While transcription is fundamentally a stochastic phenomenon that affects all genes generically, the observation that some genes are more variably expressed than others, points to the existence of physical mechanisms underlying gene expression variability. These mechanisms can be grouped broadly into two major sources of variation. The first source refers to mechanisms embedded in the genome sequence, where the content of the promoter region and other regulatory sequences affects the degree of expression variability of a downstream gene. Nucleosome positioning, for example, is one such mechanism that can generate variable gene expression where a nucleosome typically occupies a region where transcription start sites and TATA elements exist [90]. Another example involves the interaction of long-range gene regulation where the expression of some genes require the interaction of other chromosomal segments. This mechanism can generate increased expression variability for genes that are regulated by this kind of nuclear architecture [91]. Other epigenetic mechanisms such as the presence of chromatin modifications have also been attributed to expression variability [92].

The second source of variation refers to the networks or interactions that a gene participates in that can influence its expression variability. We have seen through both experimental and simulation studies, that certain circuit formations, such as auto-regulatory motifs and feedback loops, can propagate signals to create different degrees of noise or variability [15, 16]. Pathways can be viewed as a collection of integrated circuits of genes, and the makeup of these circuits have been refined through evolutionary processes to become highly gene-specific and well-tuned. Therefore, depending on the placement of genes in their specific circuits, they may be more highly sensitive to exhibit fluctuations in their expression [93]. Related to this is the interaction with other molecules that have repressive or activator effects on a mRNA signal. For example, the interaction with microRNAs and shRNAs in the cell may deplete transcript levels of a gene however due to concentration, spatial regulation and other intracellular effects, this repression is likely to be observed non-uniformly in a cell population.

## Materials and Methods

### RNA-seq data

We used the RPKM (reads per kilobases per million reads) normalized RNA-seq data set generated by Yan *et al*. [25]. The data was downloaded from GEO using the accession number GSE36552. Whole-genome RNA-seq data from three embryos were available for the four embryo stages used in our study (4-cell, 8-cell, morula, blastocyst) except for morula where there were only two embryos. For morula and blastocyst, eight and ten cells were profiled respectively. In order to eliminate genes that may be affected by poor quality, genes with an RPKM-expression value ≥ 0.1 in at least 75% of cells from the same stage were retained. This resulted in 8105 genes that were used for further analysis.

### Variability measures

To measure the inter-cellular expression variability of each gene *g*, we adopted the following statistic *SDC*_*g*_:

$$SDC_{g}=\frac{1}{E}\overset{E}{\underset{j=1}{\sum }}\sqrt{\frac{1}{N_{j}}\overset{N_{j}}{\underset{i=1}{\sum }}{\left(x_{ij}-{\bar{x}}_{j}\right)}^{2}}$$

where *x*_*ij*_ is the expression level of gene *g* in the *i*-th cell of the *j*-th embryo for a total number of *E* embryos. For each embryo *j*, there are a total of *N*_*j*_ cells that have been profiled. ${\overset{-}{x}}_{j}$ represents the average expression occurring in the *j*-th embryo. The SDC captures the SD of expression levels observed between cells belonging to the same embryo, that is then averaged across all embryos to give an overall measure of inter-cellular variability. We make the assumption that the embryos represent biological replicates, and therefore genes showing consistent inter-cellular variability between embryos are given higher weight by the *SDC*. This assumption is reasonable to make given that the embryos were screened extensively and only those passing stringent morphological criteria were included for RNA-sequencing (Fig 1).

As a contrast to the *SDC*, and as a means to evaluate inter-embryo variability of expression, we also computed the *SDE*_*g*_:

$$SDE_{g}=\sqrt{\frac{1}{E}\sum_{j=1}^{E}{\left[\frac{1}{N_{j}}\sum_{i=1}^{N_{j}}{\left(x_{ij}-{\bar{x}}_{j}\right)}^{2}-\frac{1}{E}\sum_{j=1}^{E}\frac{1}{N_{j}}\sum_{i=1}^{N_{j}}{\left(x_{ij}-{\bar{x}}_{j}\right)}^{2}\right]}^{2}}.$$

All analysis and code is available upon email request.

### Mixture modeling to identify different variability states at each stage

For each stage, the SDC values were clustered using Normal mixture models to identify how many variability states were present. We used the Mclust function in the **mclust** R package (version 5.0.1) where the number of states was inferred by the function, and selected from possible values ranging from 1 to 9.

### Functional enrichment analyses

We used the Ingenuity Pathway Analysis database (Summer Release 2014) to identify significantly enriched pathways and processes for genes in different variability states at each stage. Significance was determined using a Fisher’s exact P-value that was adjusted for multiple testing correction using the Benjamini-Hochberg method [94]. Cut-offs were applied and are specified in the corresponding table legends.

### Identifying stage-specific variability markers

We applied three simultaneous filters to identify stage-specific variability markers. To qualify as a marker of stage X, a gene had to have (1) the smallest SDC in stage X compared to all other stages; (2) a higher average expression in stage X compared to all other stages; (3) a statistically significant change in variability as detected by Levene’s test (adjusted P-values < 0.05).

### Experimental validation of *HDDC2* in human iPS and ES cell lines

For the knockdown experiments, a transgene-free human iPS cell line (clone C11 [95]) was transduced with lentiviral particles delivering pLKO.1-Puro family vectors constitutively expressing shRNAs against either control (EGFP) or *HDDC2* (x2) mRNAs (see S11–S13 Tables). Puromycin selection (2μg/mL in the culture medium) was applied at days 4–6 after transduction, after which RNA was harvested for qPCR analysis.

For inducible up-regulation experiments, a human ES cell line inducibly expressing Cas9-VP64, an artificial transcriptional activator, was transduced with lentiviruses allowing for a puromycin-selectable expression of chimeric gRNAs targeting either unrelated gene or HDDC2. 2 days after the start of puromycin selection commencing at day 4 after transduction, induction of gene activation was started by addition of doxycycline at 1μg/mL. 2 days after that neuronal differentiation was started using a standard dual Smad inhibition protocol (on days 0 and 2 of differentiation, KOSR medium without FGF but supplemented with 5μM SB431542, 10 5μM Dorsomorphin and doxycycline at 2μg/ml). RNA was harvested for qPCR analysis on day 3.

### Analysis of stable gene expression

We first identified genes that failed to meet statistical significance for Levene’s test (adjusted P-value > 0.05). These genes were clustered according to their SDC values into three groups that corresponded to low, medium and high levels of variability. We used the hclust function to perform agglomerative hierarchical clustering. Functional enrichment analysis was performed on the low variability group using IPA software. The set of stable genes were taken to be the cluster with the lowest level of variability (Cluster 1, S1 Fig).

### Assessing dependency of sample size and inter-cellular variability

All possible 4-cell combinations were elucidated for each stage, and the variability profile constructed for each combination.

## Supporting Information

S1 Fig Identification of stable genes from the data. Genes with no significant change in variability across development (as identified by Levene’s test, where the adjusted P-value was not significant, i.e. P-value > 0.05) had their expression variability clustered using hierarchical clustering. The goal was to understand the different levels of variability present for genes that showed no significant changes during development. **(A)** Schematic outlining our approach for identifying the stable genes. (**B)** We identified three clusters, and their **gene expression** values are represented in the heatmap where colors correspond to their value. Each square represents expression averaged over all cells for a gene. (**C)** The expression variability was averaged for all genes in the same cluster, and average profiles of the clusters span low to high levels of variability. Given that genes in Cluster 1 had the lowest level of variability across development, we identified these as stably expressed genes.
(EPS)

S2 Fig Examples of stable genes with medium levels of absolute expression. These genes have stable (invariant) expression and are expressed at medium levels at all stages of development. Each black dot represents expression of the gene in a single cell, the red dots represent average expression for that gene over all cells in a specific stage.
(EPS)

S3 Fig Variability markers identified for the morula stage. Each black dot represents the expression of the gene in a single cell, the orange dots represent expression that has been averaged across all cells.
(EPS)

S4 Fig Functional information from the literature about the morula variability markers identified in our study. **(A)** A diagram representing the role of the morula variability markers, and where possible, their relevance to embryonic development. **(B)** References associated with the functional information reported in the literature.
(EPS)

S5 Fig Variability markers identified for the blastocyst stage. Each black dot represents the expression of the gene in a single cell, the blue dots represent expression that has been averaged across all cells.
(EPS)

S6 Fig Functional information from the literature about the blastocyst variability markers identified in our study. **(A)** A diagram representing the role of the blastocyst variability markers, and where possible, their relevance to embryonic development. **(B)** References associated with the functional information reported in the literature.
(EPS)

S7 Fig Summary of enriched terms based on each developmental stage. This cartoon provides an overview of the significantly enriched pathways and processes as they appear for specific developmental stages, and also whether they were enriched for genes with low or high expression variability.
(EPS)

S1 Text Inspection of the RNA-seq data set to ensure appropriate data quality for studying gene expression variability. (DOCX)

S2 Text Investigating the impact of potential aneuploidy on inter-cellular expression variability. (DOCX)

S3 Text Characterizing the statistic to measure gene expression variability. (DOCX)

S4 Text Distinguishing the contributions of the maternal and zygotic transcriptome to the developing embryo. (DOCX)

S5 Text Assessment of the functional impact of expression variability on human embryonic development. (DOCX)

S6 Text Inspecting the expression of the blastocyst variability markers in human iPSCs and ESCs from other studies. (DOCX)

S7 Text Comparison of stage-specific variability markers and the genes identified using a standard ANOVA approach. (DOCX)

S8 Text Validation of key results using two single cell mouse embryo data sets. (DOCX)

S1 Table The number of stable genes identified in each expression mode for a specific developmental stage. A subset of stable genes were also common to all stages.
(DOCX)

S2 Table Over-representation of IPA Function Annotation Terms for stable genes with low expression. The IPA Function annotation terms that were enriched in the list of stable genes with low expression. Criteria for statistical significance was adjusted P-value < 0.05, and number of molecules per term ≥ 10.
(DOCX)

S3 Table Over-representation of terms for stable genes with medium expression using IPA (A) Pathway Annotation terms and (B) Function Annotation terms. **(A)** The IPA Pathway Annotation terms that were enriched in the list of stable genes with medium expression, where criteria for statistical significance was adjusted P-value < 0.01. **(B)** The IPA Function Annotation terms that were enriched in the list of stable genes with medium expression where criteria for statistical significance was adjusted P-value < 0.05, and number of molecules per term ≥ 10.
(DOCX)

S4 Table Over-representation of IPA Pathway Annotation terms for stable genes with high expression. The IPA Pathway Annotation terms that were enriched in the list of stable genes with high expression where criteria for statistical significance was adjusted P-value < 0.01.
(DOCX)

S5 Table List of genes that are likely to be contributed by the maternal transcriptome. (XLSX)

S6 Table List of genes that are likely to be contributed by the early zygotic transcriptome and transcriptionally active. (XLSX)

S7 Table List of genes that are likely to be contributed by the early zygotic transcriptome and transcriptionally repressed or lowly expressed. (XLSX)

S8 Table Over-representation of IPA Pathway Annotation terms for genes at different levels of variability in the (A) 4-cell stage, (B) 8-cell stage, (C) morula stage, and (D) blastocyst stage. The IPA Pathway Annotation terms that were enriched in genes for each level of variability identified (mixture 1 denotes the cluster of genes with the lowest level of variability, mixture 4 is the cluster with the highest level of variability). Criteria for statistical significance was adjusted P-value < 0.01.
(DOCX)

S9 Table The number of stable genes that are in common to all developmental stages and their overlap with the top 10% of ubiquitously expressed genes from de Jonge *et al*. (DOCX)

S10 Table The number of stable genes that are in common to all developmental stages and their overlap with the housekeeping genes identified by Eisenberg *et al*. (DOCX)

S11 Table shRNA target sequences used in validation of *HDDC2*. (DOCX)

S12 Table qPCR primers used in validation of *HDDC2*. (DOCX)

S13 Table *HDDC2* gRNA target sequences. (DOCX)
