# Supplementary material for: Variability of Gene Expression Identifies Transcriptional Regulators of Early Human Embryonic Development
Source: PLoS Genet. 2015 Aug 19;11(8):e1005428. doi: 10.1371/journal.pgen.1005428 (PMC4546122; doi:10.1371/journal.pgen.1005428)
Supplement: S1 Text — (DOCX) [file pgen.1005428.s008.docx]

**Text S1. Inspection of the RNA-seq data set to ensure appropriate data quality for studying gene expression variability.**

***Checking for appropriate normalization***

We downloaded the RNA-seq data from the journal website, where the data had already been pre-processed, including normalized by the authors. For stability, a log_2_(*x* + 1) transformation was applied to the data where *x* represents the RPKM-normalized expression. The boxplots below display the transcriptome-wide gene expression distributions for all cell samples included (each box corresponds to a cell from a single embryo). A total of 20, 286 genes were present in the data set. We see generally acceptable consistency in the gene expression distributions across all cell samples, and therefore conclude normalization was applied appropriately.

***Applying an expression-based filter.***

Yan et al. used a cut-off of RPKM ≥ 0.1 to identify genes that were expressed in a single cell. To study gene expression variability, it is important that genes are detectable above a certain level of expression, and that this minimum is achieved in a considerable proportion of the cell population profiled. Otherwise, the variability observed in gene expression could be a result of failure to detect transcripts of a gene across cells, and may reflect a technical issue rather than a biological one. We applied the same threshold, and calculated for each gene the percentage of the total cell population that it was expressed in. The following histogram displays how many genes were expressed at different percentages of the total cell population.

For example, there were 2124 genes expressed in all 124 cells profiled. All other genes in the data set were expressed in less than 100% of the cell population. We decided to retain only those genes that satisfied the expression criterion for at least 75% of the total cell samples (i.e. a gene had to be expressed in 93 cells or more of the total 124 cells profiled). This resulted in the retention of 8105 genes.

***Evaluating consistency of the embryos: are they adequate replicates for estimating inter-cellular variability?***

To measure the inter-cellular expression variability of each gene *g*, we adopted the following statistic *SDC_g_*:

$${SDC}_{g}=\frac{1}{E}\sum_{j=1}^{E} \sqrt{\frac{1}{N_{j}}\sum_{i=1}^{N_{j}} \left( x_{ij}-\bar{x}_{j} \right)^{2}}$$

where $x_{ij}$ is the expression level of gene *g* in the *i*-th cell of the *j*-the embryo for a total number of *E* embryos. For each embryo *j*, there are a total of *N_j_* cells that have been profiled. $\bar{x}_{j}$ represents average expression occurring in the *j*-th embryo. The *SDC* captures the standard deviation (SD) of expression levels observed between cells belonging to the same embryo, that is then averaged across all embryos to give an overall measure of inter-cellular variability. Inherent in the SDC formula is the assumption that the embryos represent replicates to estimate the inter-cellular variability, and therefore genes showing consistent inter-cellular variability between embryos are given higher weight by the *SDC*.

As a means to evaluate the consistency of the inter-cellular variability between embryos, we also computed the *SDE_g_*:

${SDE}_{g}=\sqrt{\frac{1}{E}\sum_{j=1}^{E} \left[ \frac{1}{N_{j}}\sum_{i=1}^{N_{j}} \left( x_{ij}-\bar{x}_{j} \right)^{2}-\frac{1}{E}\sum_{j=1}^{E} \frac{1}{N_{j}}\sum_{i=1}^{N_{j}} \left( x_{ij}-\bar{x}_{j} \right)^{2} \right]^{2}}$.

which is simply the SD of the embryo-specific SDC measures. We defined the SDE, a statistic that measures how consistent the inter-cellular expression variability (SDC) of a gene is between embryos. Genes that have more variable inter-cellular expression may be involved in embryo-specific processes and would be of interest in understanding human diversity (for a larger number of embryos). The violin plots below show the distribution of SDE (panel a) and SDC (panel b) measures for all genes retained by the filtering step. The global distribution of inter-embryo variability remained consistent throughout the four stages, suggesting that the transcriptomes of the embryos are relatively stable compared to each other. This observation lends support to our assumption to treat the embryos as replicates to estimate the inter-cellular expression variability in our study.

***Inter-cellular expression variability is independent of cell number sampled per stage.***

As with any population statistic, a relationship exists between our ability to estimate variability and the number of samples collected. In the context of this study, most stages featured a different number of cells that were profiled, a design that reflects both biological factors and technical capabilities. To investigate the degree of dependency between cell number and variability, we estimated the inter-cellular expression variability using a fixed number of four cells sampled from the total number of cells available, and these calculations were done for all possible 4-cell combinations (outlined in the schematic below).


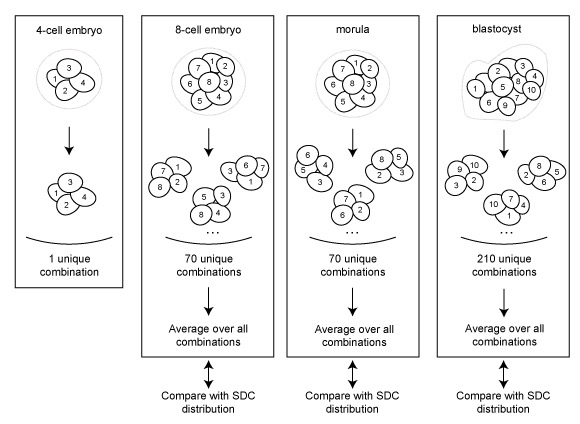


The boxplots show that comparisons between variability estimates based on the 4-cell combinations and total cells available displayed negligible difference, and we were able to see the same trends recapitulated across the four stages. Red boxes reflect the inter-cellular variability obtained with the available number of cells, e.g. estimates for morula were based on ten cells. Therefore we concluded that the number of cells was not driving the differences observed in variability across developmental stage.


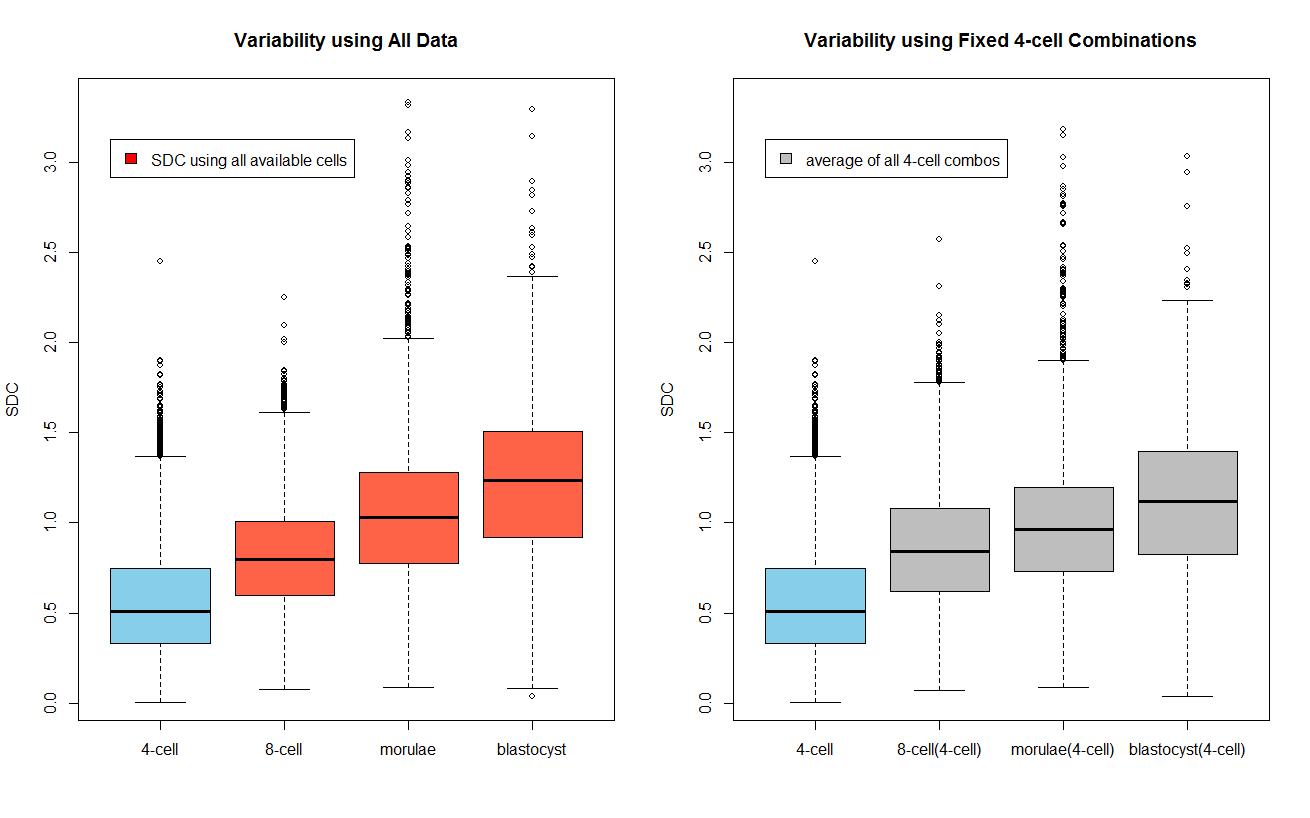


*Assessing the preservation of specific genes when the number of embryos are reduced.*

One way to assess the stability of our results is to determine the amount of overlap in the specific genes obtained with original results when the data is halved or reduced. Since the intention of calculating the intercellular expression variability is to assess variability within an embryo, we wanted to ensure that data reduction was done in a way where the cells from an embryo were retained. Therefore, we dropped one embryo from each stage available, i.e. for 4-cell we used the data from only two out of the three embryos.


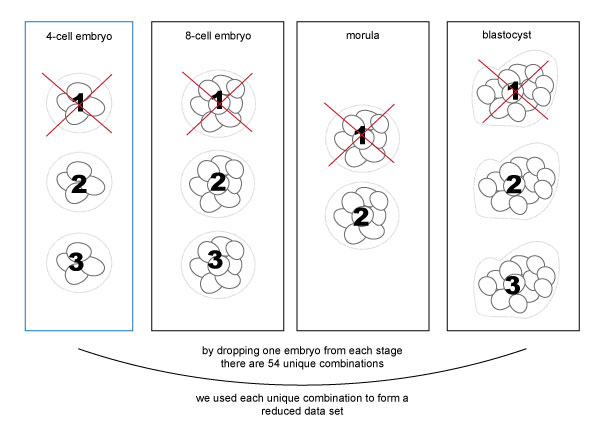


When we drop one of the embryos from each stage, there are 54 unique combinations for forming this reduced data set. For each permutation now, we have two embryos for the 4-cell stage, two embryos for the 8-cell stage , one embryo for the morula stage, and two embryos for the blastocyst stage.

For each reduced data set, we applied Levene’s test to determine which genes had a significant change in expression variability across stage. For genes with a non-significant P-value (Benjamini-Hochberg adjusted P-value > 0.05), we clustered their SDC values based on a Normal mixture model into three clusters representing low, medium and high levels of expression variability. Stable genes were designated as those belonging to the cluster with the lowest level of average expression per stage (in all permutations, there was one cluster that had the lowest level of variability for at least three out of four stages). We compared the overlap in stable and variable genes with the original lists that were determined on the full set of data.


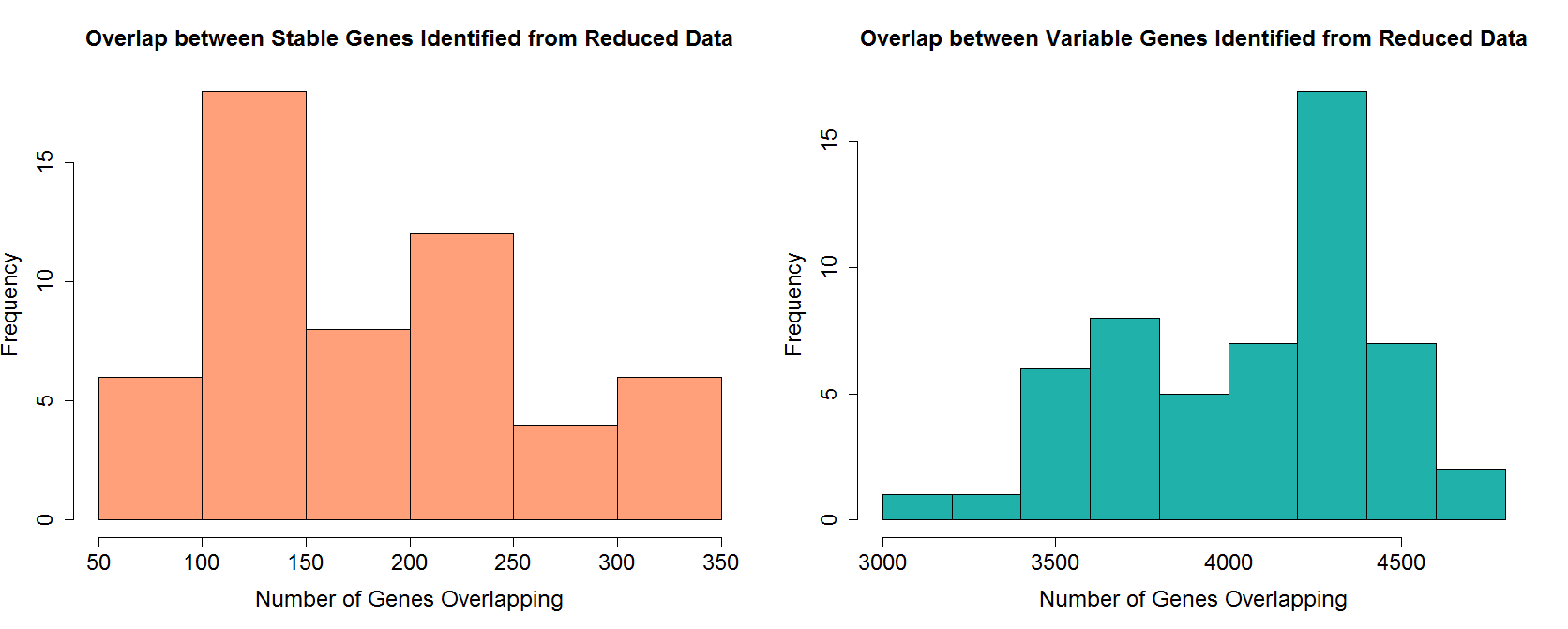


Although there were no genes that were present consistently in all overlaps between the stable gene lists computed for the 54 reduced data sets, when we calculate the union of all stable gene lists , we find that the 955 stable genes are all represented. The same result was obtained for the variable genes. This suggests that if we take all the gene lists from all 54 reduced data sets we will be able to recover the original stable and variable gene lists that were identified from the full data set.

These results show that there is some consistency observed between the identity of genes that are stable or variable, however it is important to keep in mind that with only one or two embryos per stage, it will still be more challenging to estimate gene expression variability than having access to three embryos.
