# Supplementary material for: Variability of Gene Expression Identifies Transcriptional Regulators of Early Human Embryonic Development
Source: PLoS Genet. 2015 Aug 19;11(8):e1005428. doi: 10.1371/journal.pgen.1005428 (PMC4546122; doi:10.1371/journal.pgen.1005428)
Supplement: S2 Text — (DOCX) [file pgen.1005428.s009.docx]

**Text S2. Investigating the impact of potential aneuploidy on inter-cellular expression variability**

Pre-implantation embryos are known to exhibit mosaicism in the chromosomal content of their cells. Detection of aneuploidy would require at a minimum, the availability of genome sequence which was not generated in the original publication. While we are unable to definitely diagnose which cells were aneuploid, we have analyzed the RNA-seq data to investigate whether the potential impact of aneuploidy affected our results on intercellular expression variability.

We used expression data from all 7288 autosomal genes that had an Entrez Gene ID annotation and passed the expression detection threshold (RPKM ≥ 0.1). To obtain a surrogate estimate of a chromosome’s expression, we averaged all genes located on the same chromosome, and standardized this value by the total average of all 7288 autosomal genes.

The underlying assumption is that cells with a full set of chromosomes should have each chromosome expressed at a normalized ratio of 1, in the absence of technical noise. We found that for morula stage, one of these chromosomes may be monosomy 10, in embryo 1. We have shown that the expression variability in the morula group on chromosome 10 is not extreme and likely does not affect our results.

The plots below depict the normalized expression ratios across chromosomes of each cell for each embryo sample. The orange, yellow and green dots represent a cell for embryos 1,2, and 3 respectively. Overall, most cells fall within a normal range of expression for all stages. We noticed for morula, a few cells deviated from the ratio = 1 baseline. This may reflect previous reports that point to the cleavage stage where mosaicism is most prevalent. We followed up on genes located on chromosome 1 in cell 1 of embryo 1 at the morula stage to investigate whether this potential aneuploidy impacted on our results.

**
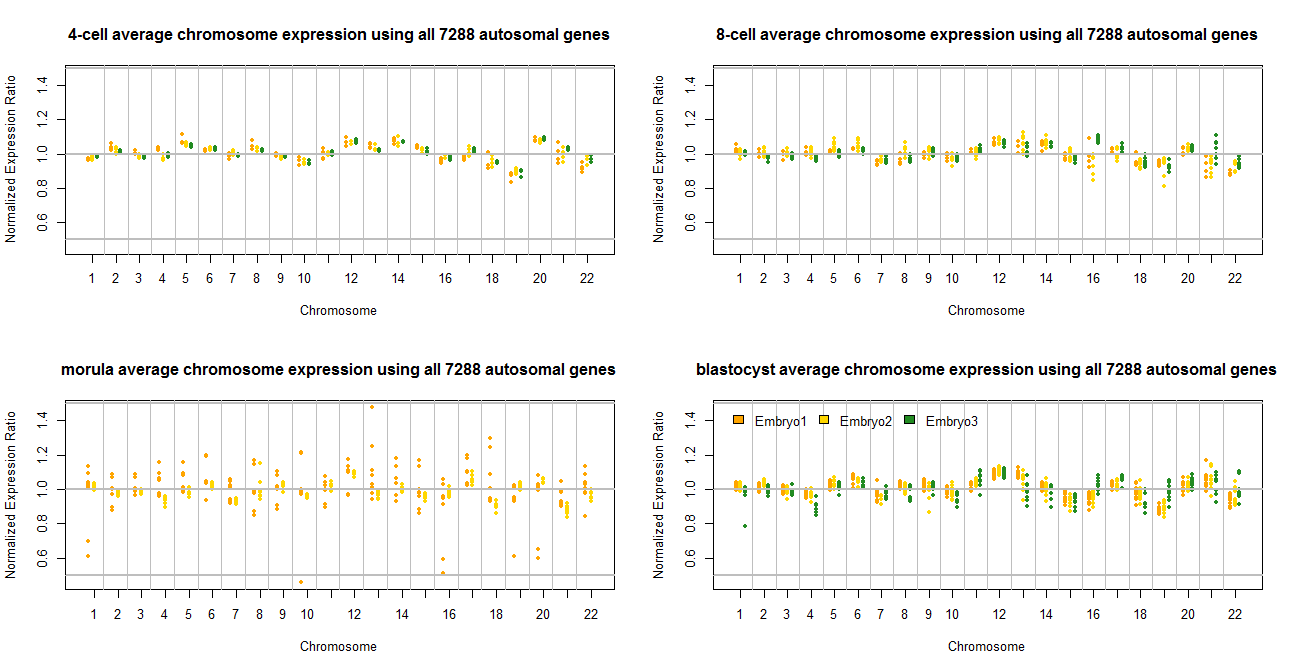
**

In the following boxplots, we showed that the distribution of inter-cellular expression variability is relatively consistent across all chromosomes in all stages. This suggested that even though chromosome 10 in cell 1 of embryo 1 may have lower overall expression compared to all other chromosomes, this difference was not extreme enough to affect the variability measures calculated for genes on chromosome 10.

**
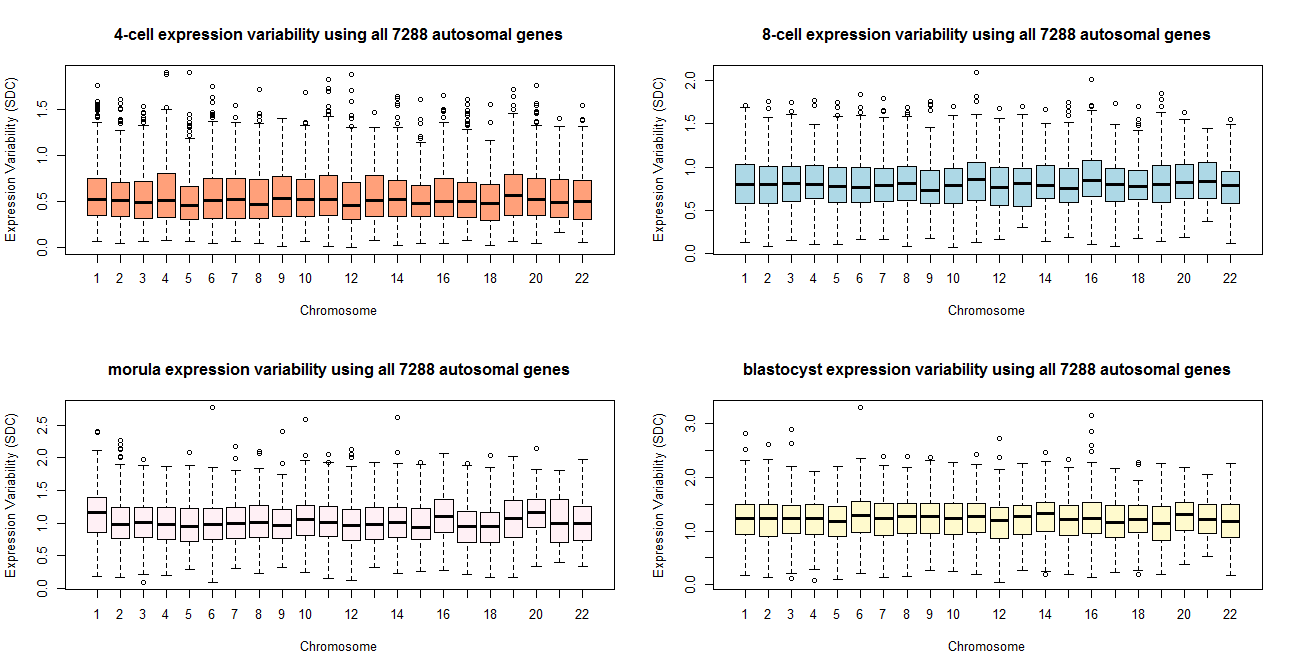
**

We also looked at the resulting effect of removing data for genes on chromosome 1 from cell 1 in embryo 1 on the inter-cellular variability. In the plots below, we showed that the overall distribution of autosomal genes in the morula stage was unaffected by the absence or inclusion of these 296 genes on chromosome 1 (the average SDC shifted by 0.4%). Focusing on the 296 genes only, the distribution also varied slightly (11% shift in average SDC), with the overall coverage of SDC measures being the same.

**
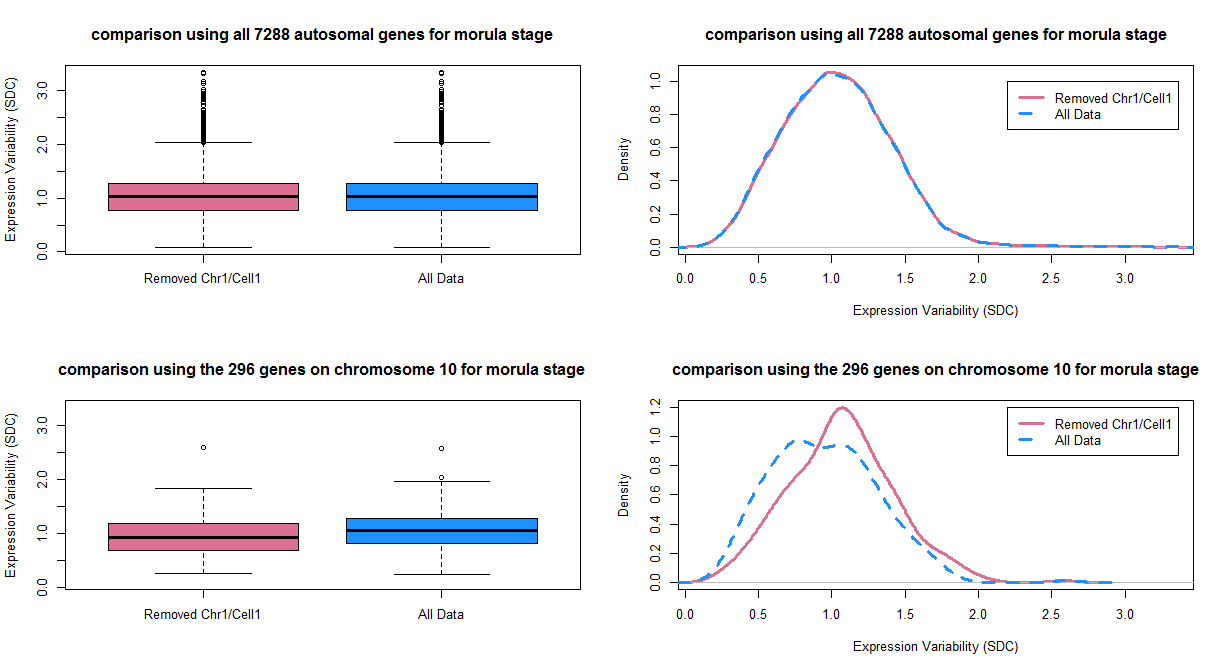
**
