# Supplementary material for: Variability of Gene Expression Identifies Transcriptional Regulators of Early Human Embryonic Development
Source: PLoS Genet. 2015 Aug 19;11(8):e1005428. doi: 10.1371/journal.pgen.1005428 (PMC4546122; doi:10.1371/journal.pgen.1005428)

**Text S3. Characterizing the statistic to measure gene expression variability.**

Variability is defined as the spread or dispersion in data. Just like any other statistical property, there are several different estimators to choose from. For the purpose of studying gene expression variability, one property of a good estimator that we look for is the lack of correlation with the average expression of a gene. This is because a correlation between the level of average and variable expression makes it difficult to study patterns of expression variability, since these will recapitulate the trends seen in average expression. Two candidate statistics for modeling gene expression variability are the standard deviation (SDC) and the coefficient of variation (CV). Both of these statistics have been used to study variability in biology, and each come with their own set of advantages and disadvantages. We summarize these briefly in the table below.

**Table 1.** Summary of the advantages and disadvantages associated with the Standard Deviation and Coefficient of Variation for studying expression variability.

|  | *Advantage* | *Disadvantage* |
| --- | --- | --- |
| **Standard Deviation** | - Same units as the average expression so this statistic has an easy interpretation. | - On its own, does not account for changes in average expression. It is necessary to specify levels of average as well as standard deviation for specific patterns, or investigate the nature of its correlation with average expression. |
| **Coefficient of Variation** | - Measure of variability that is standardized by average expression. | - Subject to inflation for genes with average expression close to zero. - It is difficult to interpret the behavior of a gene based on its level of average expression and expression variability. These two measures are combined into a single ratio. |

For expression variability, there is often an assumption that variability and average expression will be negatively correlated. The motivation behind this assumption is that genes with lower levels of average expression are more difficult to detect reliably and hence their expression levels will fluctuate and vary more. Genes that have higher levels of expression on the other hand are easier to obtain reliable estimates for and hence will be more consistent. Alternatively, an assumption in the opposite direction, that there is a potential positive correlation between these two statistic is also commonly held. Clearly this is an area that needs to be investigated.

*Investigating the potential correlation between mean and variability of gene expression.*

For this data set, we decided to investigate the nature of the correlation between average expression and expression variability as represented by the SDC and CV. For all 8105 genes in the data set, we plotted the average expression against the variability statistic, and calculated the correlation using the Pearson correlation coefficient for each developmental stage. We note that the CV has the largest absolute correlation for all four stages. From the scatter plots it is also apparent that the CV had the most consistent correlation, in that across the spectrum of low to high average expression, the CV went from high to low levels of variability. The SDC in contrast had variability levels that peaked for middle-range average expression and lower levels of variability for low and high average expression.

**Table 2.** Correlation coefficient between average expression and expression variability for all genes in the Yan human embryo data set.

| ***Pearson Correlation Coefficient*** | ***4-cell*** | ***8-cell*** | ***Morula*** | ***Blastocyst*** |
| --- | --- | --- | --- | --- |
| **SDC** | -0.357 | -0.187 | 0.120 | -0.225 |
| **CV** | -0.744 | -0.760 | -0.750 | -0.840 |

Based on these results, we decided to use the SDC for our study of expression variability since this is the least correlated variable. Where necessary, we have also put criteria based on average expression, e.g. in deriving the stage-specific variability markers so as to not ignore the potential correlation between these two variables.

**Figure 1.** Scatter plot of the standard deviation versus average expression for all 8105 genes in the Yan human embryo data set for each developmental stage. The solid line represents the **lowess** curve that shows the trend through the data.


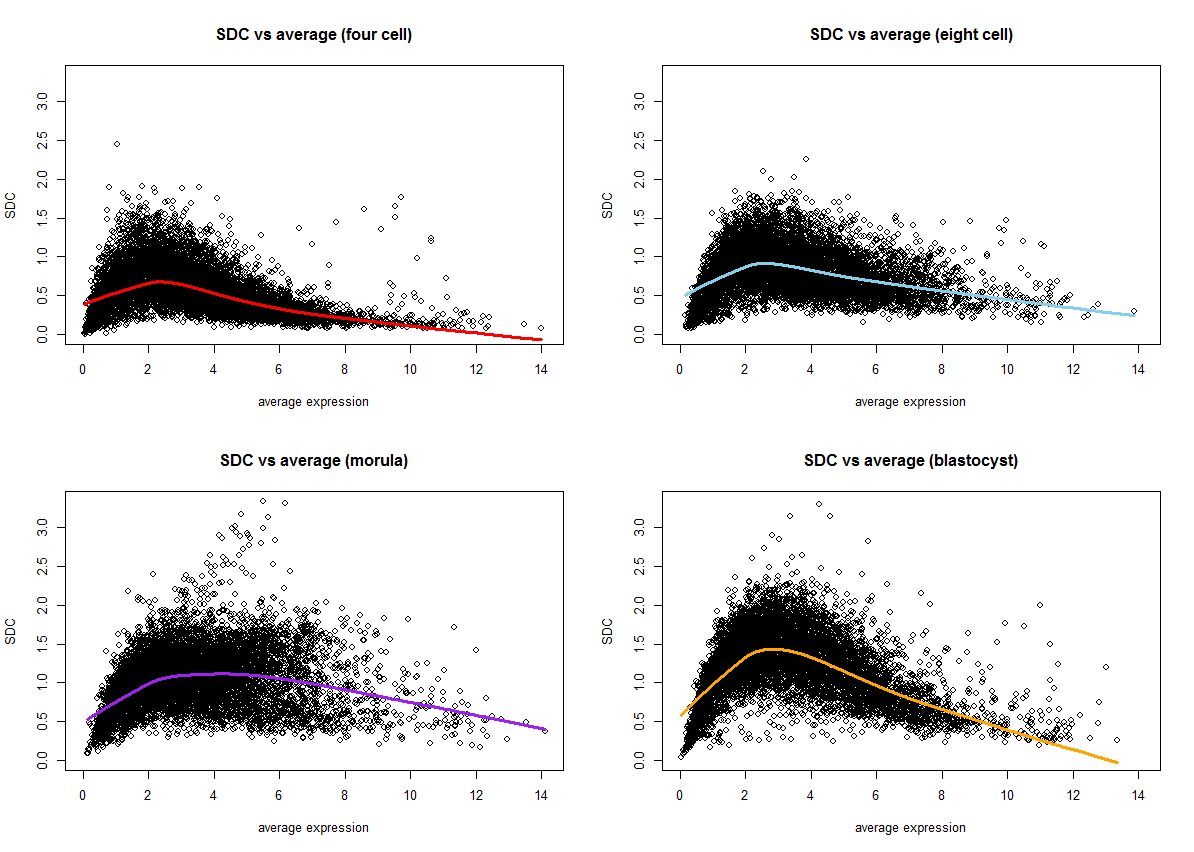


**Figure 2.** Scatter plot of the coefficient of variation versus average expression for all 8105 genes in the Yan human embryo data set for each developmental stage. The solid line is the **lowess** curve that shows the trend through the data.


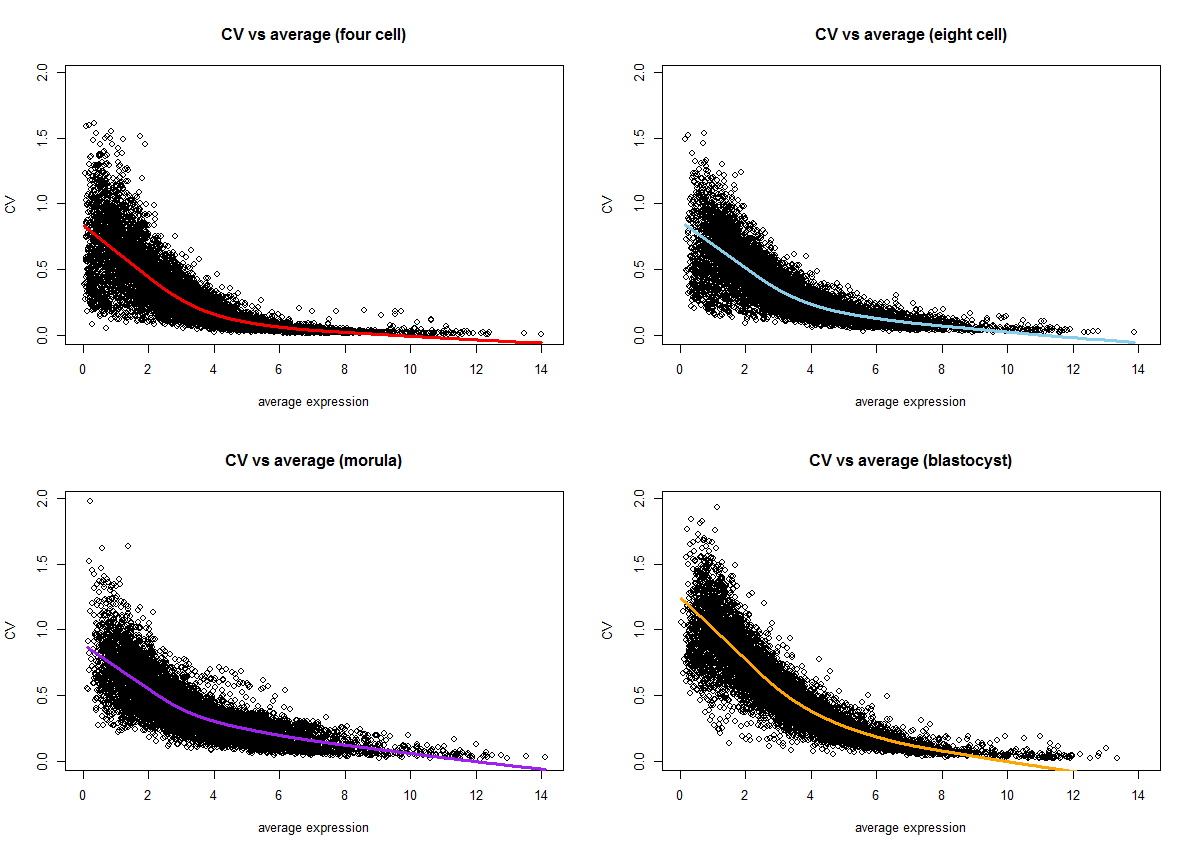


*Simulation to test the performance of the SD and the CV in distinguishing different levels of expression variability.*

A simulation was conducted in order to test the performance of the SD and CV statistics in uncovering different levels of variability in data. To best mimic a real data scenario, we generated expression data for 1000 genes where **250** genes had data simulated under N(*μ_1_*, *σ_1_^2^*), **500** genes under N(*μ_2_*, *σ_2_^2^*), and **250** genes under N(*μ_3_*, *σ_3_^2^*). Each test case highlighted a different special feature where one statistic, either the mean, SD or CV was kept constant (Table 3). The goal was to determine under which test cases, the SD or CV was able to identify the genes with different levels of variability.

**Table 3.** Design parameters and special features for the three test cases that data was simulated under.

| 1000 Genes, 100 Simulations | **Test Case 1** | **Test Case 2** | **Test Case 3** |
| --- | --- | --- | --- |
| **Mean** | *μ_1_* = 1, *μ_2_* = 2, *μ_3_* = 3 | *μ_1_* = 1, *μ_2_* = 2, *μ_3_* = 3 | *μ_1_* = 2, *μ_2_* = 2, *μ_3_* = 2 |
| **Standard Deviation** | *σ_1_* = 2, *σ_2_* = 4, *σ_3_* = 6 | *σ_1_* = 3, *σ_2_* = 3, *σ_3_* = 3 | *σ_1_* = 1, *σ_2_* = 2, *σ_3_* = 3 |
| **Coefficient of Variation (σ/μ)** | *cv_1_* = 2, *cv_2_* = 2, *cv_3_* = 2 | *cv_1_* = 3, *cv_2_* = 1.5, *cv_3_* = 1 | *cv_1_* = 0.5, *cv_2_* = 1, *cv_3_* = 1.5 |
| **Special Feature** | Same **CV**, Different **SD** | Different **CV**, Same **SD** | Different **CV**, Different **SD** |

We used a mixture model algorithm implemented in the **mclust** package to estimate the most appropriate number of clusters in the simulated data. When this estimate is calculated by the mixture model, an assumption can be made about the variability of the Normal mixtures that are fitted, i.e. they can be assumed to have different variance parameters or they can be assumed to have the same variance. We tested the number of clusters ranging from two to five, and under both assumptions of different variance parameters (denoted by a V in the figures below), and the same variance parameter (denoted by an E).

Because we simulated the data, by design, the correct answer for the algorithm is **three clusters with different variance parameters, i.e. 3-V for Test Cases 1 and 3**, and **3-E for Test Case 2**. For a given number of clusters, the mixture model will assign a score (the Bayesian Information Criterion, BIC) that reflects how likely this specific model fits the data. In Fig 3., we show the BIC scores assigned by the different variability statistics, and the correct answers for each test case were indicated by the orange dotted line. The model (i.e. the number of clusters) chosen by the **mclust** algorithm with the highest median BIC scores was colored red.

We can see from Fig. 3 that in Test Cases 1 and 3, using the SD and CV, these models were able to assign high BIC scores and both statistics identified the correct result. For Test Case 1, although the CV identified 3-V as the correct model, the BIC scores were very similar for models 2-V, 3-V, 4-V and 5-V calling into question the specificity of this statistic’s ability to distinguish between the different models. This is understandable given that under Test Case 1, the data was simulated such that the CV was the same across the three groups of genes. For Test Case 2, the data was simulated so that the SD would be the same for all three groups of genes but it is interesting to note that both statistics failed to obtain the correct answer. This suggests that neither CV or SD are able to identify the correct model.

Mixture models are also able to estimate which gene belongs to which cluster. Therefore, we can look at the percentage of genes that are misclassified to each of the three groups by the mixture model based as another way to gauge performance of the CV and the SD. We calculated the percentage of misclassified genes in each group defined by the three different parameters **(*μ_1_*, *σ_1_*) for Group 1, (*μ_2_*, *σ_2_*) for Group 2, (*μ_3_*, *σ_3_*) for Group 3**.

In Fig. 4., we see that for all three test cases, the CV has the higher rates of misclassification. For Test Cases 1 and 3, the SD has almost 100% perfect classification rates. The CV performed the best for Test Case 3, however even these rates are higher than those observed for with the SD. While we know from Fig. 3 that both CV and SD fail to find the correct model for Test Case 2, the misclassification rates are quite different for the two statistics. For the SD, the misclassification rate is approximately 60-65% for all three groups, whereas for the CV, we see nearly a 100% misclassification rate for 50% of the genes (Group 1 and Group 3), and nearly 0% misclassification for the remaining 50% of genes in Group 2.

**Figure 3.** Comparing the performance of the SD versus the CV in determining the correct number of clusters. The correct answer is indicated by orange dotted line. The model with the highest median Bayesian Information Criterion score assigned is highlighted in red.


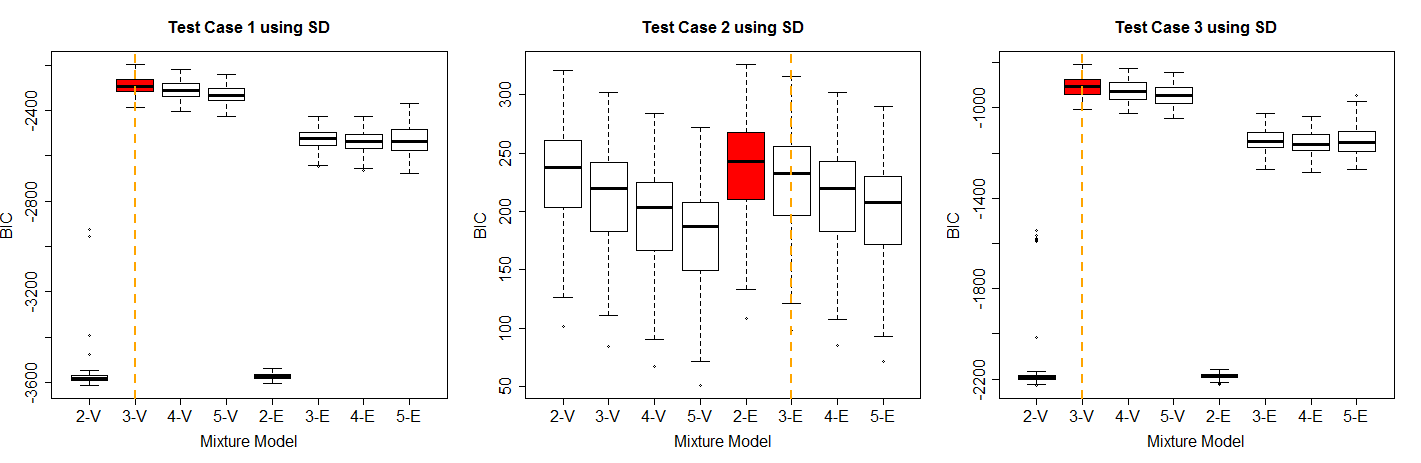

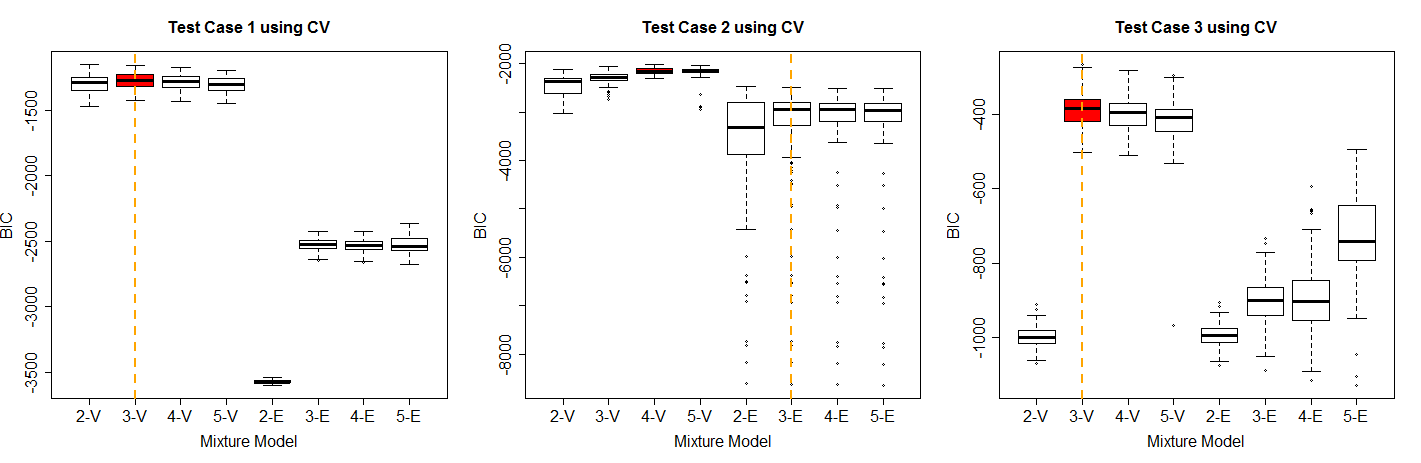


**Figure 4.** Comparing the performance of SD versus CV based on misclassification rate of genes to their specific groups.


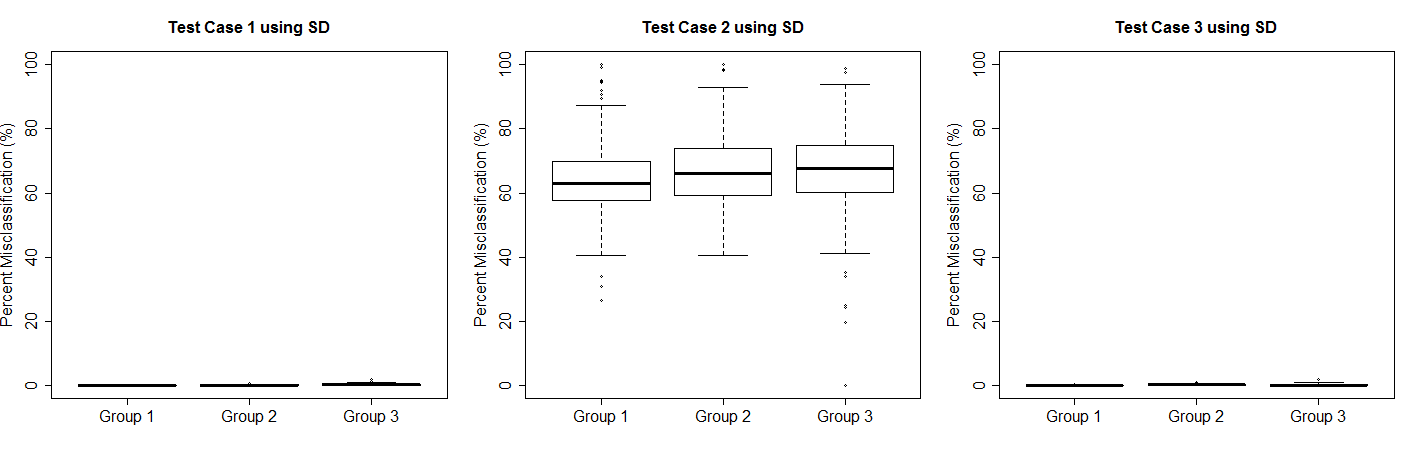


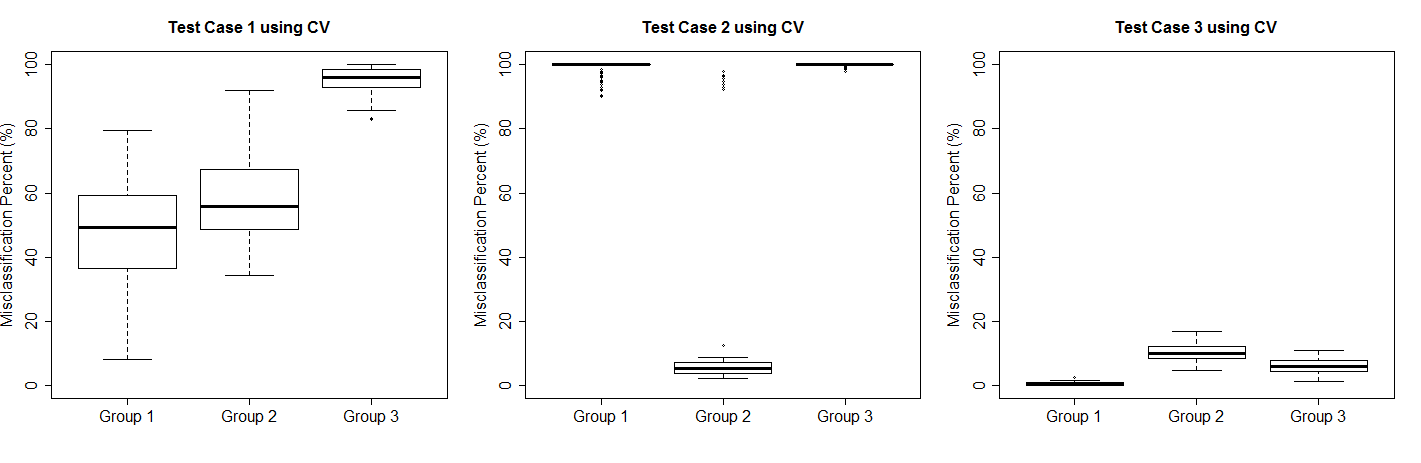


*Conclusions from the simulation study.*

Overall, based on the test cases presented, the SD demonstrated slightly better performance than the CV for this simulation study. Both statistics failed at Test Case 2 (when the SD was the same), and both succeeded in finding the correct answer for Test Case 3. However for Test Case 1, the SD was able to distinguish the correct answer from the other candidates more definitively, whereas the CV had similar scores for the many different models. Based on misclassification rates, both CV and SD performed poorly for Test Case 2. The SD had for Test Cases 1 and 3, nearly very low misclassification rates, and hence overall was the better performer compared to the CV. It is worthwhile highlighting that there are more assumptions inherent in this simulation study, that in order to conduct a more comprehensive analysis, would warrant further investigation.

*Comparing the main results obtained from our expression variability analysis with those obtained with CV on the human embryo data set.*

In order to investigate the impact of the statistic used to study expression variability, we repeated our analyses using the CV to see whether we would obtain similar results to when the SDC statistic was used.

We started by applying the mixture model to cluster genes into their variability states. Using **mclust**, we identified eight clusters for the 4-cell stage, five clusters for the 8-cell, morula and blastocyst stages. As a comparison, we also repeated this step using the average expression levels to see how many distinct gene clusters were present in the data (Table 4).

Based on average expression, the mixture model identified six clusters for the 4-cell stage, five clusters for the 8-cell stage, and six clusters for the morula and blastocyst stages. We note that while the SDC, CV and average expression all generate a different set of clusters, **the average and CV are more similar in the number of clusters identified for each stage**.

**Table 4.** Number of gene clusters identified for each stage that represent different levels of expression variability.

| Number of Mixtures | 4-cell | 8-cell | Morula | Blastocyst |
| --- | --- | --- | --- | --- |
| SDC | 4 (V) | 3 (E) | 3 (V) | 2 (V) |
| CV | 8 (V) | 5 (V) | 5 (V) | 5 (V) |
| Average | 6 (V) | 5 (V) | 6 (V) | 6 (V) |

We also inspected the shapes of the density distributions of the expression variability, and how these changed during embryonic development (Fig. 5). We noticed that the average expression and CV distributions shared similar characteristics in that we saw a lot of overlap between all four stages.

**Figure 5.** Density distributions across all four stages for each population summary statistic.


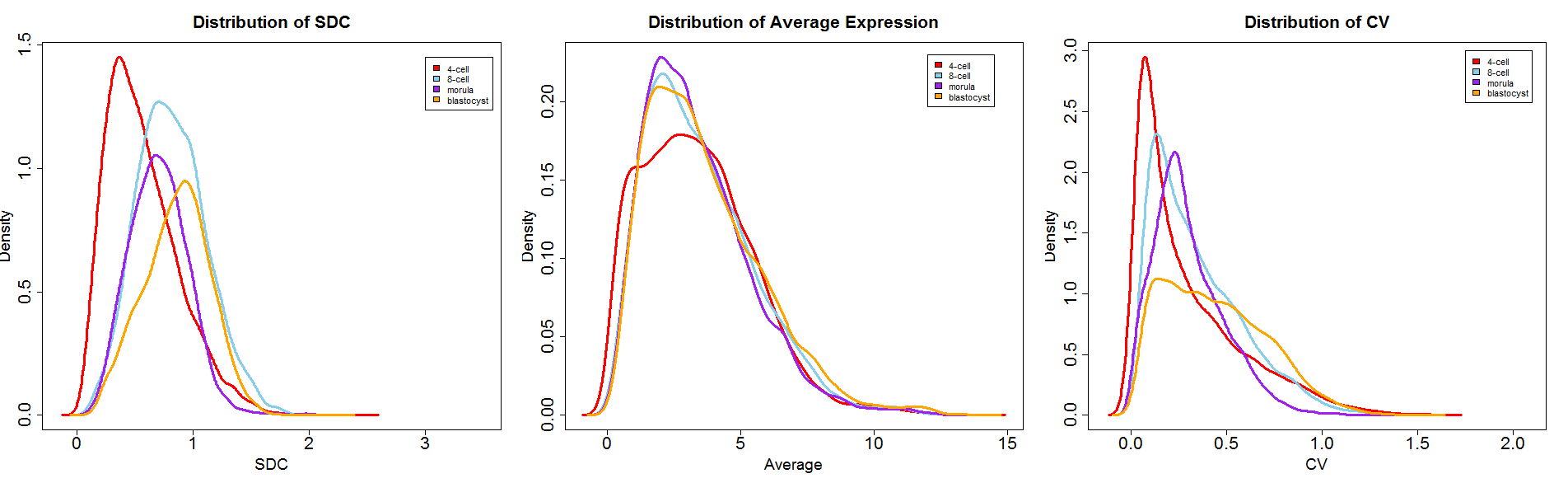


*Comparing stable gene list membership between SDC and CV as the expression variability statistic.*

We also compared the overlap of genes that were identified as stably expressed using both the SDC statistic and the CV.

**Figure 6.** Overlap of genes that were classified as stably expressed based on CV and SDC.


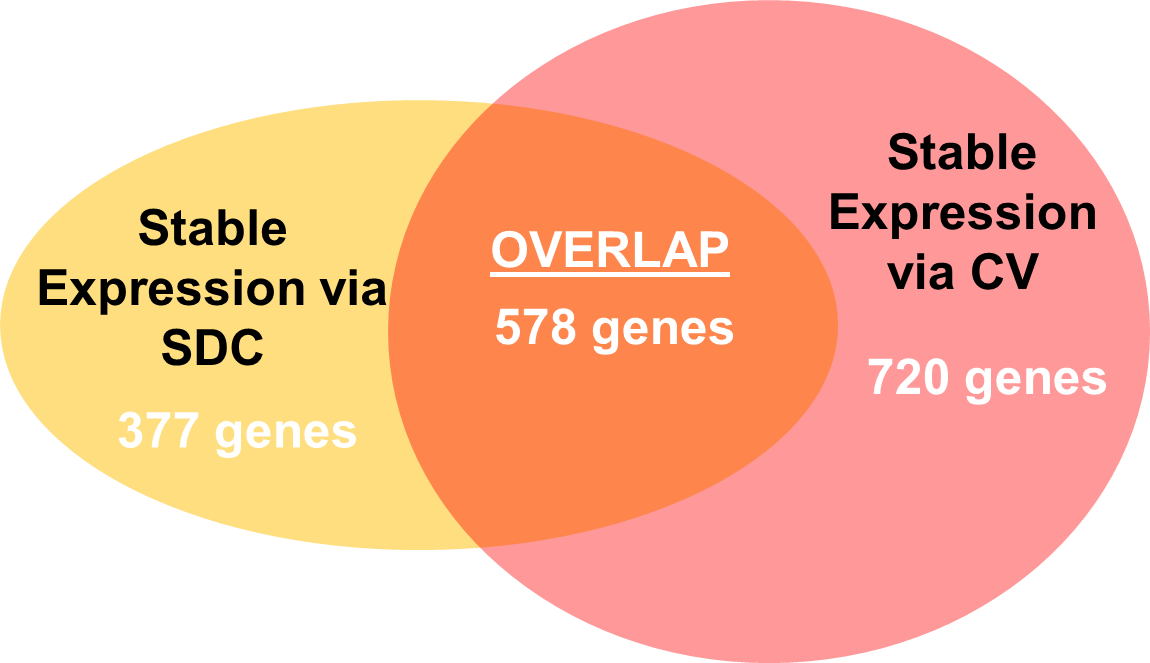


*Detecting stage-specific variability markers using SDC and CV*

Stage-specific variability markers were detected based on a gene satisfying the following criteria:

1. A statistically significant change in variability as assessed by Levene’s test (adjusted P-value < 0.05).
2. The lowest expression variability compared to all other stages (using the SDC).
3. The highest average expression level compared to all other stages.

We computed the CV for the stage-specific variability markers that were identified to see whether these genes also had low CV values, and whether we would have detected these genes based on the CV rather than the SDC.

In Fig. 7, we see that for morula and blastocyst stages, all the variability markers had CV values that fell below the first quartile. For the 8-cell stage, there were five marker genes that had CV values above the first quantile and less than the median (NT5C3, PANK2, PTPN9, RPS6KA5, UCK1). From this figure we conclude that the majority of marker genes had low CV values.

**Figure 7.** The distribution of CV values for genes, ranked from lowest to highest. The red dots indicate genes that were designated variability markers in our previous analysis.


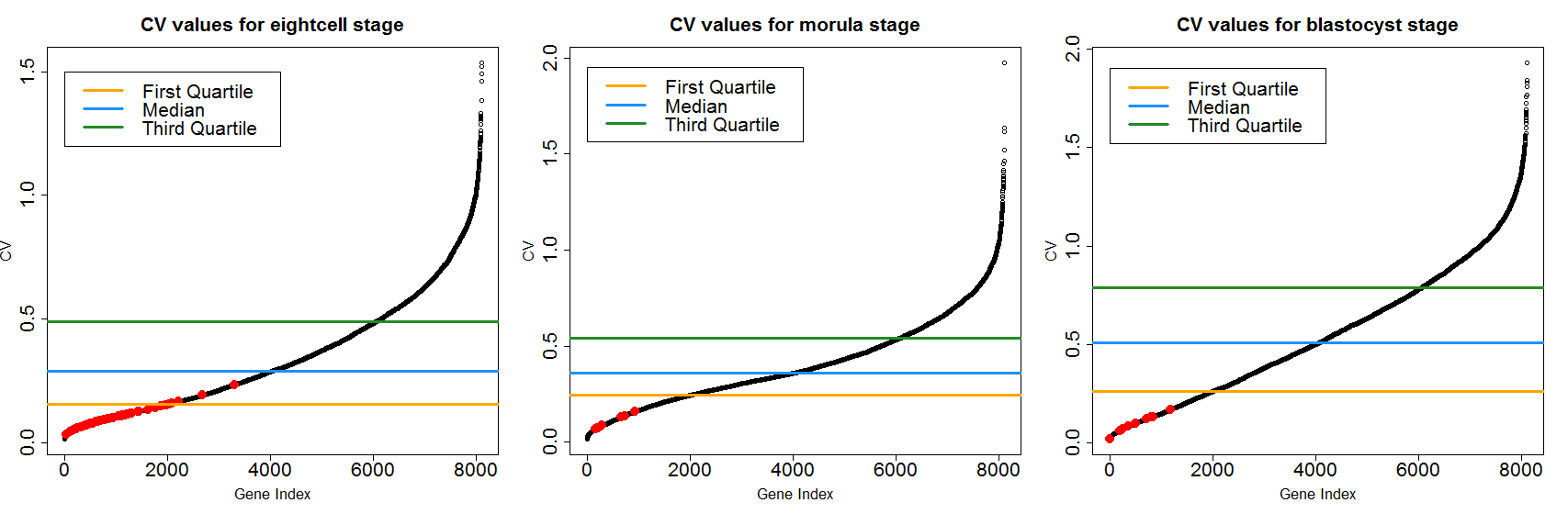


While it is fair to say that genes with low SDC generally had low CV values, is the converse also true, that genes with low CV also have low SDC? We extracted genes with a CV value less than the first quartile for a specific stage and inspected their corresponding average expression and SDC values. In Fig. 8, we see that there is considerable spread in the range of SDC values adopted by these low CV genes. In fact, for all genes with a CV less than the first quartile, the percentage of these genes that also have a SDC less than the first quartile was 15.5% for the 8-cell stage, 50.4% for the morula stage, and 61.5% for the blastocyst stage. Clearly from these results there is some overlap in what the SDC and CV are detecting, but also some considerable differences too.

**Figure 8.** The distribution of average versus SDC for genes with a CV falling below the first quartile. We can see that genes with low CV nevertheless can have high SDC.


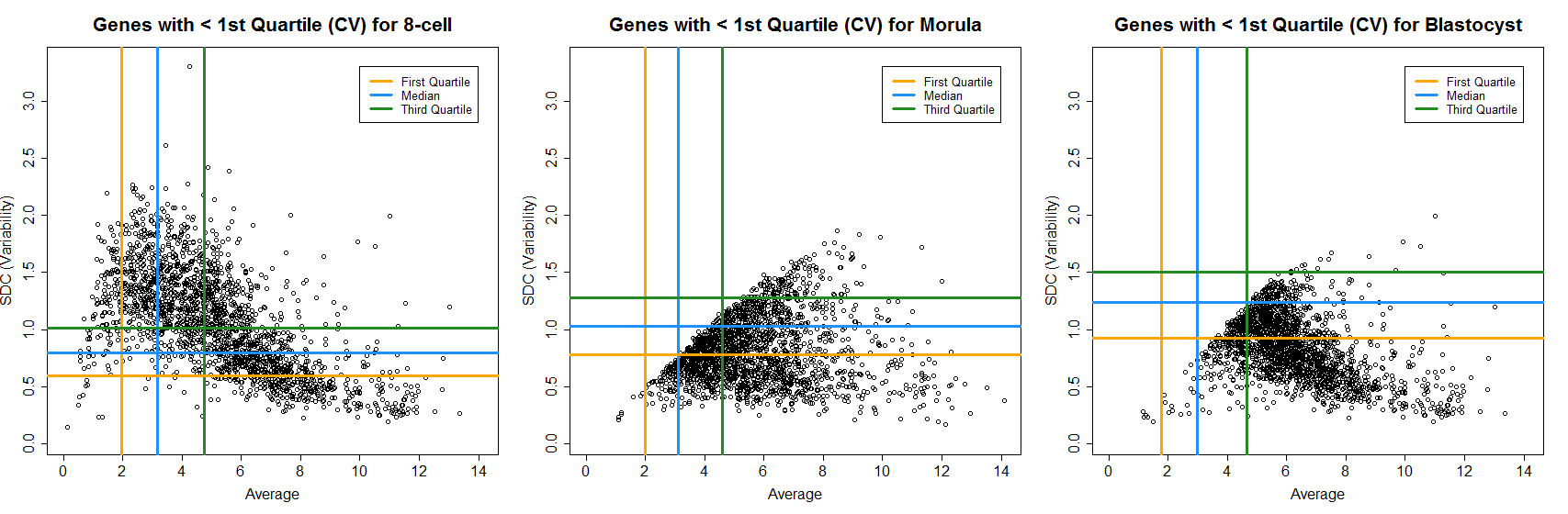

Supplement: S3 Text — (DOCX) [file pgen.1005428.s010.docx]
