# Supplementary material for: Variability of Gene Expression Identifies Transcriptional Regulators of Early Human Embryonic Development
Source: PLoS Genet. 2015 Aug 19;11(8):e1005428. doi: 10.1371/journal.pgen.1005428 (PMC4546122; doi:10.1371/journal.pgen.1005428)
Supplement: S4 Text — (DOCX) [file pgen.1005428.s011.docx]

**Text S4. Distinguishing the contributions of the maternal and zygotic transcriptome to the developing embryo.**

In addition to the four development stages that were selected for our study, 4-cell, 8-cell, morula and blastocyst, Yan et al. [1] also profiled the transcriptomes of the oocyte from three different human embryos. We therefore have the opportunity to infer which genes with stable, invariant expression at the early stage of development were contributed to the zygote from the maternal transcriptome.

*Identification of genes that were likely to be expressed in the maternal transcriptome.*

Yan et al. had also collected the single cell transcriptomes of 8 human embryonic stem cells (hESCs) at passage 0, and 26 hESCs at passage ten. We reasoned that a gene was more likely to be expressed in the maternal transcriptome if it was highly expressed in the oocyte (greater than the global median of all gene expression in the oocyte) and lowly expressed in the hESCs (less than the global median of all gene expression in both p0 and p10 hESCs). The additional requirement that genes be down-regulated in hESCs was motivated by the possibility that highly-expressed genes in the oocyte may simply represent housekeeping genes that are ubiquitously and highly expressed in most tissues.

**Figure 1.** Density of expression for the oocyte and the hESCs from the Yan human embryo data set.


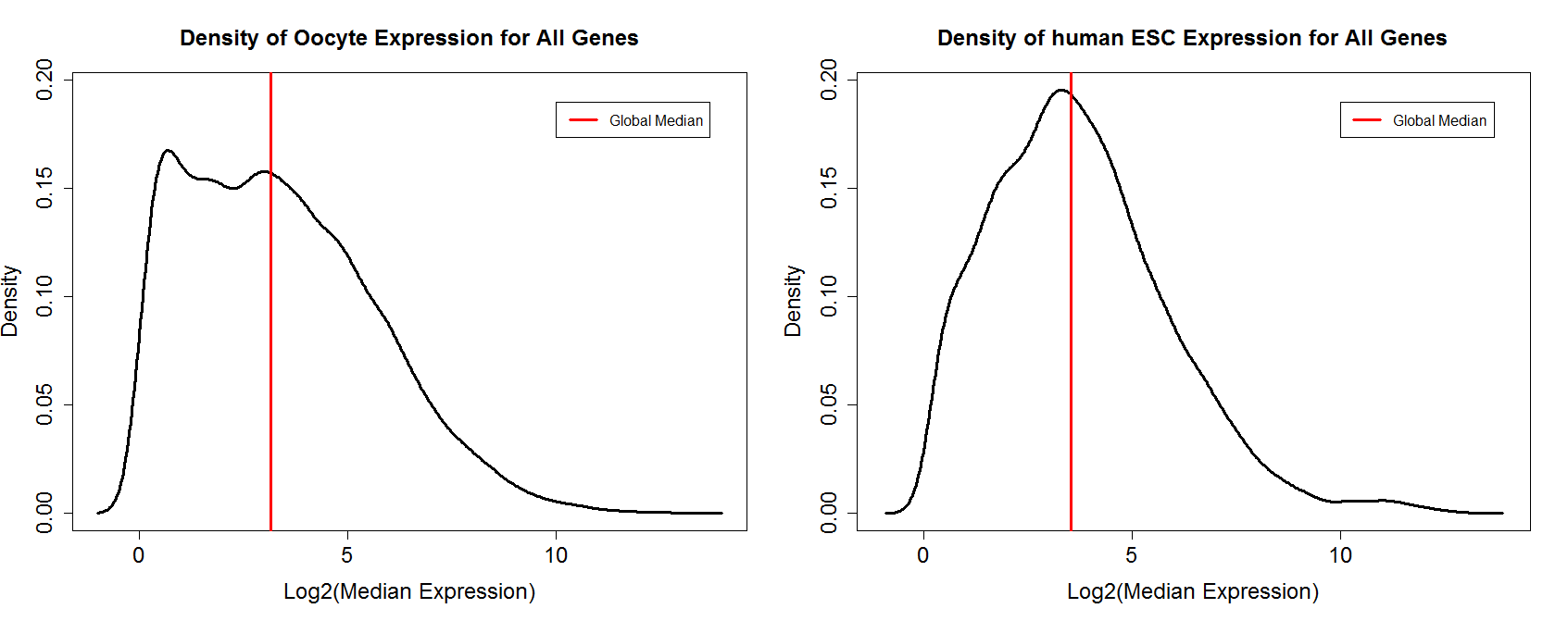


Based on this criteria, we identified 1414 genes (out of a total of 8105 genes) that were highly-expressed in the maternal transcriptome.

**Figure 2.** Distribution of genes that are highly expressed in oocytes but lowly expressed in hESCs.


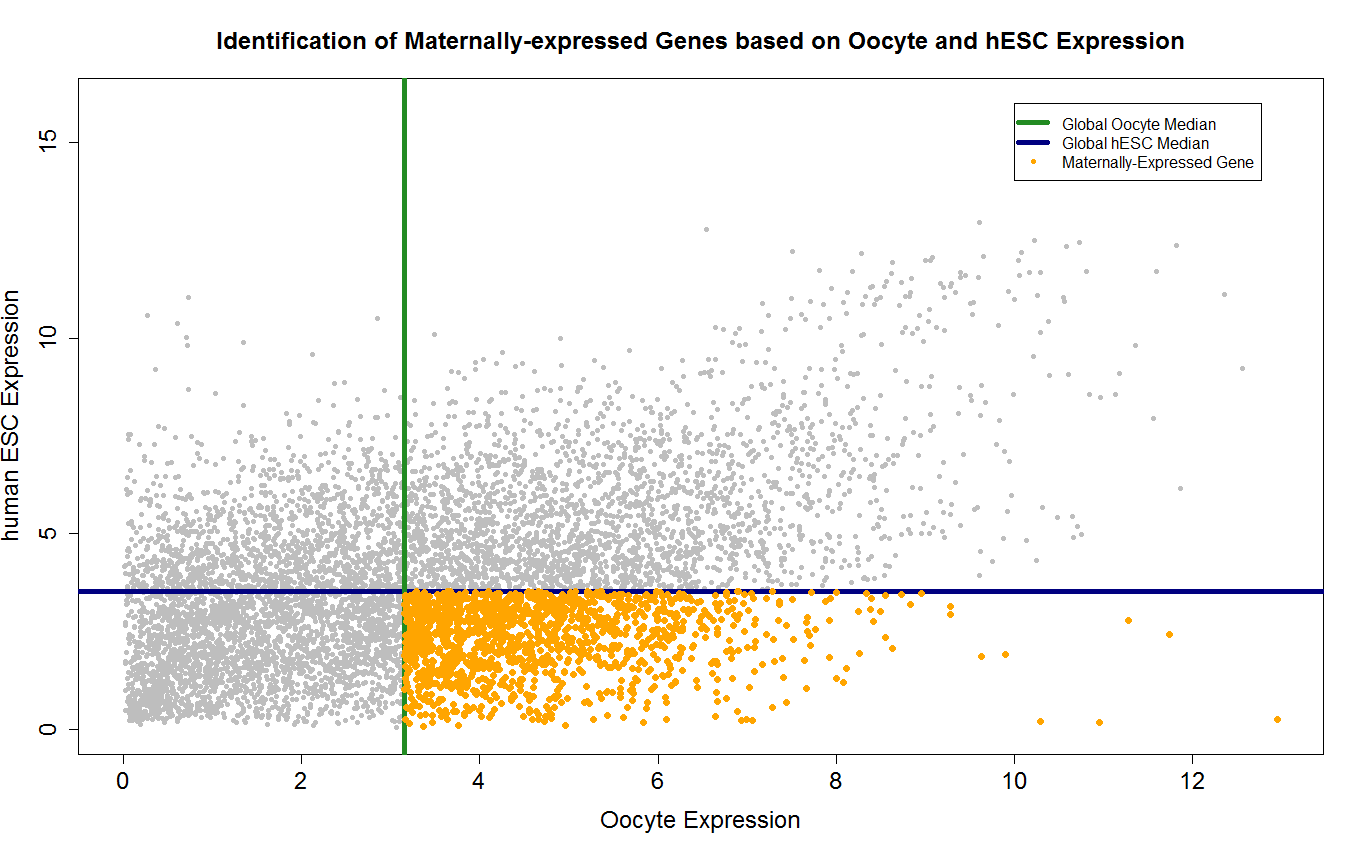


*Determining which maternally-expressed genes were most likely contributed to the zygote by focusing on the genes with stable, invariant expression at the 4-cell stage.*

A subset of genes that are highly expressed with high stability (invariance) in the early stages of development in the zygote are likely to be contributed by the maternal transcriptome. Therefore, we determined the overlap between genes that we had identified as stably expressed, and having either high or medium average expression at the 4-cell stage. In total, we found **90 stable genes** that were likely to be contributed by the maternal transcriptome.

**Figure 3.** Overlap of maternally-expressed genes and the stable genes at the 4-cell stage.


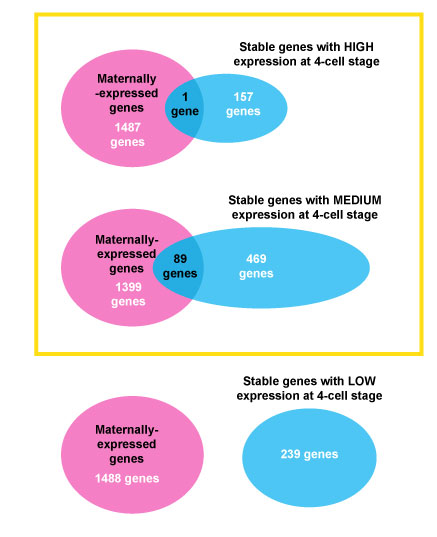


The stable gene that was expressed in the maternal transcriptome and highly expressed at the 4-cell stage was DPPA3, a known maternal factor in mice and has an important role in development of preimplantation embryos [2]. More recently in human ESCs, DPPA3, also known as STELLA, has been found to facilitate the differentiation of the germline and endodermal lineages [3]. For the Yan et al. data set, we see that the single cell expression distribution of DPPA3 showed elevated and stable expression of this gene through most stages of development until the blastocyst stage, and then a marked drop in expression for the two hESC populations.

**Figure 4.** Gene expression distribution in single cells of the maternal factor DPPA3. Each circle represents expression of DPPA3 in a single cell.


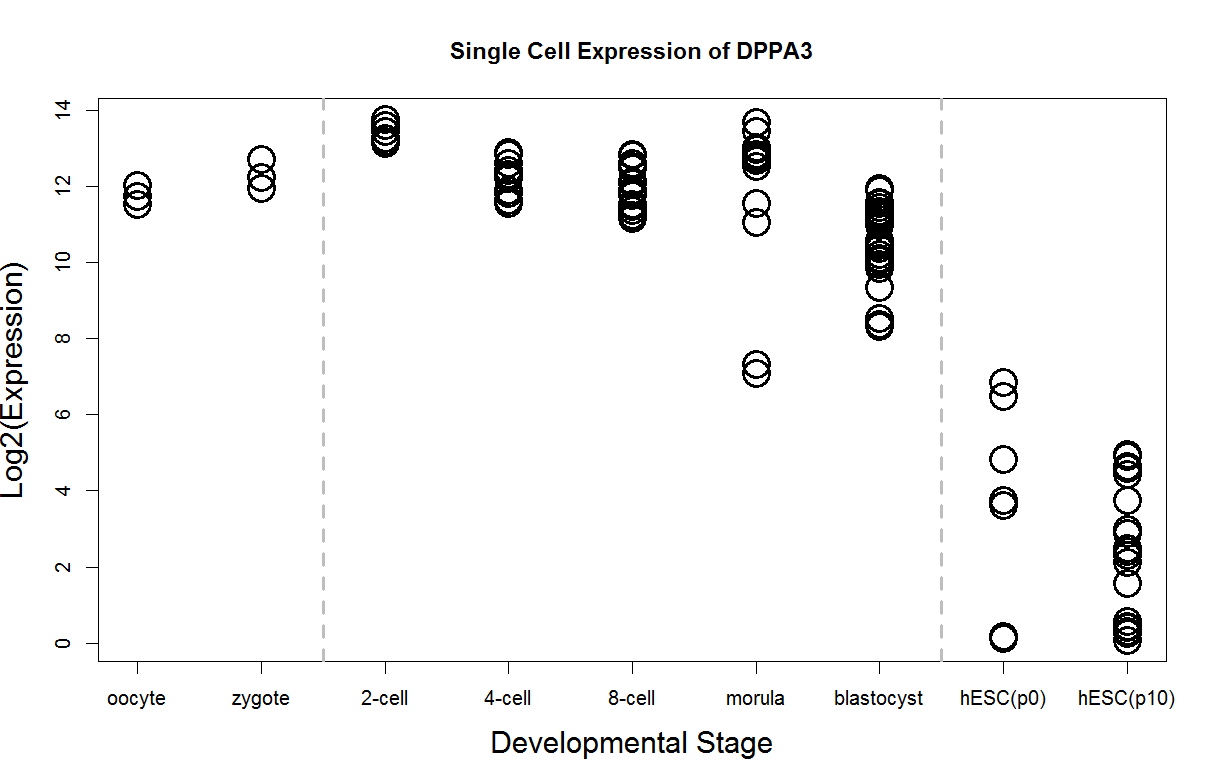


*Identification of stable genes that are expressed in the early zygotic stage and not contributed by the maternal transcriptome.*

As part of the developmental switches that occur during maternal-to-zygotic transition (MZT), genes that are exclusively expressed in the zygote and not contributed by the maternal transcriptome, are transcriptionally activated to facilitate Zygote Genome Activation (ZGA). From the previous analysis, we identified the maternally-contributed genes however we can also use these lists to infer the genes that are likely to be transcriptionally active in the early zygote. These early zygote genes were identified as the subset of genes that are not in the maternally-expressed gene, and exclusively in the set of stable genes that have either high or medium average levels of expression at the 4-cell stage. In total, we identified 626 genes (157 with high average expression, 469 with medium average expression).

From studies performed on zebrafish embryos, it is known that the early zygote genes are enriched for targets of the main pluripotency factors, of which their human orthologs are OCT4, SOX2 and NANOG [4, 5].

For our list of early zygotic genes that were transcriptionally active, we saw an enriched of genes that are targets of SOX2 and NANOG but not OCT4 (P-value < 0.05). We obtained target information from a study performed in human embryonic stem cells [6] and assessed the significance of the overlap using a Fisher’s exact test. We used the list of stable genes that had low average expression at the 4-cell stage as an appropriate background to test for enrichment in our list of transcriptionally active, early zygotic genes.

**Table 1.** Counts of early zygotic genes and targets of OCT4.

|  | *OCT4 Targets* | *Not OCT4 Targets* |
| --- | --- | --- |
| Early Zygotic Genes (Transcriptionally Active) | **14** | 612 |
| Early Zygotic Genes (Transcriptionally Repressed) | 6 | 233 |

**Table 2.** Counts of early zygotic genes and targets of SOX2.

|  | *SOX2 Targets* | *Not SOX2 Targets* |
| --- | --- | --- |
| Early Zygotic Genes (Transcriptionally Active) | **47** | 579 |
| Early Zygotic Genes (Transcriptionally Repressed) | 6 | 233 |

**Table 3.** Counts of early zygotic genes and targets of NANOG.

|  | NANOG Targets | Not NANOG Targets |
| --- | --- | --- |
| Early Zygotic Genes (Transcriptionally Active) | **53** | 573 |
| Early Zygotic Genes (Transcriptionally Repressed) | 9 | 230 |

**Table 4.** Summary statistics from a two-sided Fisher’s exact test to evaluate enrichment of pluripotency transcription factor targets amongst genes that are transcriptionally active in the early zygote.

| ***Early Zygotic Genes (Transcriptionally Active)*** | **OCT4 Targets** | **SOX2 Targets** | **NANOG Targets** |
| --- | --- | --- | --- |
| **Overlap with Targets** | 14 | 47 | 53 |
| **Odds Ratio** | 0.8885 | 3.1491 | 2.3618 |
| **95% Confidence Interval** | 0.3161178 2.8554480 | 1.318982 9.136188 | 1.130727 5.537746 |
| **P-value (Exact Test)** | 0.8027 | 0.006339 | 0.01764 |
| **P-value (Permutation Test)** | 0.5766 | 0.0023 | 0.0228 |

We ran 10,000 permutations to assess the probability of observing something more extreme than the Odds Ratio obtained for each set of TF targets. Gene sets of identical size to the list of repressed and active early zygotic genes were sampled at random from the starting set of 8105 genes. For SOX2 and NANOG, even after permutation, we saw significant enrichment (P-value < 0.05).

**Figure 5.** Distribution of permutation P-values generated for each set of transcriptional factor targets. The red line indicates the P-value observed with real, un-permutated data.


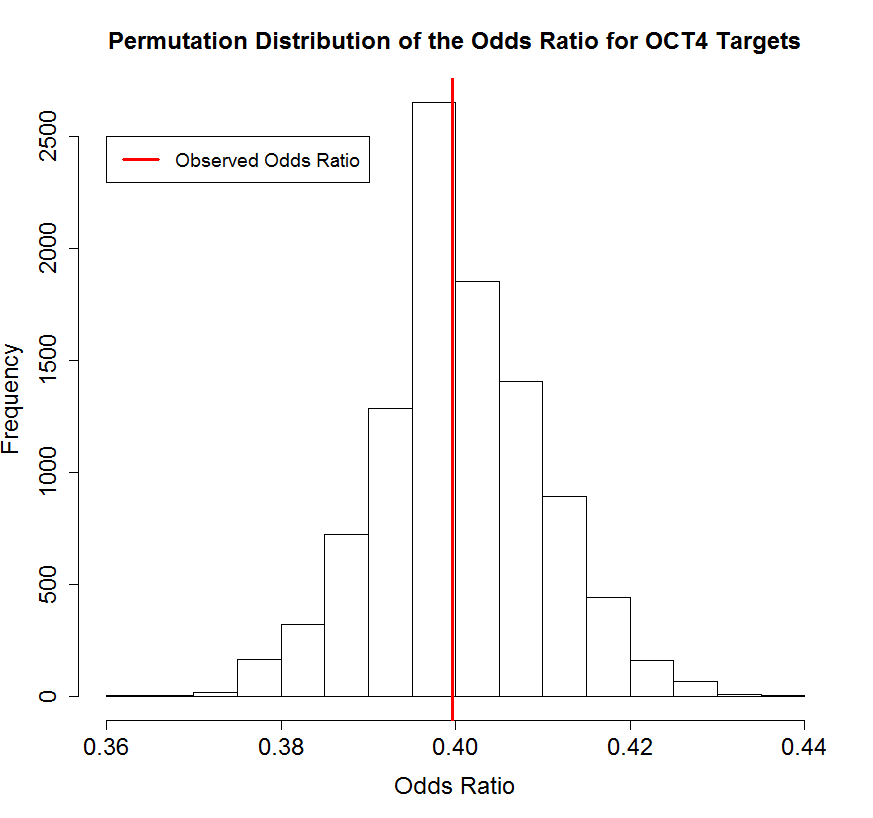


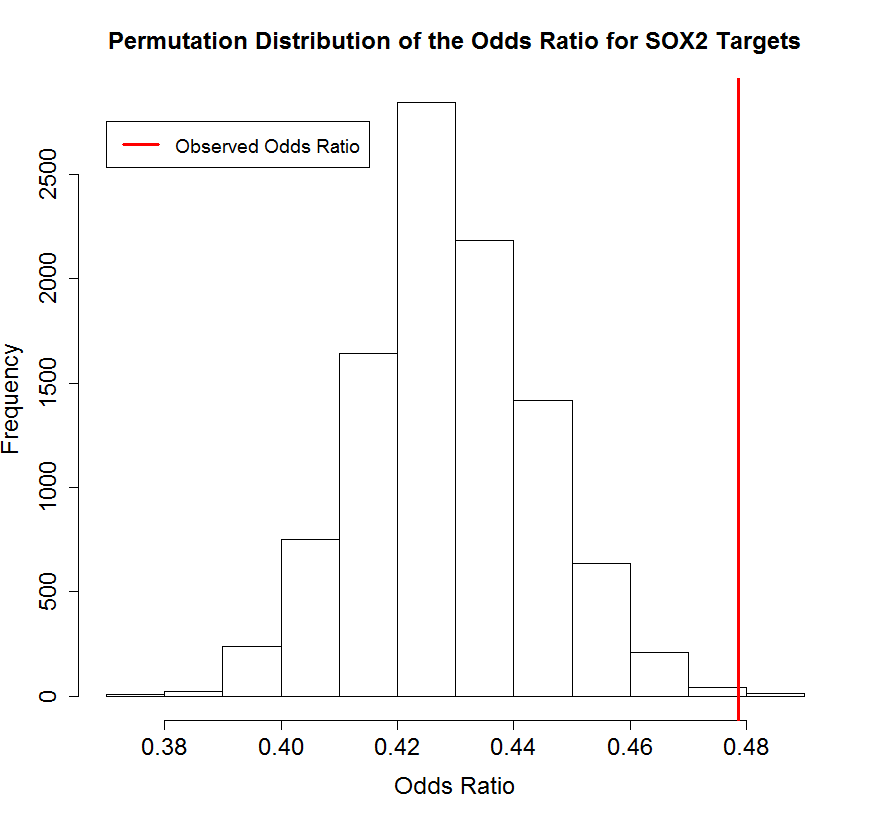

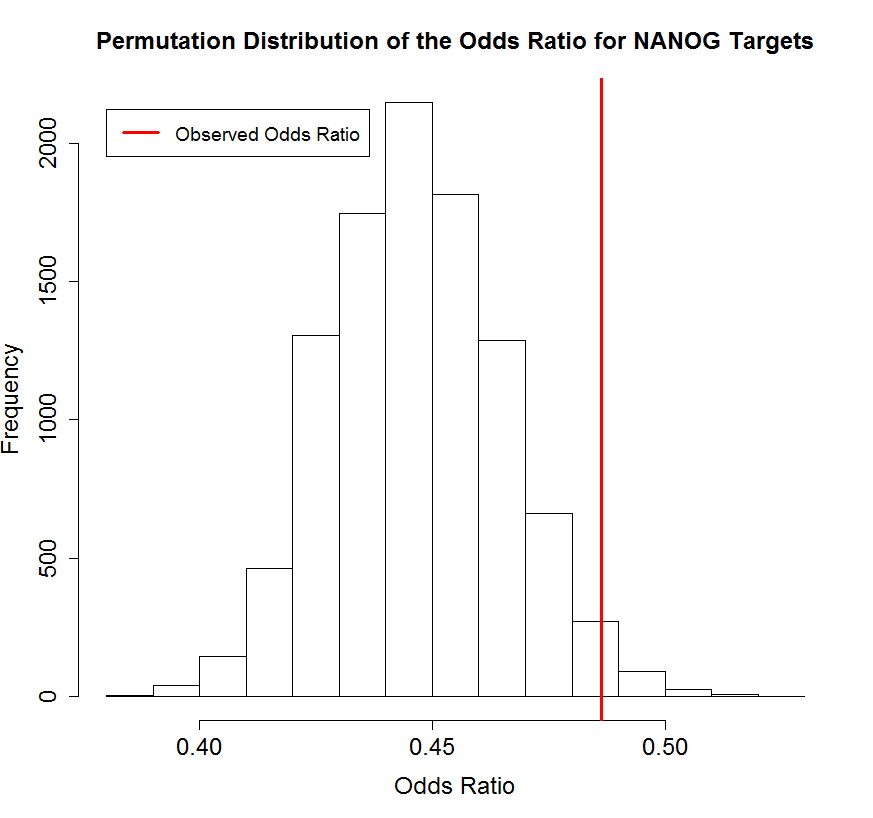


*Functional Enrichment of the Maternally-Contributed Genes*

For the 90 genes with overlapping expression values between the maternal and the 4-cell set, we also tested for enrichment of Biological Process terms using the Metacore software package. The terms that were statistically significant (FDR-adjusted P-value < 0.01) are listed in **Table 5** below.

Even though these 90 genes were identified by expression overlap there is no guarantee that the transcripts represented in the embryo are also maternal, as gene activation within the embryo could replace maternal transcripts with the same gene product. Nevertheless, prior publications have presented evidence for multiple roles of the genes found in the table with embryonic development. For example, adenylate cyclase has been found to play a role in regulation of processes in both oocytes and embryos. PDK -/- embryonic stem cells have dis-regulated transcription [7]. Previous studies have also found that p300 is required for normal embryogenesis [8].

**Table 5.** List of statistically significant Biological Process terms that were enriched in the maternally-contributed genes.

| Biological Process | Adjusted P-value | Genes |
| --- | --- | --- |
| Signal transduction: ESR1-membrane pathway | 0.002 | Adenylate cyclase, G-protein alpha-13, Adenylate cyclase type VIII, PDK (PDPK1), p300, Adenylate cyclase type III |
| Signal transduction: ESR1-nuclear pathway | 0.017 | Adenylate cyclase, Adenylate cyclase type VIII, PDK (PDPK1), LBC, p300, Adenylate cyclase type III, LCoR (MLR2) |
| Signal Transduction: Cholecystokinin signaling | 0.017 | Adenylate cyclase, G-protein alpha-13, ATF-2, LBC, G-protein alpha-12 family |
| Apoptosis:_Anti-Apoptosis mediated by external signals via PI3K/AKT | 0.017 | Adenylate cyclase, FOXO3A, PDK (PDPK1), G-protein alpha-12 family, c-IAP1, GDNF, c-IAP2 |
| Proteolysis: Proteolysis in cell cycle and apoptosis | 0.025 | Skp2/TrCP/FBXW, beta-TrCP, Separase, c-IAP1, c-IAP2 |

Based on all the analyses described in this supplement, we have made the following gene lists available:

- List of Genes that are Contributed by the Maternal Transcriptome
- List of Genes that are Contributed by the Zygote and Transcriptionally Active
- List of Genes that are Contributed by the Zygote and Transcriptionally Repressed

**References**

1. Yan L, Yang M, Guo H, Yang L, Wu J, Li R, et al. Single-cell RNA-Seq profiling of human preimplantation embryos and embryonic stem cells. Nat Struct Mol Biol. 2013;20(9):1131-9. doi: 10.1038/nsmb.2660. PubMed PMID: 23934149.

2. Payer B, Saitou M, Barton SC, Thresher R, Dixon JP, Zahn D, et al. Stella is a maternal effect gene required for normal early development in mice. Curr Biol. 2003;13(23):2110-7. PubMed PMID: 14654002.

3. Wongtrakoongate P, Jones M, Gokhale PJ, Andrews PW. STELLA facilitates differentiation of germ cell and endodermal lineages of human embryonic stem cells. PLoS One. 2013;8(2):e56893. doi: 10.1371/journal.pone.0056893. PubMed PMID: 23457636; PubMed Central PMCID: PMC3573007.

4. Lee MT, Bonneau AR, Takacs CM, Bazzini AA, DiVito KR, Fleming ES, et al. Nanog, Pou5f1 and SoxB1 activate zygotic gene expression during the maternal-to-zygotic transition. Nature. 2013;503(7476):360-4. doi: 10.1038/nature12632. PubMed PMID: 24056933; PubMed Central PMCID: PMC3925760.

5. Leichsenring M, Maes J, Mossner R, Driever W, Onichtchouk D. Pou5f1 transcription factor controls zygotic gene activation in vertebrates. Science. 2013;341(6149):1005-9. doi: 10.1126/science.1242527. PubMed PMID: 23950494.

6. Boyer LA, Lee TI, Cole MF, Johnstone SE, Levine SS, Zucker JP, et al. Core transcriptional regulatory circuitry in human embryonic stem cells. Cell. 2005;122(6):947-56. doi: 10.1016/j.cell.2005.08.020. PubMed PMID: 16153702; PubMed Central PMCID: PMC3006442.

7. Tominaga Y, Tamguney T, Kolesnichenko M, Bilanges B, Stokoe D. Translational deregulation in PDK-1-/- embryonic stem cells. Mol Cell Biol. 2005;25(19):8465-75. doi: 10.1128/MCB.25.19.8465-8475.2005. PubMed PMID: 16166629; PubMed Central PMCID: PMC1265760.

8. Kwok RP, Liu XT, Smith GD. Distribution of co-activators CBP and p300 during mouse oocyte and embryo development. Mol Reprod Dev. 2006;73(7):885-94. doi: 10.1002/mrd.20440. PubMed PMID: 16596650.
