# Supplementary material for: Variability of Gene Expression Identifies Transcriptional Regulators of Early Human Embryonic Development
Source: PLoS Genet. 2015 Aug 19;11(8):e1005428. doi: 10.1371/journal.pgen.1005428 (PMC4546122; doi:10.1371/journal.pgen.1005428)
Supplement: S5 Text — (DOCX) [file pgen.1005428.s012.docx]

**Text S5. Assessment of the functional impact of expression variability on human embryonic development.**

A meta-analysis based on data from seven genome-wide, large-scale human and mouse studies was performed to assess how expression variability may functionally impact transcriptional regulation of the human embryo. We reasoned that if the stable genes identified through an analysis of expression variability were important to embryonic development then we should expect to see a significant overlap of these genes with those that had previously been linked to phenotypes related to essentiality, lethality or ubiquitous expression. Similarly, we would also expect to see enrichment amongst stable genes with genes that have been implicated in human genetic diseases, since we hypothesize that the stable genes are core regulators of the embryo and therefore their perturbation would therefore result in a disease phenotype. For a healthy human population, mutations in essential genes would be less likely to occur and selected against due to background selection. Hence, we would expect to see a reduced overlap in genes associated with loss-of-function variants with the set of stable genes if indeed the stable genes represent core, essential regulators.

To test this hypothesis, the studies used were:

1. **Online Mendelian Inheritance in Man (OMIM)** **Gene Map** [1] represents a catalogue of human genes that have been linked to a Mendelian trait or disorder. We extracted the genes via the Download site located at: http://www.omim.org/downloads and used the *genemap.txt* file.
2. **A Catalog of Published Genome-Wide Association Studies (GWAS Catalog)** [2] contains all genes that have been published in a GWAS. The data was downloaded from the EBI site: [www.ebi.ac.uk/gwas/](http://www.ebi.ac.uk/gwas/), specifically the file gwas_catalog_v1.0-donloaded_2015-05-06.tsv. We extracted all genes from the Mapped Gene column which reported the genes that had a SNP identified in a published GWAS. For SNPs identified in an intergenic region, we included both the upstream and downstream genes that were reported.
3. A list of 2,472 **human orthologs of mouse essential genes** was curated by Georgi et al. [3]. Using the Mouse Genome Informatics database, Georgi et al. identified genes associated with 46 phenotypic categories based on terms associated with pre-, peri- and postnatal lethality. The GENCODE database was then used to map the essential mouse genes to their one-to-one human orthologs.
4. A list of **ubiquitously expressed human genes** were determined by de Jonge et al. [4]. Using combined data from 13,629 human gene array samples, de Jonge et al. ranked all human genes based on their stability in gene expression using the coefficient of variation. We extracted the top 10% most stable genes for our analysis, resulting in a list of 1,304 human genes.
5. A list of **299 human haploinsufficient genes** were identified by Dang et al. [5] based on a rigorous analysis of the published literature, and the OMIM database [1].
6. A list of **loss-of-function genetic variants** **in human protein-coding genes** from MacArthur et al. [6] was determined through a series of stringent filters applied to the 1000 Genomes data to result in a curated list of high-confidence hits. The file was downloaded from: <http://ftp.1000genomes.ebi.ac.uk/vol1/ftp/phase1/analysis_results/functional_annotation/annotated_vcfs/ALL.wgs.integrated_phase1_release_v3_Loss_of_Function_20120626.20101123.xls>.
7. A list of human recessive disease genes from Boone et al. [7] that were associated with a copy number variant deletion spanning two or more recessive disease genes.

As an appropriate background to compare enrichment to, we used the 18,992 human protein-coding genes currently annotated in the HUGO Gene Nomenclature Committee list [8]. The data was downloaded from http://www.genenames.org/cgi-bin/statistics, and the file used was *gene_with_protein_product.txt*. For each study, we calculated the enrichment of stable genes that overlapped with the genes of interest identified and assessed its significance using a two-sided Fisher’s exact test.

**Figure 1:** Schematic outline detailing how the overlap between the list of stable genes and the list of category genes is assessed using Fisher’s exact test.
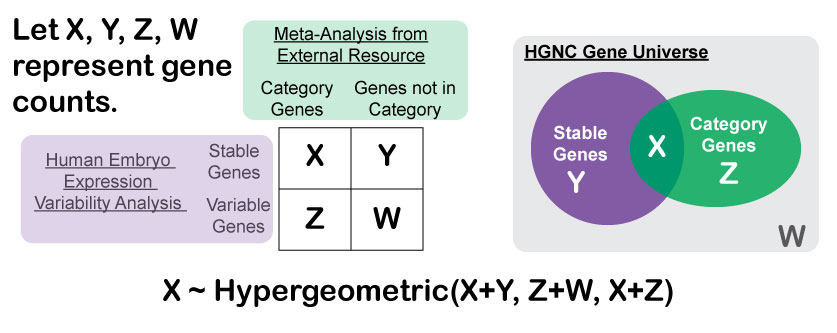


For each of the seven studies, we computed the following gene counts:

| **#1.** | **OMIM Genes** | **Other HGNC Genes** |
| --- | --- | --- |
| **Stable Genes** | 601 | 190 |
| **Variable Genes** | 4637 | 1733 |

| **#2.** | **GWAS Catalog Genes** | **Other HGNC Genes** |
| --- | --- | --- |
| **Stable Genes** | 257 | 543 |
| **Variable Genes** | 1981 | 4426 |

| **#3.** | **Essential Genes** | **Other HGNC Genes** |
| --- | --- | --- |
| **Stable Genes** | 166 | 626 |
| **Variable Genes** | 1109 | 5274 |

| **#4.** | **Top 10% Ubiquitously Expressed Genes** | **Other HGNC Genes** |
| --- | --- | --- |
| **Stable Genes** | 225 | 569 |
| **Variable Genes** | 782 | 5627 |

| **#5.** | **Haploinsufficient Genes** | **Other HGNC Genes** |
| --- | --- | --- |
| **Stable Genes** | 17 | 774 |
| **Variable Genes** | 84 | 6286 |

| **#6.** | **Loss-of-Function Variant Genes** | **Other HGNC Genes** |
| --- | --- | --- |
| **Stable Genes** | 138 | 660 |
| **Variable Genes** | 1357 | 5101 |

| **#7.** | **Recessive Disease Genes** | **Other HGNC Genes** |
| --- | --- | --- |
| **Stable Genes** | 34 | 473 |
| **Variable Genes** | 757 | 5897 |

**Table 1:** Assessing enrichment of stable genes with different functional categories.

| **Stable Genes**  **2-sided Exact Test** | **Functional Category** | **P-value** | **Odds Ratio Estimate** | **95% Confidence Interval** |
| --- | --- | --- | --- | --- |
| 1 | OMIM Gene Map | 0.06121 | 1.182134 | (0.9929463, 1.4120945) |
| 2 | GWAS Catalog | 0.4911 | 1.057442 | (0.8995706, 1.2407526) |
| 3 | Essential Gene List (Georgi et al.) | 0.01376 | 1.261036 | (1.043609, 1.517813) |
| 4 | Top 10% Ubiquitously Expressed Genes  (de Jonge et al.) | < 2.2e-16 | 2.844841 | 2.384891 3.386566 |
| 5 | Haploinsufficient Genes (Dang et al.) | 0.07628 | 1.643481 | 0.909576 2.808734 |
| 6 | Loss of Function Variants  (MacArthur et al.) | 0.01392 | 0.7860137 | 0.6431917 0.9553694 |
| 7 | Recessive Disease Genes (Boone et al.) | 0.0008947 | 0.5599949 | 0.3800937 0.8012453 |
|  |  |  |  |  |
| Legend | P-value < 0.10 | P-value < 0.05 |  |  |

We observed that for most of these categories, the overlap with stable genes identified from our analysis was statistically significant (P-value < 0.10), suggesting that our list of genes was associated with genes that had previously been determined to be essential or functionally important. Only the GWAS Catalog did not yield a statistically-significant overlap with the stable genes. This is likely to be the result of the diverse range of phenotypes that are included in the GWAS Catalog, not all of which has strong genetic links and also may not be so directly relevant to developing embryos or lethality.

**References**

1. 1. Online Mendelian Inheritance in Man, OMIM®. McKusick-Nathans Institute of Genetic Medicine, Johns Hopkins University (Baltimore, MD). Available from: <http://omim.org/>.
2. 2. Welter D, MacArthur J, Morales J, Burdett T, Hall P, Junkins H, et al. The NHGRI GWAS Catalog, a curated resource of SNP-trait associations. Nucleic Acids Res. 2014;42(Database issue):D1001-6. doi: 10.1093/nar/gkt1229. PubMed PMID: 24316577; PubMed Central PMCID: PMC3965119.
3. 3. Georgi B, Voight BF, Bucan M. From mouse to human: evolutionary genomics analysis of human orthologs of essential genes. PLoS Genet. 2013;9(5):e1003484. doi: 10.1371/journal.pgen.1003484. PubMed PMID: 23675308; PubMed Central PMCID: PMC3649967.
4. 4. de Jonge HJ, Fehrmann RS, de Bont ES, Hofstra RM, Gerbens F, Kamps WA, et al. Evidence based selection of housekeeping genes. PLoS One. 2007;2(9):e898. doi: 10.1371/journal.pone.0000898. PubMed PMID: 17878933; PubMed Central PMCID: PMC1976390.
5. 5. Dang VT, Kassahn KS, Marcos AE, Ragan MA. Identification of human haploinsufficient genes and their genomic proximity to segmental duplications. Eur J Hum Genet. 2008;16(11):1350-7. doi: 10.1038/ejhg.2008.111. PubMed PMID: 18523451.
6. 6. MacArthur DG, Balasubramanian S, Frankish A, Huang N, Morris J, Walter K, et al. A systematic survey of loss-of-function variants in human protein-coding genes. Science. 2012;335(6070):823-8. doi: 10.1126/science.1215040. PubMed PMID: 22344438; PubMed Central PMCID: PMC3299548.
7. 7. Boone PM, Campbell IM, Baggett BC, Soens ZT, Rao MM, Hixson PM, et al. Deletions of recessive disease genes: CNV contribution to carrier states and disease-causing alleles. Genome Res. 2013;23(9):1383-94. doi: 10.1101/gr.156075.113. PubMed PMID: 23685542; PubMed Central PMCID: PMC3759716.
8. 8. Gray KA, Yates B, Seal RL, Wright MW, Bruford EA. Genenames.org: the HGNC resources in 2015. Nucleic Acids Res. 2015;43(Database issue):D1079-85. doi: 10.1093/nar/gku1071. PubMed PMID: 25361968; PubMed Central PMCID: PMC4383909.
