# Supplementary material for: Variability of Gene Expression Identifies Transcriptional Regulators of Early Human Embryonic Development
Source: PLoS Genet. 2015 Aug 19;11(8):e1005428. doi: 10.1371/journal.pgen.1005428 (PMC4546122; doi:10.1371/journal.pgen.1005428)

**Text S6. Inspecting the expression of the blastocyst variability markers in human iPSCs and ESCs from other studies.**

In order to determine whether the increased average expression we observed in the blastocyst markers was not just a function of the environment that the embryos were exposed to, we looked at the expression of these genes in other stem cell lines. We obtained expression data from three other published studies and also the two hESC populations that were profiled in the Yan data set. The data sets and studies that they derive from are described briefly in Table 1.

**Table 1.** Information on the studies used to validate the expression of the blastocyst variability markers.

| *Study* | *Reference* | *Cell Lines* | *Experimental Design* | *Platform* | *Data Access* | *Pre-processing Steps Applied* |
| --- | --- | --- | --- | --- | --- | --- |
| [1] | Kolle et al. (2009) | hESCs (HES2 cell line) | Sorted populations based on four different levels of pluripotency. Three replicates per pluripotent fraction. A total of 12 samples. | Illumina Human-6 v2 BeadChip Array | GEO GSE13201 |  |
| [2] | Vitale et al. (2012) | hESCs, two iPSCs (SSEA-4 high and SSEA-4 low), fibroblasts | Eight fibroblast lines, three hESCs, 5 iPSCs (SSEA-4 high), 4 iPSCs (SSEA-4 low). | Illumina HT12 v4 BeadChip Array | Array Express E-MTAB-1040 | Expression values with a detection  P-value > 0.01 were removed from the analysis. |
| [3] | Briggs et al. (2013) | hESCs (MEL1 cell line), iPSCs, fibroblasts | 15 fibroblast lines, 3 hESCs (MEL1), 15 iPSCs all from normal control subjects. | Illumina HT12 v4 BeadChip Array | GEO GSE42956 |  |
| [4] | Yan et al. (2013) | hESCs at passage 0 and passage 10 | 8 hESCs (passage 0), 26 hESCs (passage 10). | RNA-seq | GEO GSE36552 | Expression values RPKM < 0.1 were removed from the analysis. |

**References**

1. Kolle G, Ho M, Zhou Q, Chy HS, Krishnan K, Cloonan N, et al. Identification of human embryonic stem cell surface markers by combined membrane-polysome translation state array analysis and immunotranscriptional profiling. Stem Cells. 2009;27(10):2446-56. doi: 10.1002/stem.182. PubMed PMID: 19650036.

2. Vitale AM, Matigian NA, Ravishankar S, Bellette B, Wood SA, Wolvetang EJ, et al. Variability in the generation of induced pluripotent stem cells: importance for disease modeling. Stem Cells Transl Med. 2012;1(9):641-50. doi: 10.5966/sctm.2012-0043. PubMed PMID: 23197870; PubMed Central PMCID: PMC3659735.

3. Briggs JA, Sun J, Shepherd J, Ovchinnikov DA, Chung TL, Nayler SP, et al. Integration-free induced pluripotent stem cells model genetic and neural developmental features of down syndrome etiology. Stem Cells. 2013;31(3):467-78. doi: 10.1002/stem.1297. PubMed PMID: 23225669.

4. Yan L, Yang M, Guo H, Yang L, Wu J, Li R, et al. Single-cell RNA-Seq profiling of human preimplantation embryos and embryonic stem cells. Nat Struct Mol Biol. 2013;20(9):1131-9. doi: 10.1038/nsmb.2660. PubMed PMID: 23934149.

**Figure 1.** Expression of the blastocyst variability markers in the Kolle et al. data set. Each circle represents the gene expression in a hESC line for a specific pluripotent fraction (p4 corresponds to the least pluripotent fraction, p7 is the most pluripotent).


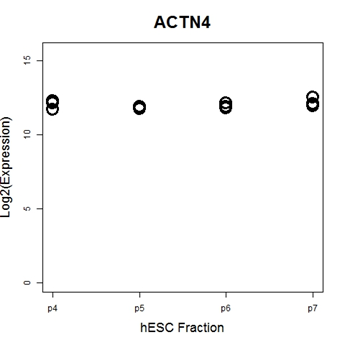

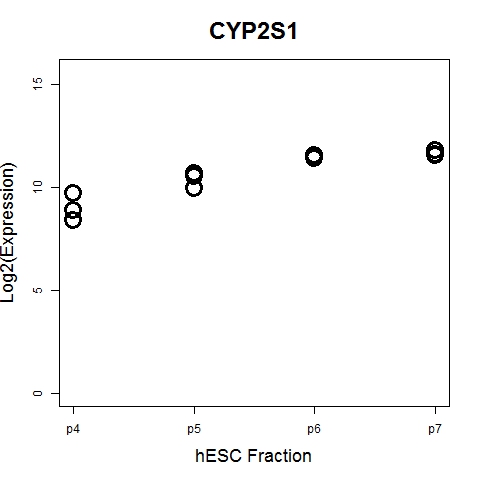

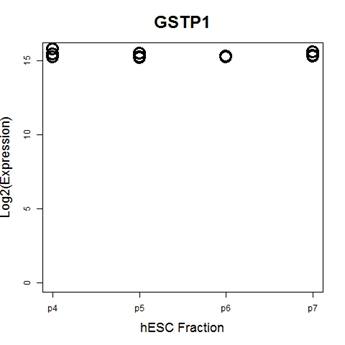


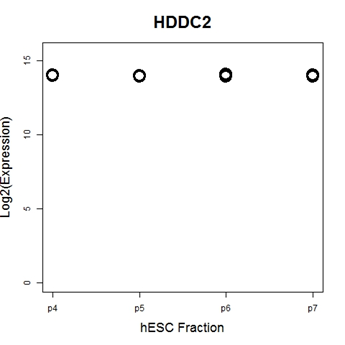

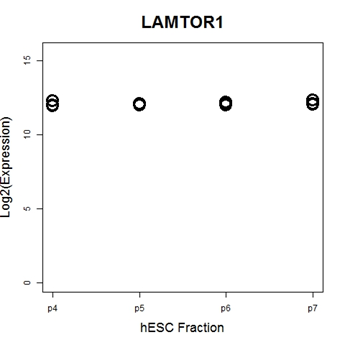

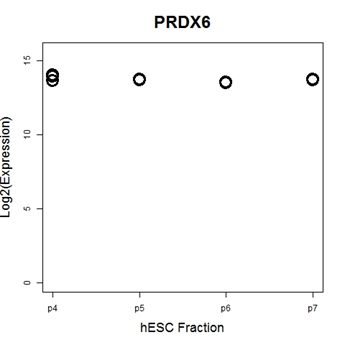


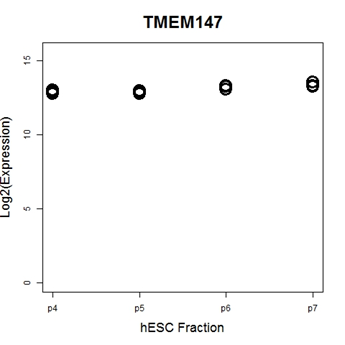


**Figure 2.** Expression of the blastocyst variability markers in the Vitale et al. data set.


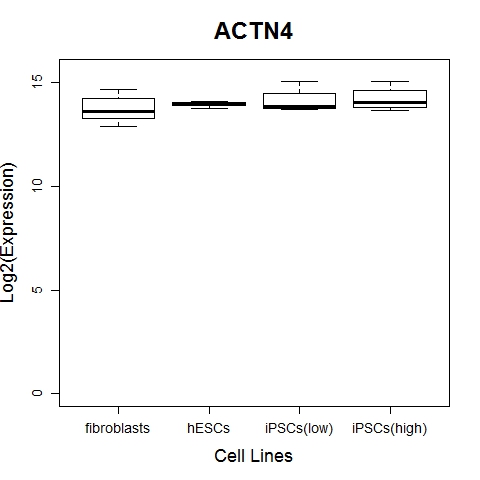

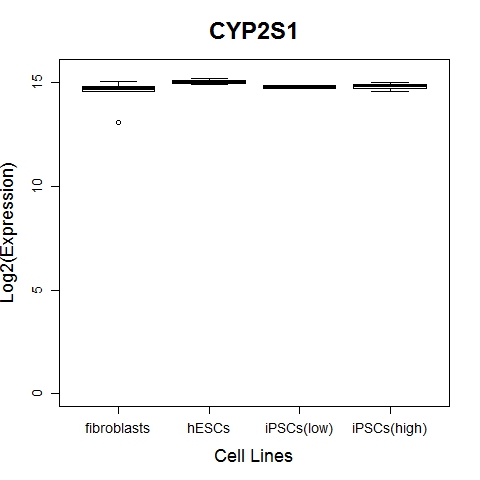

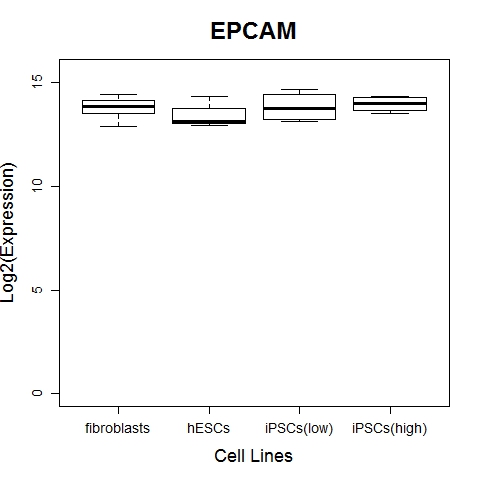

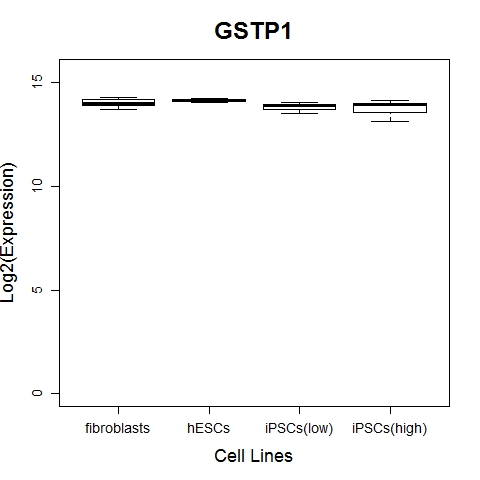

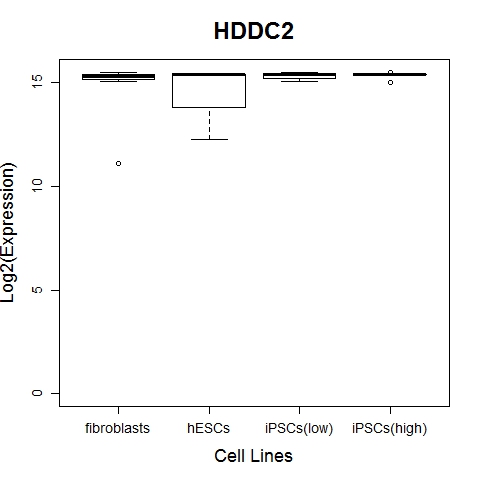

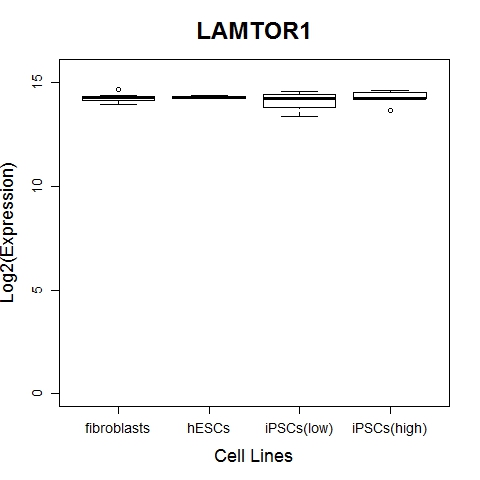

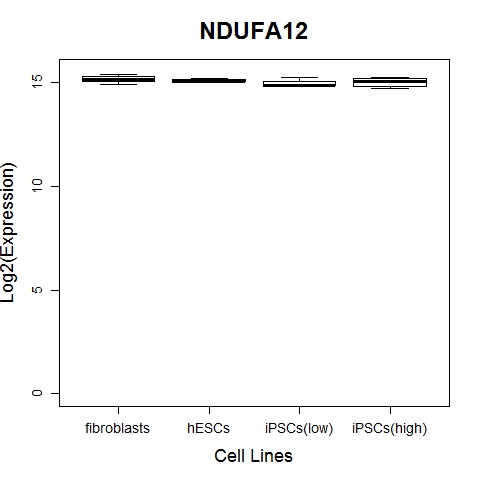

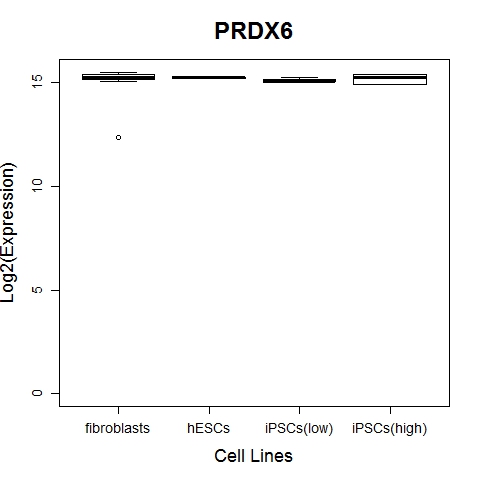

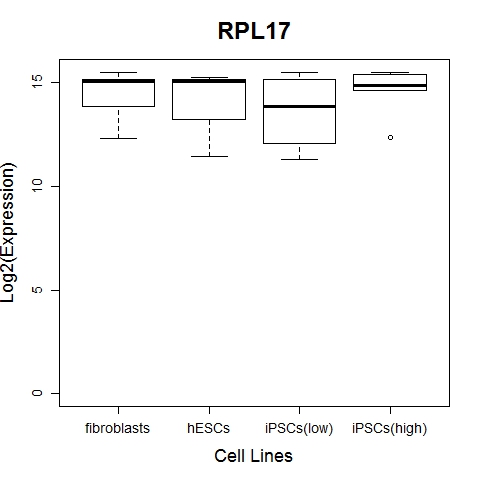

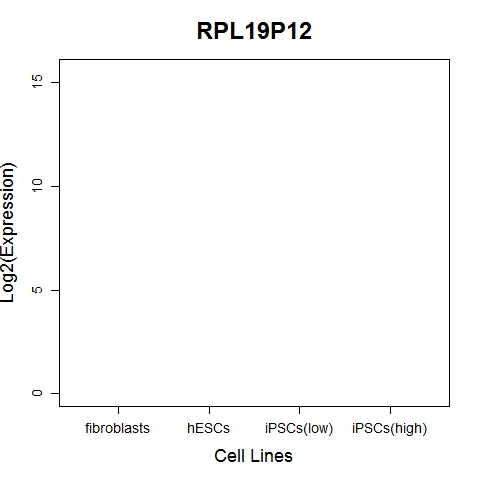

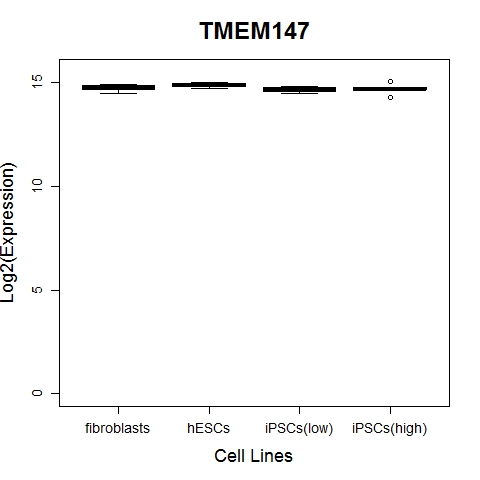


**Figure 3.** Expression of the blastocyst variability markers in the Briggs et al. data set.


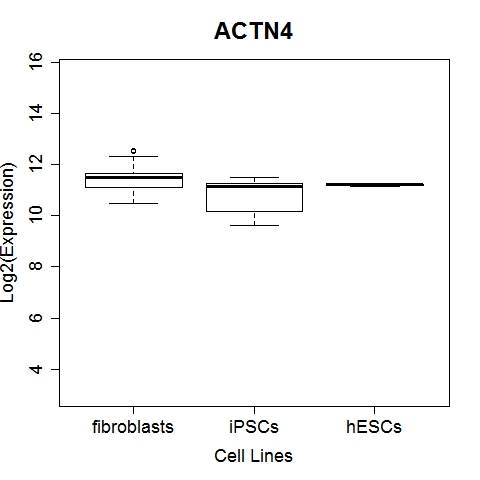

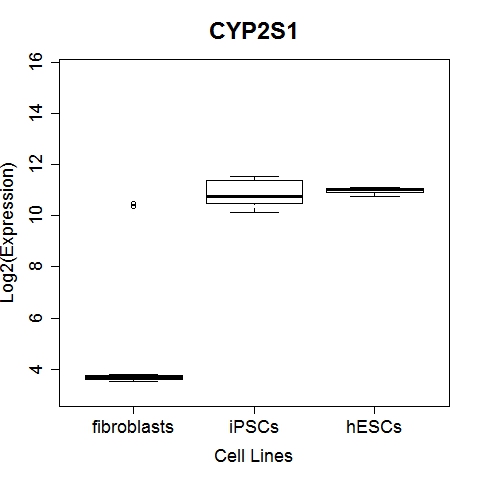

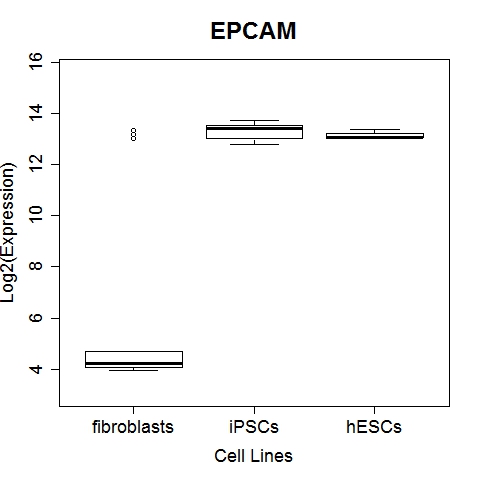

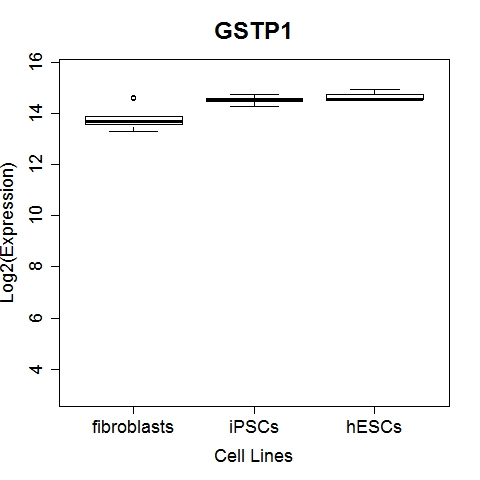

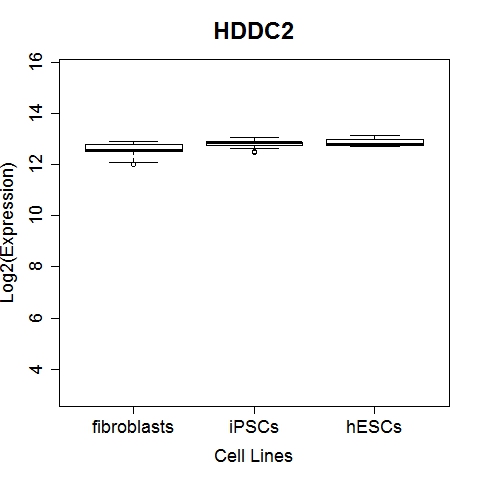

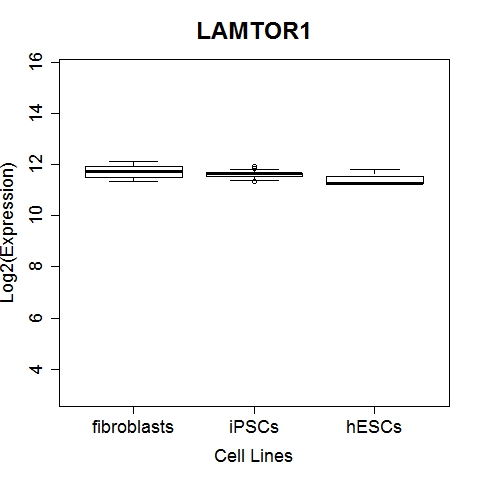

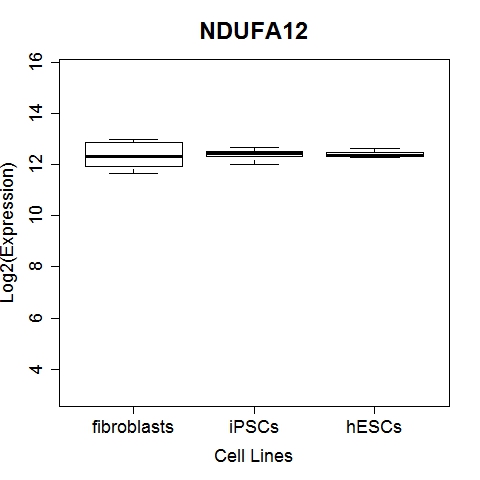

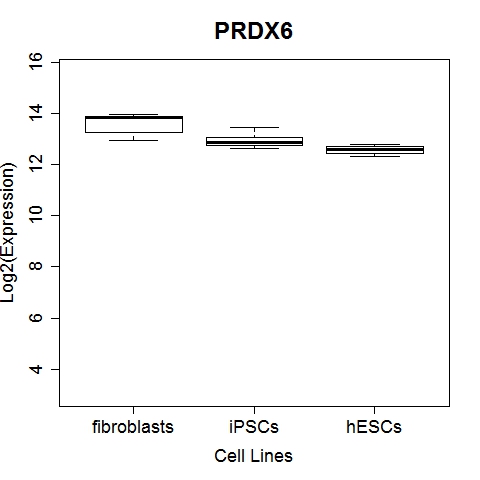

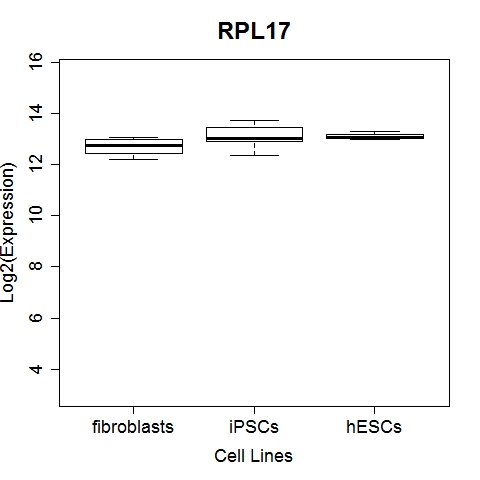

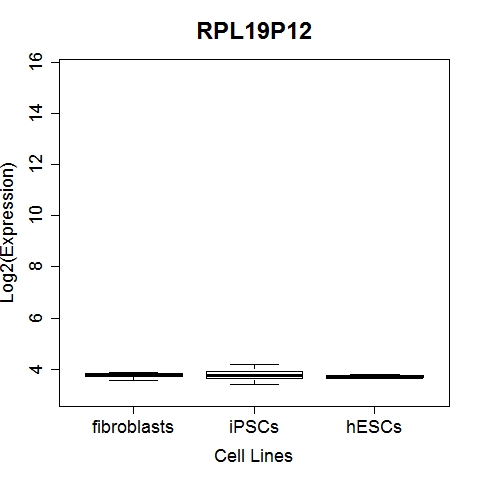

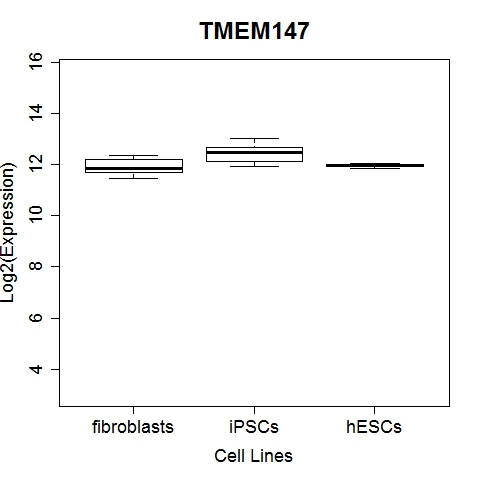


**Figure 4.** Expression of the blastocyst variability markers in the two hESC populations (highlighted in gray) from the Yan et al. data set.


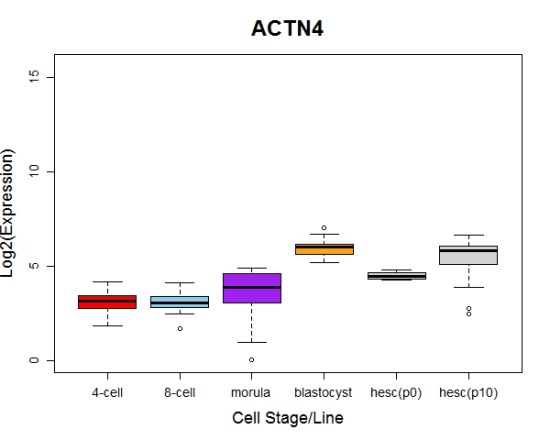

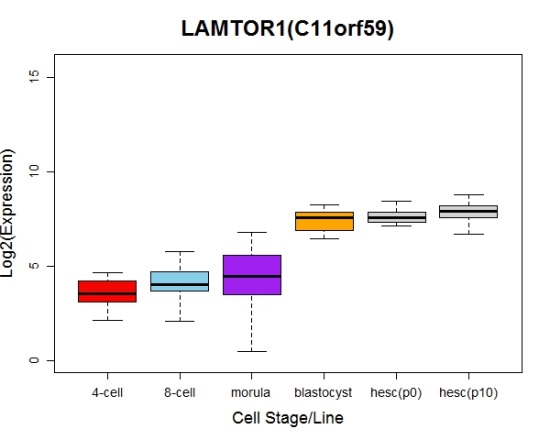

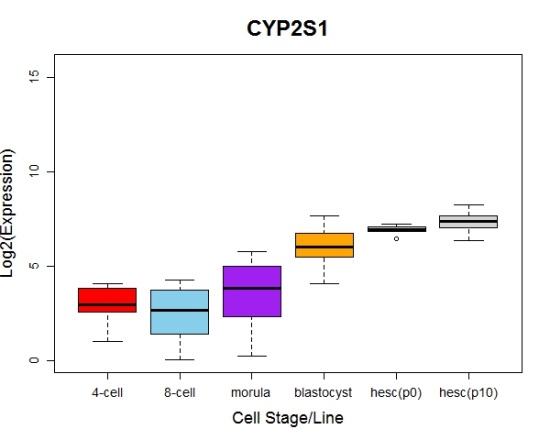

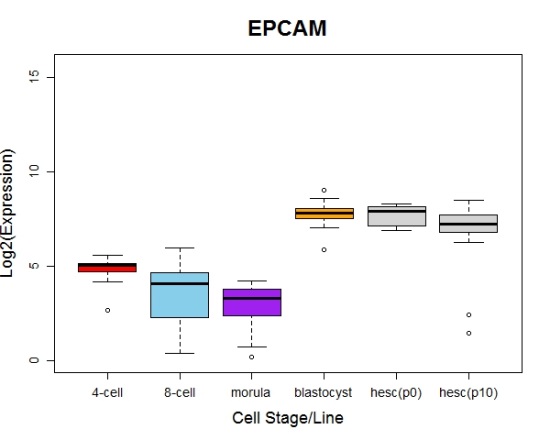

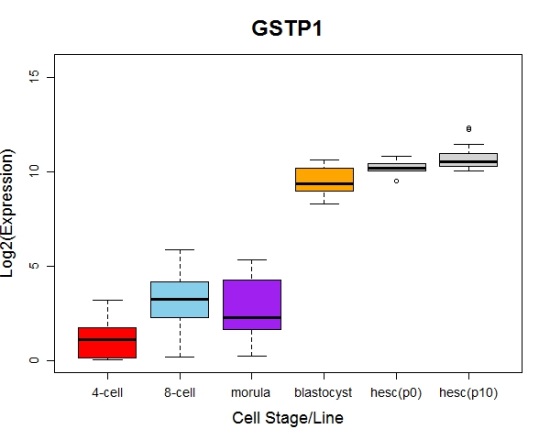

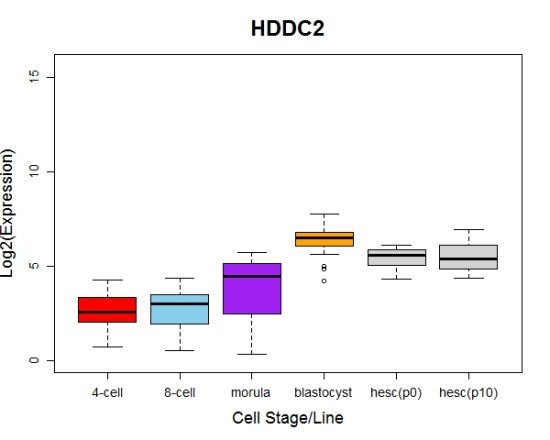

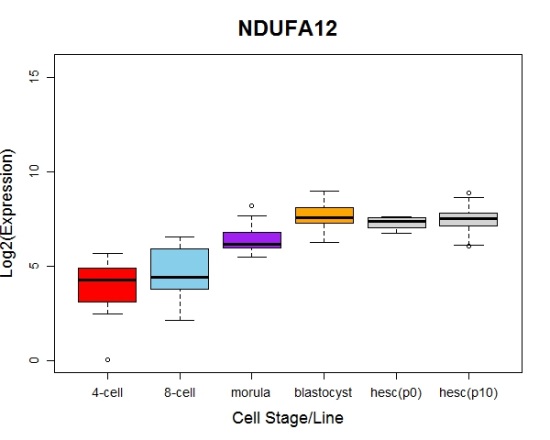

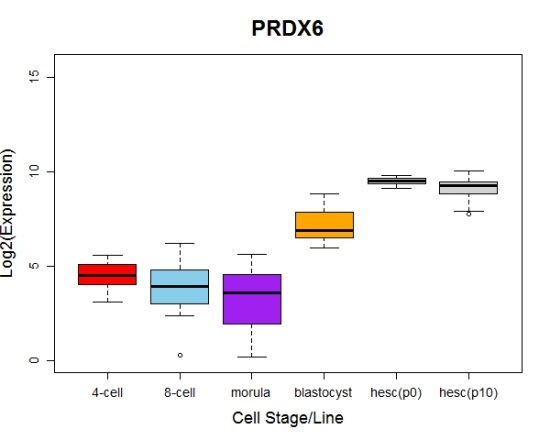

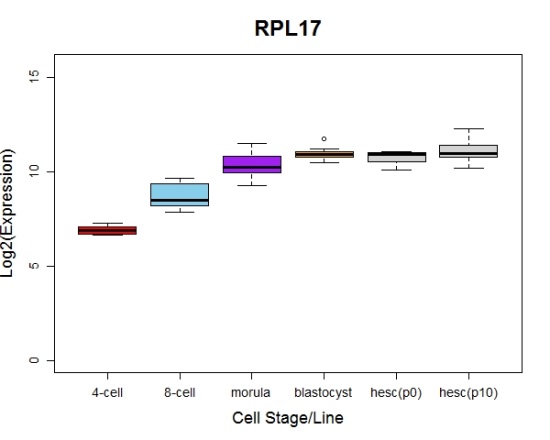

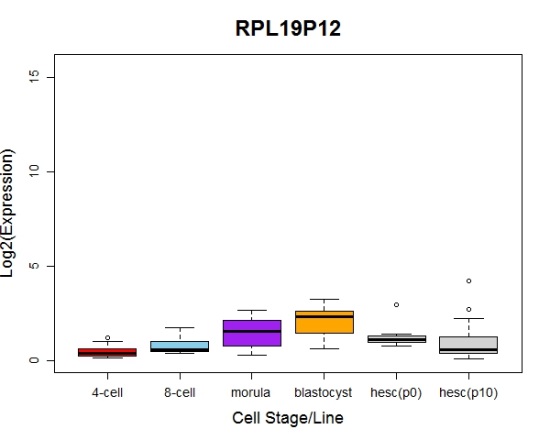

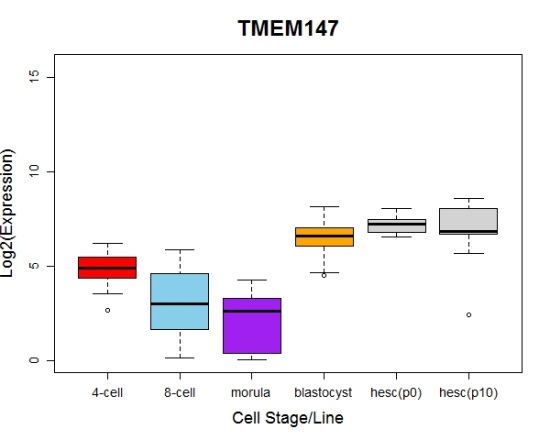

Supplement: S6 Text — (DOCX) [file pgen.1005428.s013.docx]
