# Supplementary material for: Variability of Gene Expression Identifies Transcriptional Regulators of Early Human Embryonic Development
Source: PLoS Genet. 2015 Aug 19;11(8):e1005428. doi: 10.1371/journal.pgen.1005428 (PMC4546122; doi:10.1371/journal.pgen.1005428)
Supplement: S7 Text — (DOCX) [file pgen.1005428.s014.docx]

**Text S7. Comparison of stage-specific variability markers and the genes identified using a standard ANOVA approach.**

We fitted an one-way ANOVA model using developmental stage as a factor with four levels corresponding to the 4-cell, 8-cell, morula and blastocyst stages. Even after adjustment for multiple correction using the Benjamini-Hochberg method, for standard cut-offs of significance, e.g. P-value < 0.05 or 0.01, we saw that the majority of the transcriptome showed a significant change in expression across the four developmental stages. In contrast, when we compared the number of significant genes observed from Levene’s test, the percentage of significant genes were much smaller (see Table 1). Figure 1 also demonstrated how most of the adjusted P-values are shifted towards very small values, and 86% of genes have a P-value < 0.05. Therefore in order to have a more appropriate comparison between the variability markers identified and those genes identified by a standard ANOVA approach, we cannot simply use the same significance cut-off thresholds that were applied to Levene’s test (P-value < 0.05) to the P-values from the ANOVA.

**Table 1.** The number of genes that pass cut-off thresholds for the ANOVA and Levene’s test out of a total of 8105 genes.

| **Significance Level** | **Adjusted P-value < 0.05** | **Adjusted P-value < 0.01** |
| --- | --- | --- |
| *Number of Significant Genes from ANOVA* | 6968 | 6314 |
| *Number of Significant Genes from Levene’s Test* | 1421 | 501 |

**Figure 1.** Distribution of adjusted P-values resulting from the ANOVA and Levene’s test.


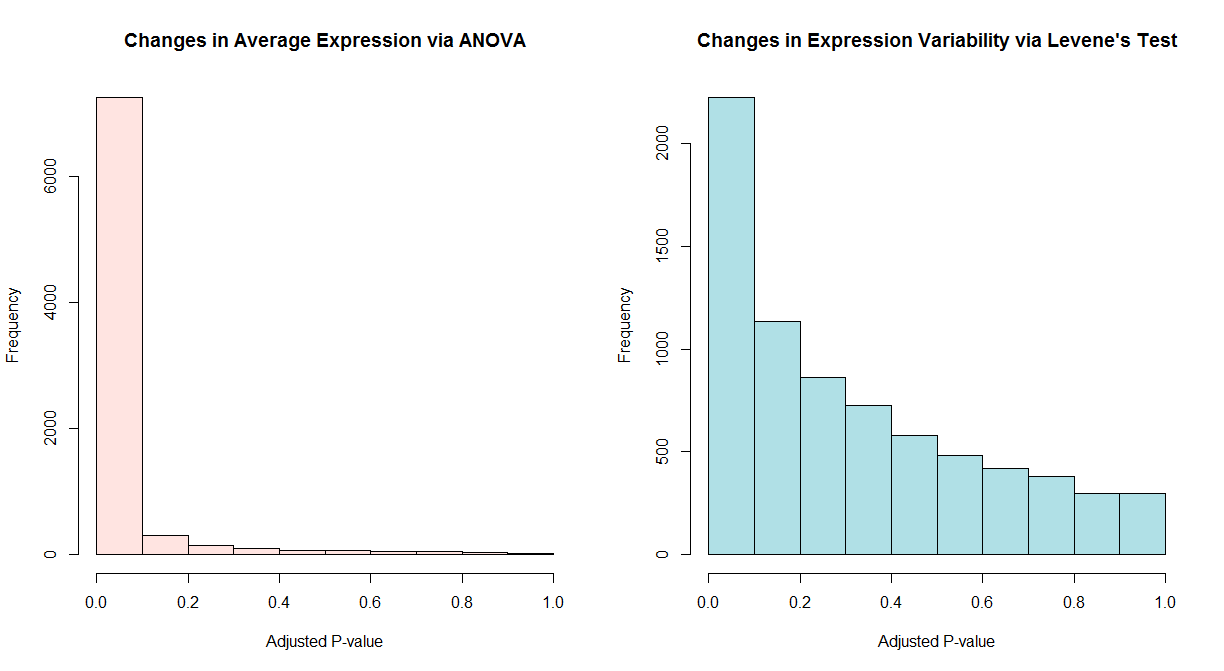


Instead, we took three different cut-offs based off of percentiles of the ANOVA adjusted P-values and compared the list of genes that satisfied these thresholds with the variability markers that were identified. The results of this comparison appear in Table 2 below, with the specific genes identified in Table 3.

**Table 2.** Overlap of genes identified by the ANOVA approach at three different cut-off thresholds with the variability markers identified for each developmental stage.

| **Overlap of Variability Markers Identified with ANOVA** | **Lower 1^th^ Percentile of ANOVA P-values**  **(P-value < 3.206×10^-24^)** | **Lower 5^th^ Percentile of ANOVA P-values**  **(P-value < 7.364×10^-18^)** | **Lower 10^th^ Percentile of ANOVA P-values**  **(P-value < 1.315×10^-14^)** | **Total Number of Variability Markers Identified** |
| --- | --- | --- | --- | --- |
| 8-cell | 0 | 0 | 5 | 55 |
| Morula | 0 | 1 | 1 | 8 |
| Blastocyst | 2 | 4 | 8 | 11 |

**Table 3.** Genes that were identified as both a stage-specific marker and by the standard ANOVA model.

| **Overlap of Variability Markers Identified with ANOVA** | **Lower 1^th^ Percentile of ANOVA P-values** | **Lower 5^th^ Percentile of ANOVA P-values** | **Lower 10^th^ Percentile of ANOVA P-values** | **Total Number of Variability Markers Identified** |
| --- | --- | --- | --- | --- |
| 8-cell | - | - | DIS3, FAM122C, MRPL16, REST, SNAPC1 | 55 |
| Morula | - | SNF8 | SNF8 | 8 |
| Blastocyst | EPCAM, PRDX6 | ACTN4, CYP2S1, EPCAM, PRDX6,  TMEM147 | ACTN4, CYP2S1, EPCAM, GSTP1,  HDDC2, NDUFA12,  PRDX6, RPL19P12,  TMEM147 | 11 |
