# Supplementary material for: Variability of Gene Expression Identifies Transcriptional Regulators of Early Human Embryonic Development
Source: PLoS Genet. 2015 Aug 19;11(8):e1005428. doi: 10.1371/journal.pgen.1005428 (PMC4546122; doi:10.1371/journal.pgen.1005428)
Supplement: S8 Text — (DOCX) [file pgen.1005428.s015.docx]

**Text S8. Validation of key results using two single cell mouse embryo data sets.**

One limitation of the Yan et al. data set is that it contained a relatively small number of embryos and cells. While there are many challenges that prevent the collection of single cell data sets with larger sample sizes for preimplantation human embryos, it has been relatively easier to collect such data sets in mice. We identified two published single cell data sets that profile similar early developmental stages for mouse embryos, and applied the same analysis of expression variability conducted on the human embryo Yan data set to these two mouse studies. We compared the results from this meta-analysis to those derived from the human data set, and used this as another means to interrogate the validity of our key findings which were encapsulated in **Fig 2** of the paper.

- A subset of genes have stable (invariant) expression across development, and these fall into three categories of low, medium and high levels of absolute expression.
- A subset of genes are more homogeneously expressed for a specific developmental stage such that they may have utility as stage-specific markers.
- The distribution of global gene expression variability widens as embryos develop, and we see genes adopt a larger range of expression variability states.

**Data Set 1: Guo et al. (2010) Cell. Resolution of Cell Fate Decisions Revealed by Single-Cell Gene Expression Analysis from Zygote to Blastocyst.**

Guo et al. profiled single cells using the Fluidigm BioMark System 48.48 Dynamic Arrays at seven stages of embryonic development in mice. The stages that were profiled were 1-cell, 2-cell, 4-cell, 8-cell, 16-cell, 32-cell and 64-cell stages. A total of 48 genes representing transcription factors were selected and their transcriptional levels were captured in single cells.

*Experimental design of the Guo data set.*

**Table 1.** The experimental design used by Guo et al. paper to profile single cell transcription for 48 genes across five developmental stages.

| Guo et al. (2010) | 4-cell | 8-cell | 16-cell | 32-cell | 64-cell |
| --- | --- | --- | --- | --- | --- |
| Number of embryos | 6 | 6 | 5 | 8 | 7 |
| Number of cells per embryo collected | 4 cells (embryo 1)  3 cells (embryo 2)  4 cells (embryo 3)  4 cells (embryo 4)  4 cells (embryo 5)  4 cells (embryo 6) | 5 cells (embryo 1)  8 cells (embryo 2)  8 cells (embryo 3)  7 cells (embryo 4)  8 cells (embryo 5)  8 cells (embryo 6) | 16 cells (embryo 1)  16 cells (embryo 2)  15 cells (embryo 3)  16 cells (embryo 4)  12 cells (embryo 5) | 21 cells (embryo 1)  16 cells (embryo 2)  8 cells (embryo 3)  23 cells (embryo 4)  23 cells (embryo 5)  8 cells (embryo 6)  8 cells (embryo 7)  6 cells (embryo 8) | 48 cells (embryo 1)  25 cells (embryo 2)  8 cells (embryo 3)  40 cells (embryo 4)  16 cells (embryo 5)  8 cells (embryo 6)  14 cells (embryo 7) |
| Total number of genes | 48 | 48 | 48 | 48 | 48 |
| Total number of cells | 23 | 44 | 75 | 113 | 159 |

*Selection of developmental stages and justification for their human stage equivalent*

To compare with our results from the human embryo data, we selected the 4-cell, 8-cell, 16-cell, 32-cell and 64-cell stages from the Guo mouse data set. These five stages are generally thought to overlap with the four human embryonic development stages that we had selected. Although it remains a rough approximation, the 32-cell stage in mouse can be thought of as a proxy for studying the morula stage in human and similarly, the 64-cell stage in mouse for late blastocyst human stage.

*Pre-processing steps of the Guo data set.*

No normalization or pre-processing was required as the normalized expression data was obtained directly from the authors by request. We calculated the inter-cellular gene expression variability, SDC as described in the Materials and Methods section for the 48 genes at each developmental stage.

*Identifying stable genes based on invariant patterns of gene expression across developmental stages.*

We identified 12 out of the 48 genes with no significant change in variability across the five developmental stages using Levene’s test (Benjamini-Hochberg adjusted P-value > 0.05). These 12 genes were: Cebpa, Eomes, Gata4, Hand1, Krt8, Lcp1, Mbnl3, Msc, Msx2, Snail, Sox13, Tcf23. We clustered the SDC profiles for these 12 genes to determine whether a stable subset of genes could be observed. Agglomerative hierarchical cluster analysis based on an Euclidean distance was used to construct the clustering. From **Fig. 1**, it was apparent that with exception of Gata4 and Cebpa, the remaining 10 genes had low levels of inter-cellular variability that remained consistent across the five developmental stages.

**Figure 1.** Heatmap of the SDC profiles show that these the majority of these 12 genes have low levels of expression variability across all five developmental stages.


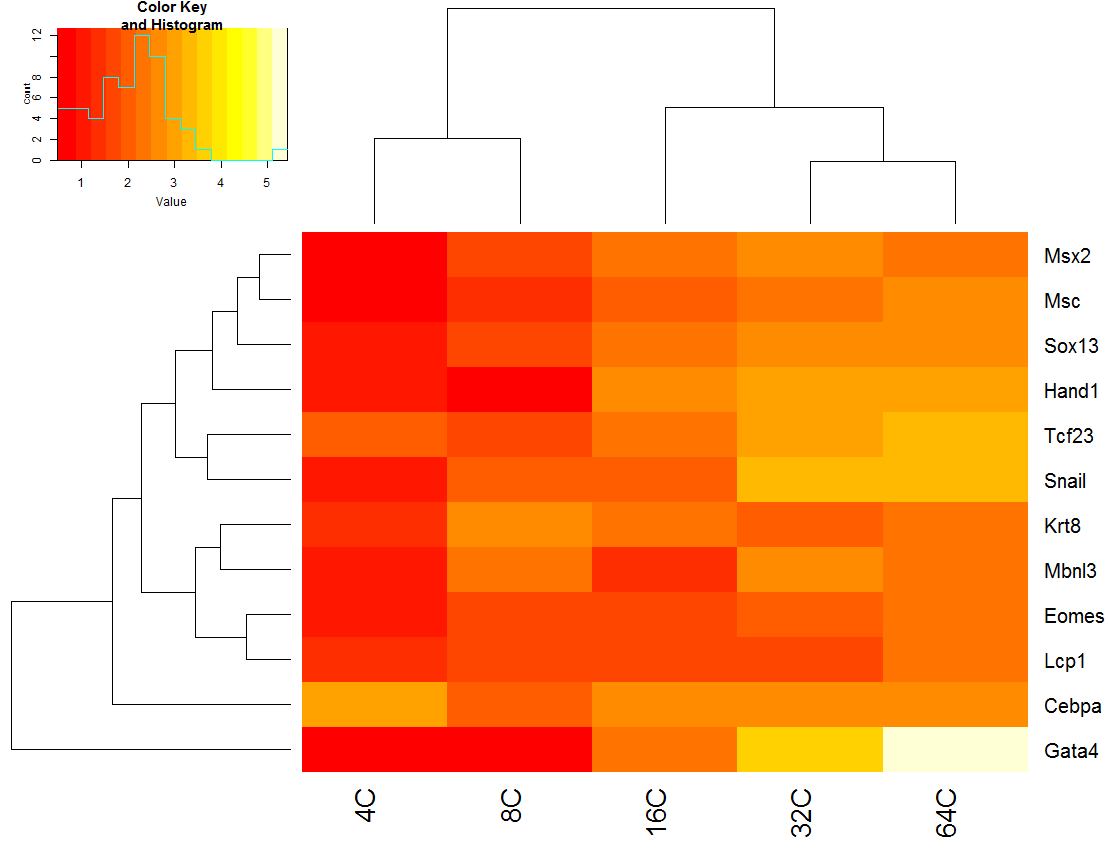


Further inspection of the individual gene expression variability profiles showed that Gata4 and Cebpa were quite different from the other ten genes with respect to variability across development. Cebpa showed sustained levels of medium expression variability across all stages except for lower variability at the 8-cell stage. Out of the 12 genes identified as not significant from Levene’s test, Gata4 was the only gene that showed consistent increasing expression variability across development, starting from very low levels at the 4-cell stage to very high levels by the 64-cell stage (see **Fig. 2**).

Gata4 is a well-known early marker of the primitive endoderm (PE), one of the three cell types that make up the inner cell membrane of the embryo. As a PE marker, the activation of Gata4 is restricted to only a subpopulation of cells and hence

**Figure 2.** Gene expression variability profiles for each individual gene. Each point in the plot represents the SDC measured at a developmental stage.


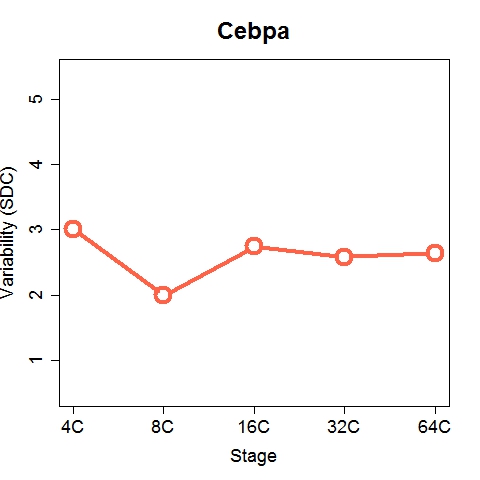

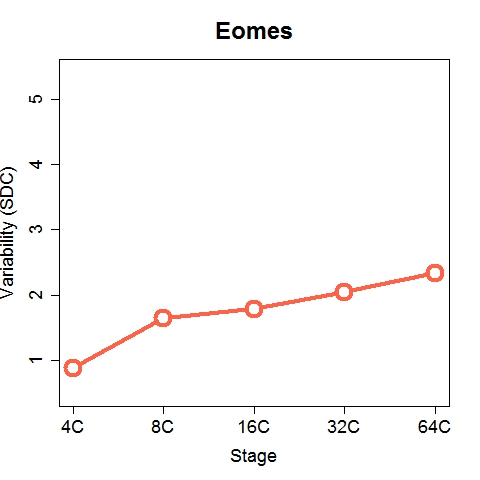

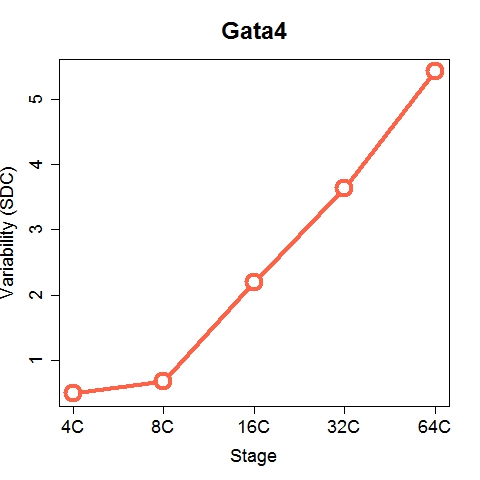

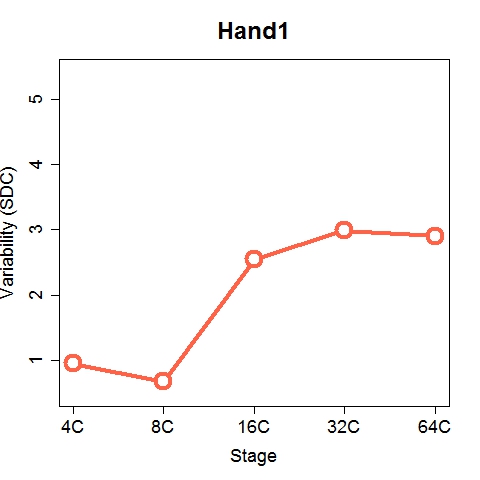

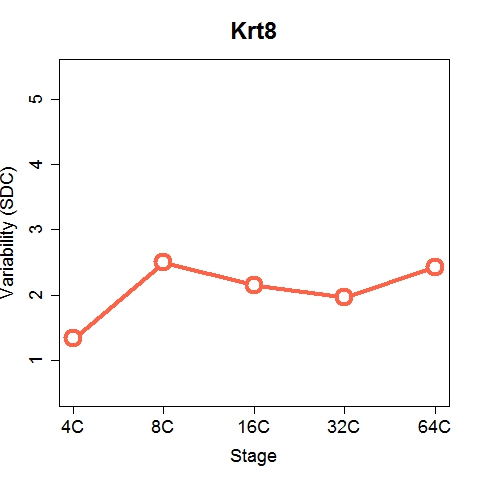

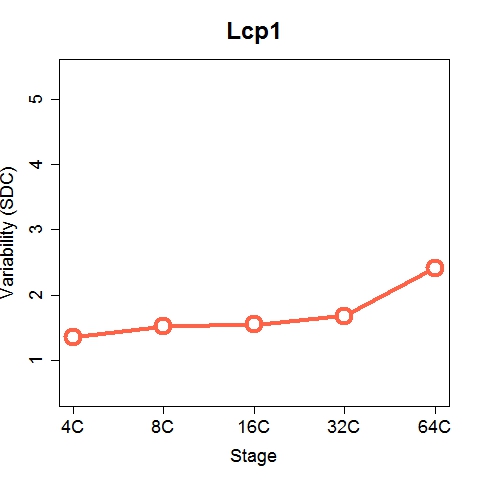

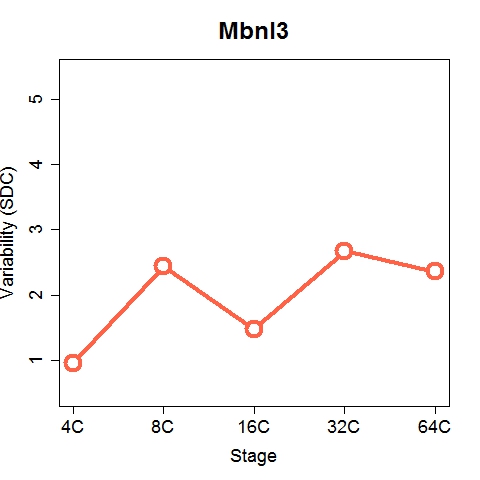

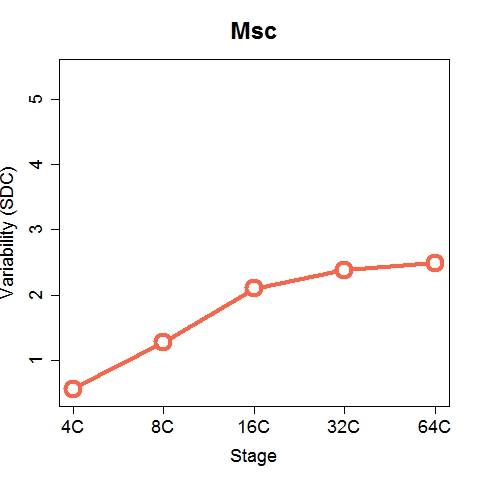

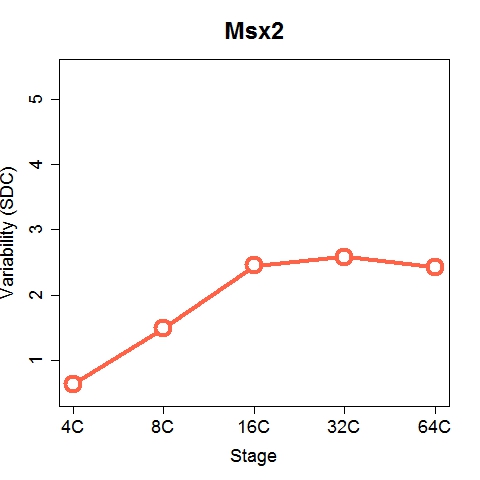


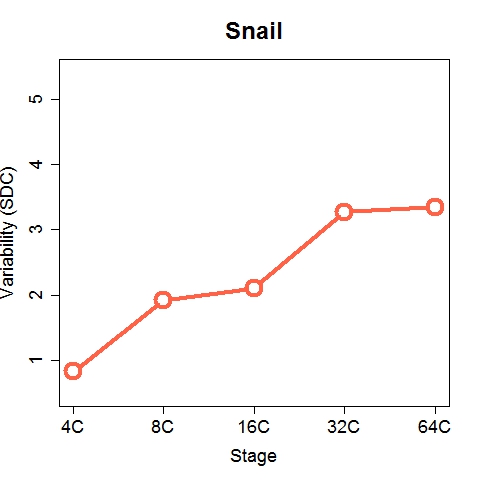

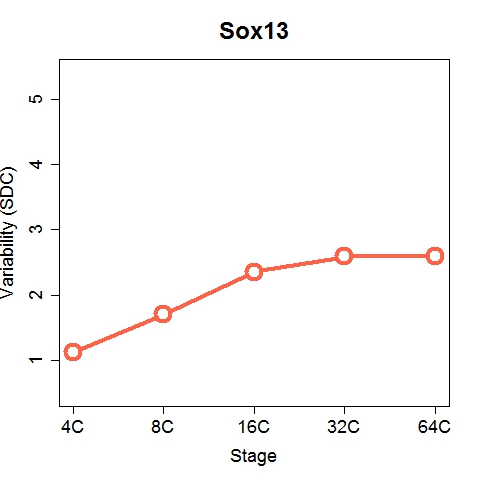

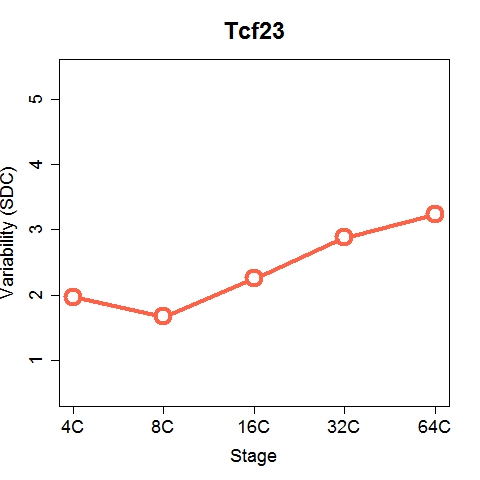


the increased expression variability of Gata4 at the 64-cell stage is likely to reflect this specificity of cell type expression since the blastocyst consists of at least three cell types, the trophectoderm, the epiblast and the PE. Moreover, Guo et al. reported that Gata4 was enriched in only forty cells (25%) that were collected from the 64-cell stage blastocyst. Boxplots of the expression levels of Gata4 in the cell population during development displayed in Fig. 6B of the original paper highlighted how the expression of Gata4 becomes more segregated into specific states as the embryo develops, and therefore giving rise to more variable expression across the cell population of a specific stage.

The dendrogram of the remaining 10 genes, coupled with the individual expression variability profiles showed that this subset of genes had very low to moderate levels of variability across development and therefore we designated this set as the stable genes.

**Table 2.** List of stable and variable genes determined by Levene’s test and hierarchical cluster analysis.

| **Stable Genes** | **Variable Genes** | |
| --- | --- | --- |
|  | Non-Significant Levene’s test (adjusted P-value > 0.5) | Significant Levene’s test  (adjusted P-value < 0.05) |
| Eomes, Hand1, Krt8,  Lcp1, Mbnl3, Msc,  Msx2, Snail, Sox13,  Tcf23 | Cebpa,  Gata4 | Actb, Ahcy, Aqp3, Atp12a, Bmp4, Cdx2, Creb312, Dab2, DppaI, Esrrb, Fgf4, Fgfr2, Fn1, Gapdh, Gata3, Gata6, Grhl1, Grhl2, Hnf4a, Id2, Klf2, Klf4, Klf5, Nanog, Pdgfa, Pdgfra, Pecam1, Pou5f1, Runx1, Sox2, Sall4, Sox17, Tcfap2a,  Tcfap2c, Utf1, Tspan8 |
| **10 genes** | **2 genes** | **36 genes** |

The 48 mouse genes were mapped to their available human orthologs using the homology file **HOM_MouseHumanSequence.rpt** that was downloaded from the Mouse Genome Informatics database. We calculated the overlap between the stable and variable genes identified from the Guo mouse data set, with those from the human study. For the stable genes, there was no overlap detected however for the variable genes, there were 10 genes in common between human and mouse (see Table. 3).

**Table 3.** Overlap between the lists of stable and variable genes identified in human and Guo mouse embryo studies.

| ***Overlapping Gene Counts (Guo)*** | *Mouse Stable Genes* | *Mouse Variable Genes* |
| --- | --- | --- |
| *Human Stable Genes* | **0 genes** | **1 gene** (AHCY) |
| *Human Variable Genes* | **2 genes**  (KRT8, SOX13) | **10 genes**  (ACTB, FGFR2, FN1, ID2, KLF4, KLF5, NANOG, PDGFA, POU5F1, SALL4) |

It is worth noting that for the 48 mouse genes that were profiled in the Guo data set, it was only possible to map 43 mouse genes to a known human orthologs. From this list of 43 mouse genes, only 13 mouse genes had human orthologs that overlapped with the 8105 human genes that were used in analysis of the Yan data (genes were filtered out if they did not meet a quality threshold, see Text S1). Therefore in assessing the overlap between results from mouse and human studies, we are really starting from a comparison based on only 13 genes. Given the large discrepancy between the resolution of the platforms used in these two studies – genome-wide in human versus a 48-gene panel in mouse – it does not seem very fruitful to compare the overlap as evidence for validation.

*Using gene expression variability to identify stage-specific variability markers.*

We applied the same criteria to screen for stage-specific variability markers in the Guo data set. To qualify as a marker, a gene had to have:

1. A statistically significant change in variability as assessed by Levene’s test (adjusted P-value < 0.05).
2. The lowest expression variability compared to all other stages.
3. The highest average expression level compared to all other stages.

We detected a subset of genes satisfying all three criteria for 4-cell, 8-cell and 16-cell stages. These genes are listed in Table 4. It is likely that no variability markers were detectable for the later developmental stages, 32-cell and 64-cell stage because many of the genes selected by Guo et al. to study are markers of different cell types of the ICM. Hence, we would expect the expression variability of these genes to increase, such as the behavior that was observed for Gata4 where only a subset of cells were expressing a specific marker. Criteria 2 and 3 therefore would not hold for cell type-specific genes, and it is therefore not surprising that we did not detect variability markers for the last two developmental stages.

**Table 4.** List of potential variability markers detected for each specific developmental stage in the Guo data set.

| **Stage** | **Number of Markers** | **Genes** |
| --- | --- | --- |
| **4-cell** | 13 | Bmp4, Esrrb, Fgf4, Fn1, Hnf4a, Klf2, Klf4, Pecam1, Sox2, Sall4, Tcfap2a, Tcfap2c, Tspan8 |
| **8-cell** | 1 | Pdgfra |
| **16-cell** | 7 | Ahcy, Aqp3, Dab2, Gata6, Klf5, Pdgfa, Utf1 |
| **32-cell** | 0 | - |
| **64-cell** | 0 | - |

The overall distribution of gene expression associated with these markers appear to follow the trends of increased average expression and decreased variability for the specific stage that they are delineating (Fig. 3).

**Figure 3.** Boxplots show the expression distribution of genes that are potential stage-specific variability markers from the Guo mouse embryo data set.


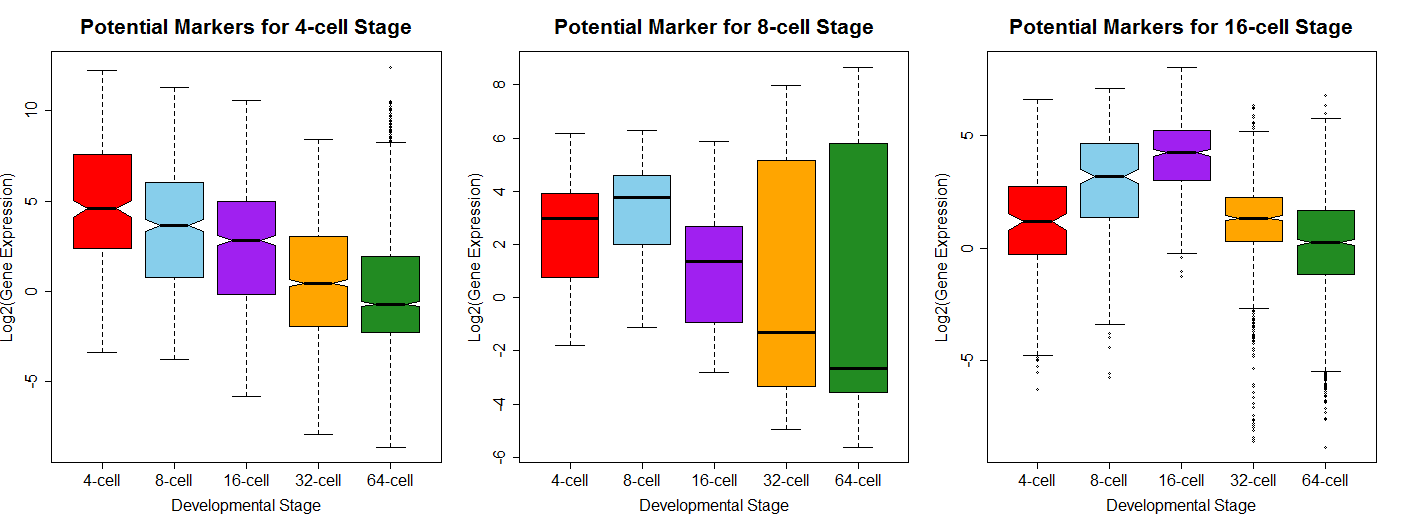


Inspection of the single cell gene expression profiles for each individual marker highlight the distributional changes across different developmental stages (see Figs. 4-6).

**Figure 4.** Single cell gene expression profiles for the 4-cell variability markers. Each circle represents the expression level of the gene in a cell. The colored circles represent the stage-specific average expression where expression has been averaged over all cells.


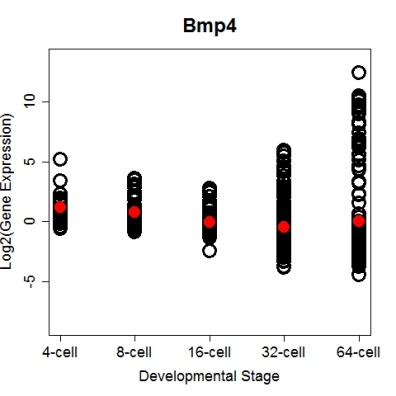

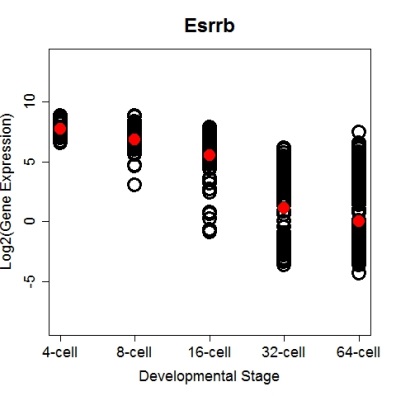

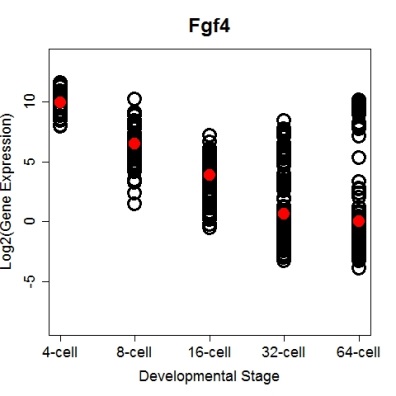

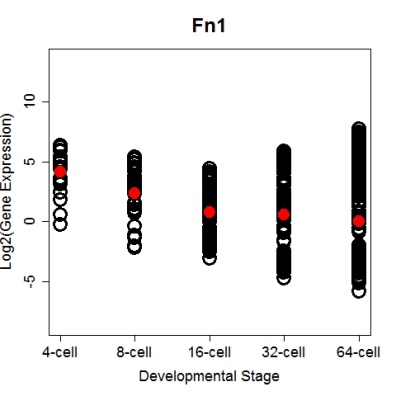

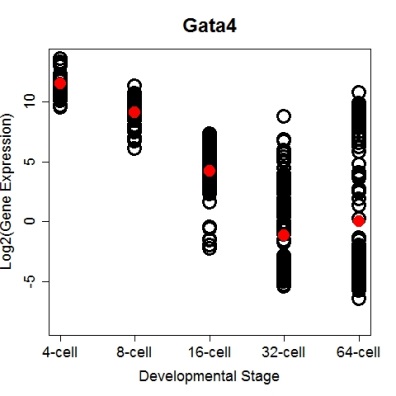

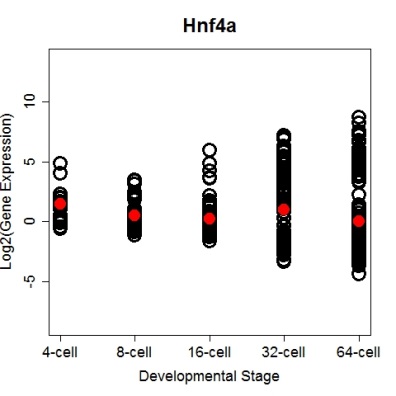

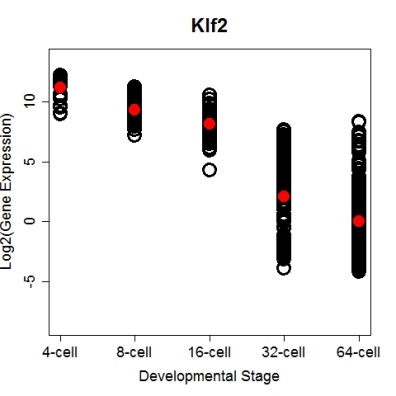

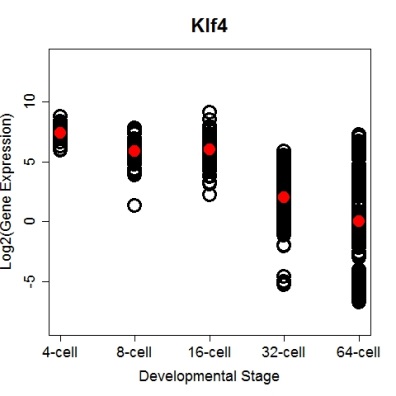

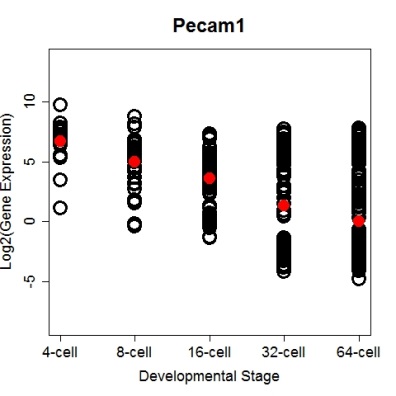

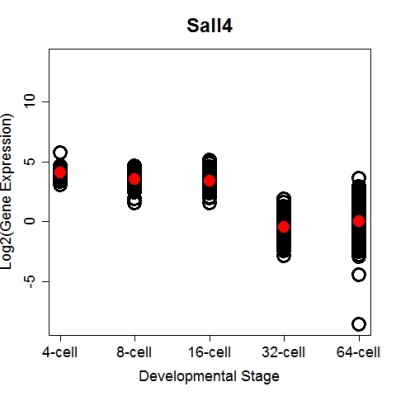

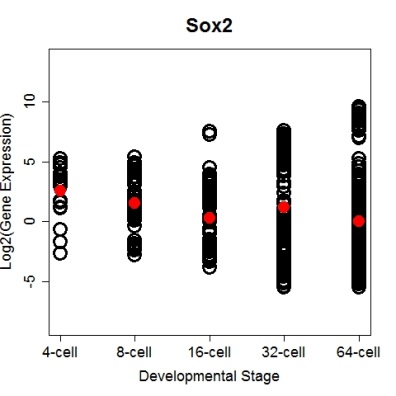

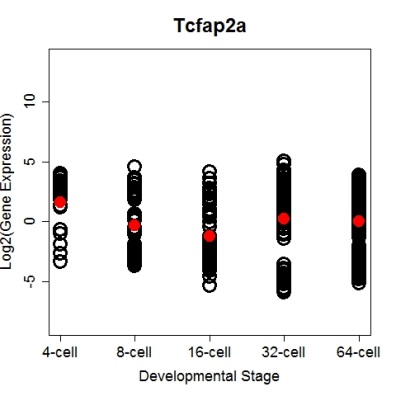

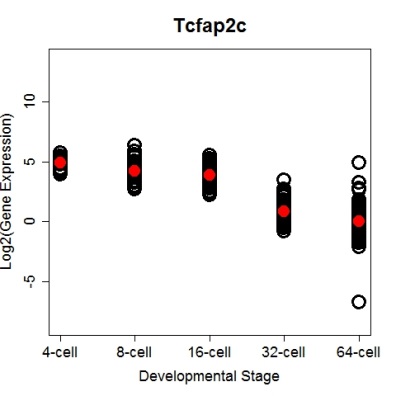

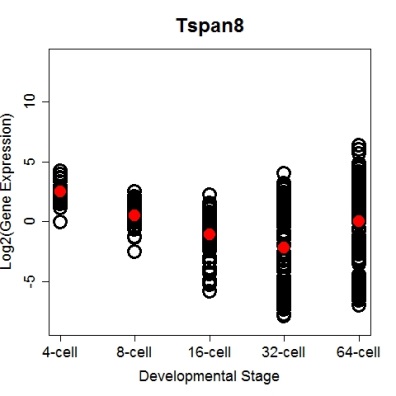


**Figure 5.** Single cell gene expression profile for the 8-cell variability marker, Pdgfra. Each circle represents the expression level of the gene in a cell. The colored circles represent the stage-specific average expression where expression has been averaged over all cells.


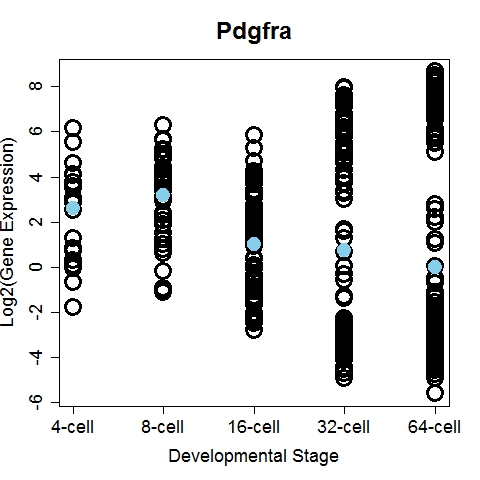


**Figure 6.** Single cell gene expression profiles for the 16-cell variability markers. Each circle represents the expression level of the gene in a cell. The colored circles represent the stage-specific average expression where expression has been averaged over all cells.


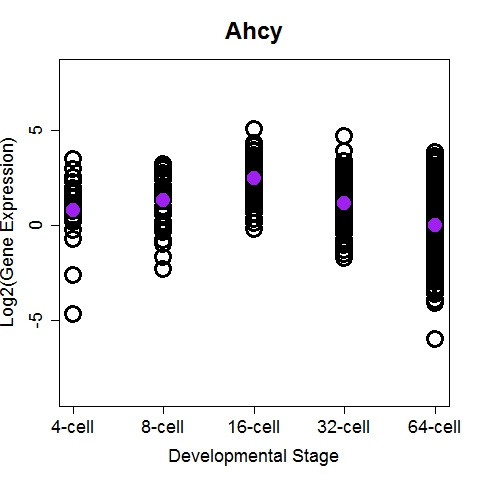

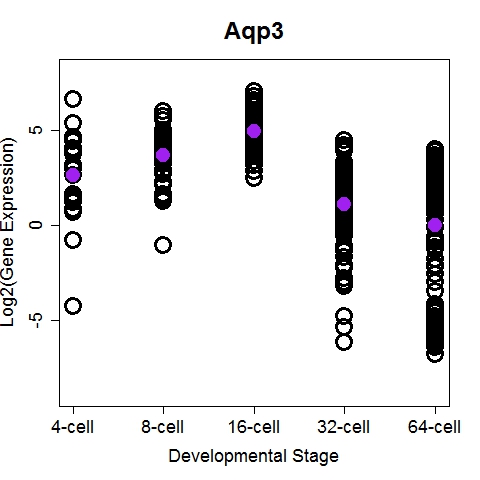

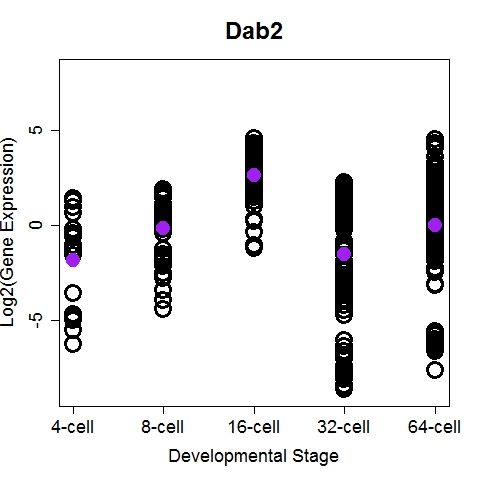
A
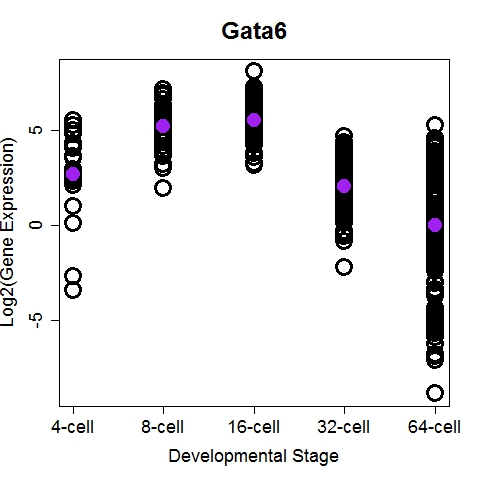

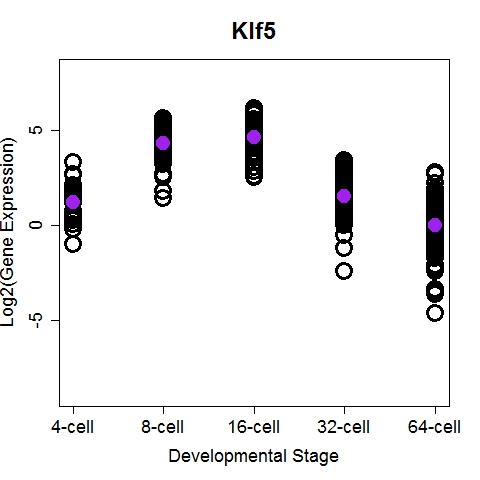

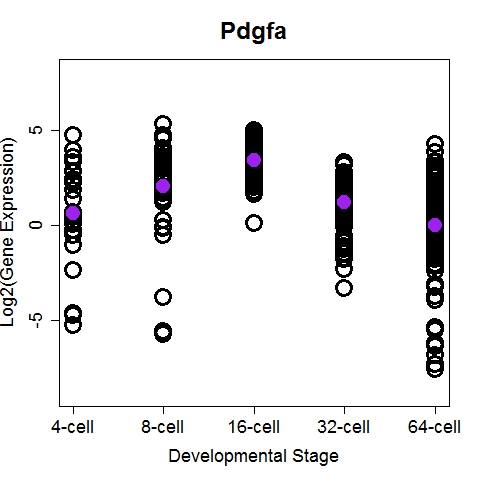

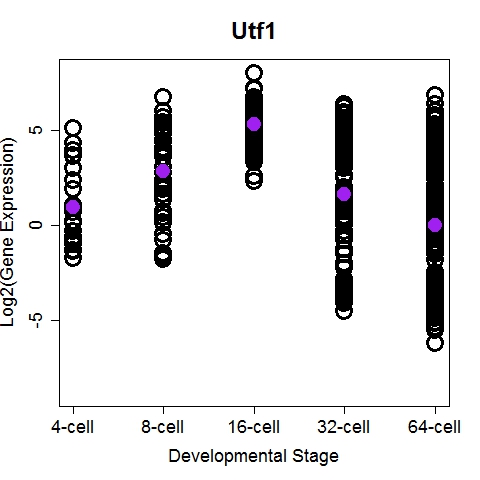


*The global distribution of intercellular gene expression variability also widens during mouse embryo development.*

Although in the Guo data set, we only had transcriptional profiles available for 48 genes, the violin plots demonstrated that this “global” distribution of intercellular expression variability followed an increasing trend in a similar way as was observed for the human data. We also see the inter-embryo expression variability, as measured by the SDE, remained relatively constant across all developmental stages in the mouse data (Fig. 7). While we have a much fewer number of genes in this data set, it is worthwhile to emphasize that due to the higher number of cells available, our estimates of the inter-cellular variability are much more stable and accurate. Therefore, it is interesting to note that the same trends in the distribution of SDC that were observed for the human data are also apparent in the mouse data.

**Figure 7.** Violin plots show that the distribution of inter-cellular expression variability adopts a wider range of values as mouse embryos develop. The distribution of inter-embryo variability on the other hand remains relatively constant.


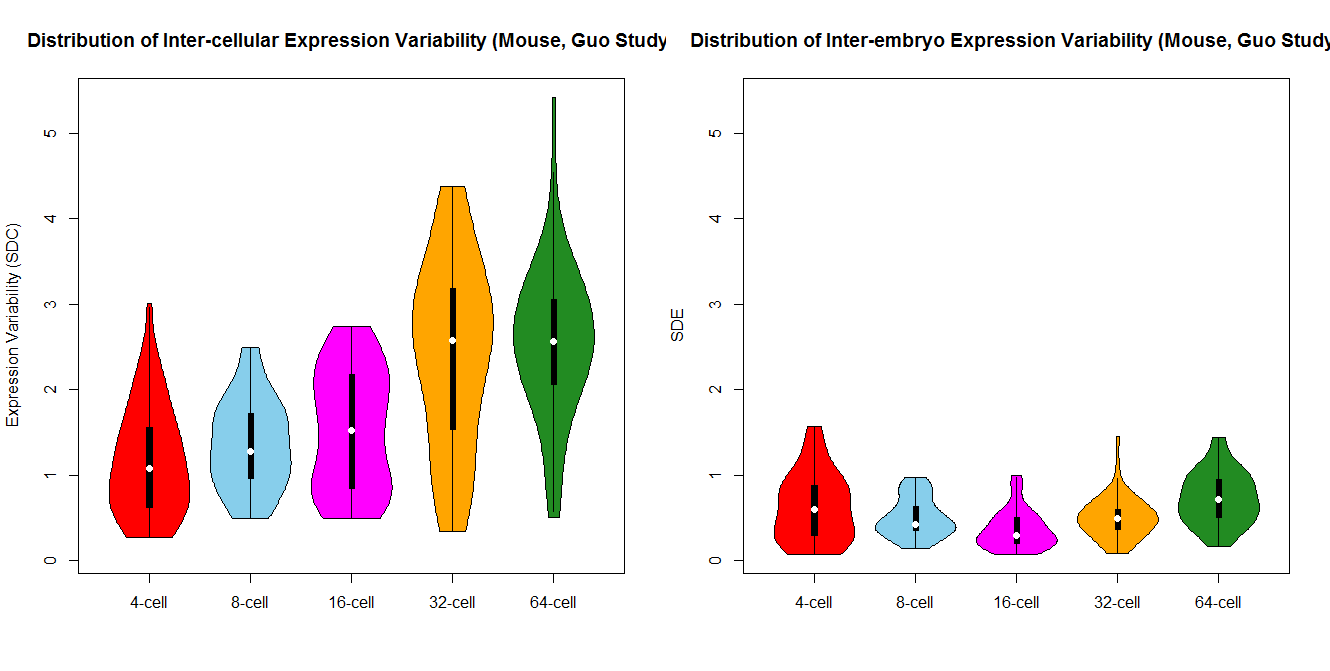


*Conclusion*

The application of our expression variability analysis to the Guo data set demonstrated that we were able to verify all three of the key findings from the human embryo data set. Similar to the Yan data set, we observed a subset of genes with stable expression across development in the mouse embryos. While we did not see an overlap in actual genes, this comparison was hindered due to the fact that we had such different numbers of genes to work with. This factor was the motivation to look for a second mouse study that featured a more comparable number of transcripts so that we can better validate our results using a more appropriate data set.

**Data set 2: Deng et al. (2014). Science. Single-Cell RNA-Seq Reveals Dynamics, Random Monoallelic Gene Expression in Mammalian Cells**

Deng et al. used Smart-seq and Smart-seq2 single cell RNA-sequencing to profile the transcriptomes of 269 individual cells extracted from mouse embryos at early developmental stages ranging from zygote to the late blastocyst, and including the oocyte. Their primary purpose was to understand how monoallelic gene expression is controlled in preimplantation embryos.

*Experimental design of the Deng dataset.*

**Table 5.** The experimental design used by Deng et al. paper to profile the single cell transcriptome across the four developmental stages selected for validation of our results from the human study.

| **Deng et al. (2014)** | *4-cell* | *8-cell* | *16-cell* | *Late blastocyst* |
| --- | --- | --- | --- | --- |
| **Number of Embryos** | 4 | 6 | 4 | 2 |
| **Number of cells per embryo collected** | 3 cells (embryo 1)  4 cells (embryo 2)  3 cells (embryo 3)  4 cells (embryo 4) | 7 cells (embryo 1)  4 cells (embryo 2)  4 cells (embryo 3)  7 cells (embryo 4)  7 cells (embryo 5)  7 cells (embryo 6) | 14 cells (embryo 1)  11 cells (embryo 2)  13 cells (embryo 3)  12 cells (embryo 4) | 19 cells (embryo 1)  11 cells (embryo 2) |
| **Number of genes (after filtering for quality control)** | 5439 | 5439 | 5439 | 5439 |
| **Total number of cells** | 14 | 36 | 50 | 30 |

*Selection of developmental stages and justification for their human stage equivalent.*

For the purpose of validating our original analysis, we took the following cellular stages: 4-cell, 8-cell, 16-cell and late blastocyst stages to compare with the results obtained from the human embryo data set.

*Pre-processing steps and quality control of the Deng data set.*

We applied the same pre-processing steps the Deng data set as we did for the Yan data set since they were both generated by similar technology platforms and had similar experimental designs. Starting from the normalized gene expression data, a cut-off of RPKM ≥ 0.1 was used to filter out transcripts with low expression. The distribution of the number of cells that passed this filter criterion was similar to the distribution observed from our quality control results (see Figure 8, and Appendix S1 for the corresponding information on the Yan human embryo data set). We apply the same requirement that a transcript must be expressed (RPKM ≥ 0.1) in at least 75% of the total cell samples to be included in the analysis, this results in 5439 genes. The SDC and SDE were calculated as described in the Materials and Methods section.

**Figure 8.** Distribution of genes that have percentage of total cells passing the expression threshold RPKM ≥ 0.1.


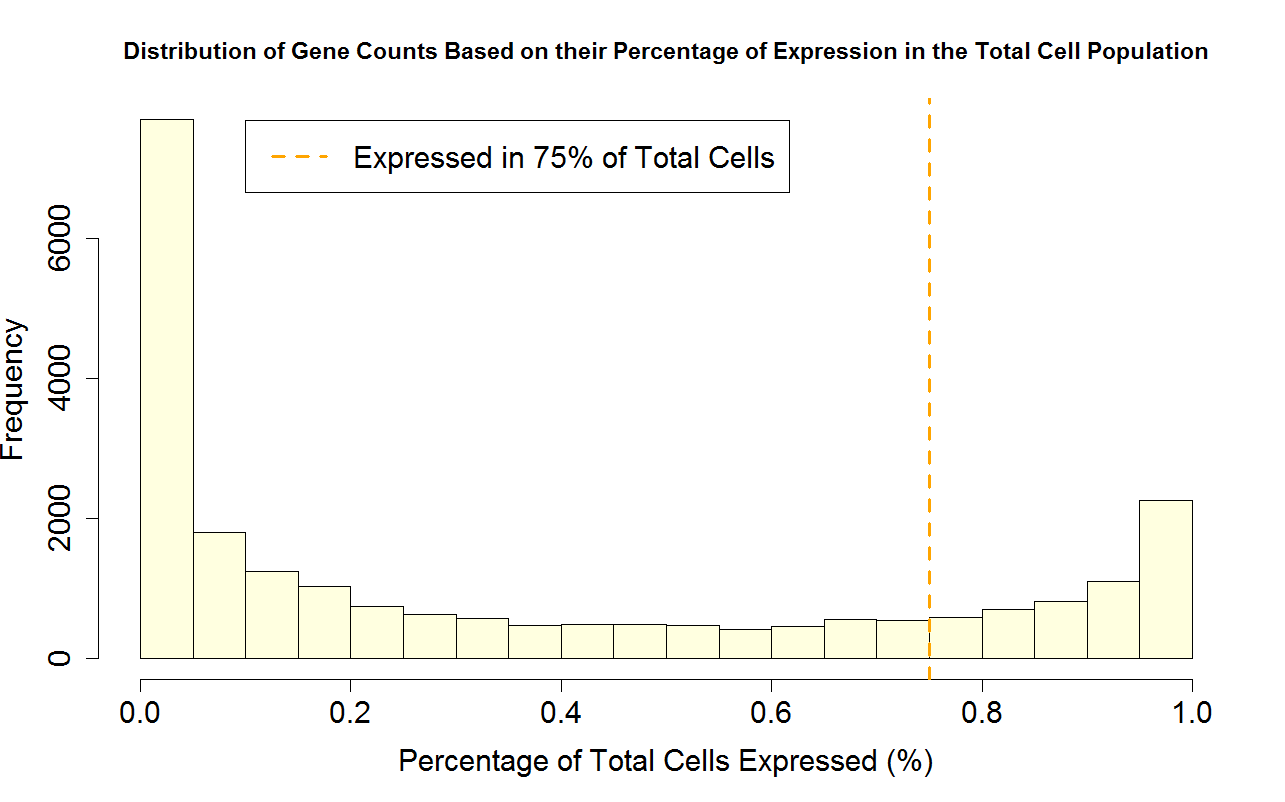


*Identifying stable genes using the Deng mouse data set.*

Levene’s test was used to identify the subset of genes that did not have a significant change in expression variability across the developmental stages (Benjamini-Hochberg adjusted P-value > 0.05). A Normal mixture model using the R package **mclust** was used to cluster the genes into three groups based on their SDC values (Fig. 9). These three groups corresponded to levels of low, medium and high variability across the five stages (Fig. 10). 912 genes were grouped into the low variability category (Cluster 1), 1620 genes in the medium variability category (Cluster 2) and 490 genes in the high variability category (Cluster 3). We defined the stable genes as those genes that were assigned to Cluster 1.

**Figure 9.** Schematic outlining the approach used to identify the stable genes from the Deng mouse embryo data set.


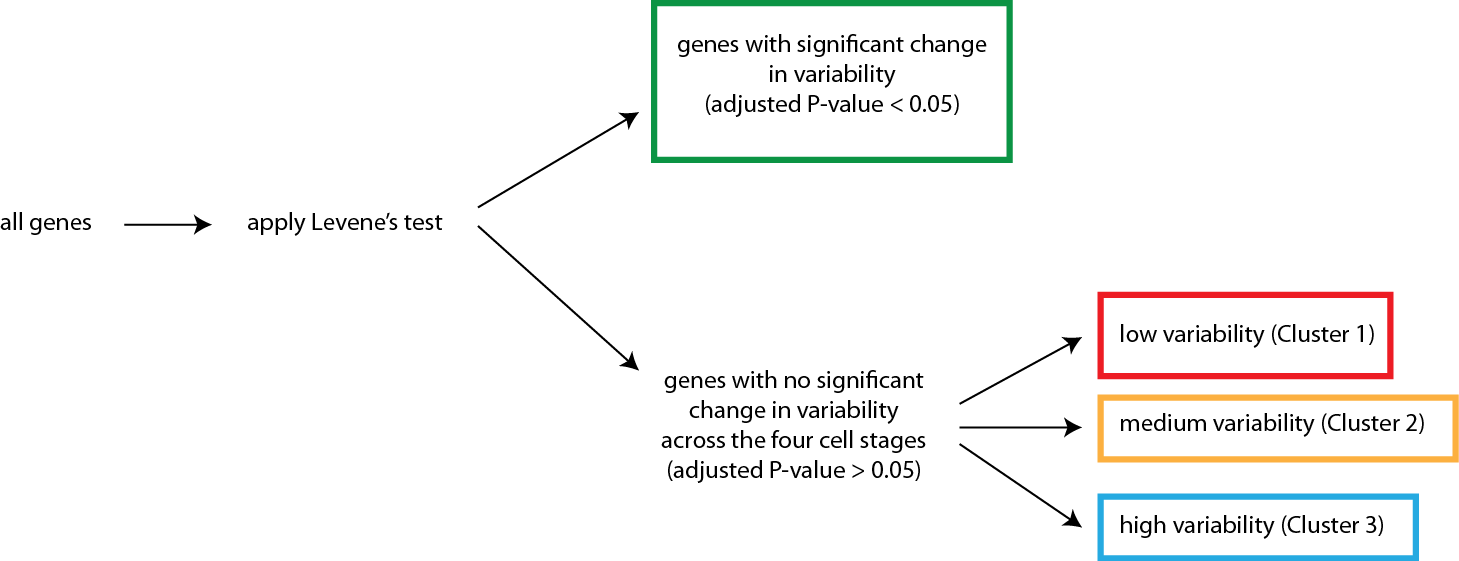


**Figure 10.** The average SDC values for each of the clusters identified from the genes with no significant change in expression variability. Of the three clusters, Cluster 1 has the most stable level of variability. Genes in Cluster 1 were designated the stable genes in the Deng mouse embryo data set.


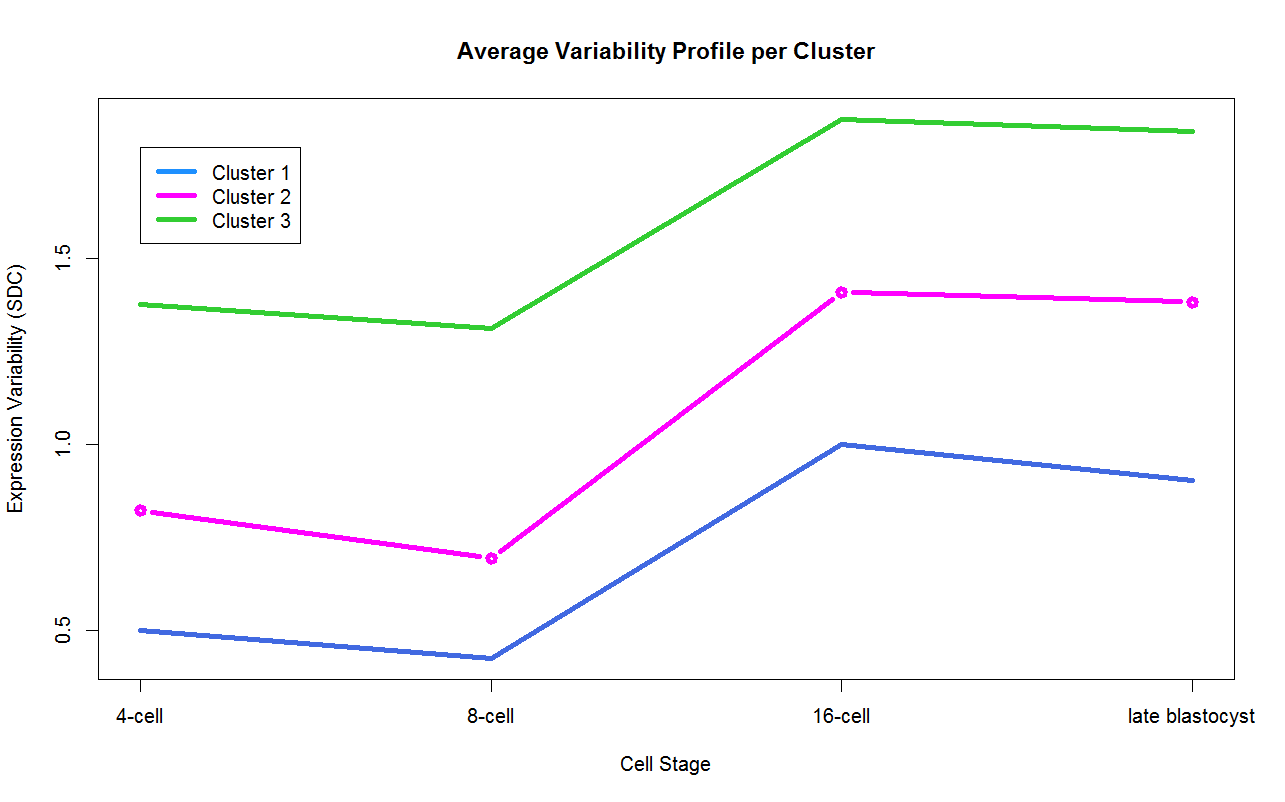


Mouse genes were mapped to their human orthologs using the homology file from the Mouse Genome Informatics database **HOM_MouseHumanSequence.rpt**. We calculated the overlap between the stable and variable genes identified in the Deng data set and the human embryo data set. 127 stable genes and 3441 variable genes were found to be in common between the two data sets. This overlap was found to be statistically significant (two-sided Fisher’s exact test, P-value = 2.5638 x 10^-24^) with an odds ratio estimate of 3.744, and a 95^th^ percent confidence interval of (2.940, 4.750).

**Table 6.** Overlap between the list of stable and variable genes identified in human and Deng mouse embryo studies.

| ***Overlapping Gene Counts (Deng)*** | *Mouse Stable Genes* | *Mouse Variable Genes* |
| --- | --- | --- |
| *Human Stable Genes* | **127** | **408** |
| *Human Variable Genes* | **286** | **3441** |
| *Total* | 413 | 3849 |

We also conducted a permutation test to evaluate the significance of the overlap between mouse and human stable genes that was observed. We randomly sampled 413 genes from the universe of 4262 genes (the number of genes that had available mouse-human orthologues) and calculated the overlap between human and mouse stable gene lists. We performed 106 permutations, and counted the number of permutations that yielded an overlap that was as or more extreme than what we observed with the real data (127 genes). No permutation satisfied this criterion (see Fig. 11.), and hence the permutation P-value was statistically significant (P-value < 10^-6^_)_.

**Figure 11.**


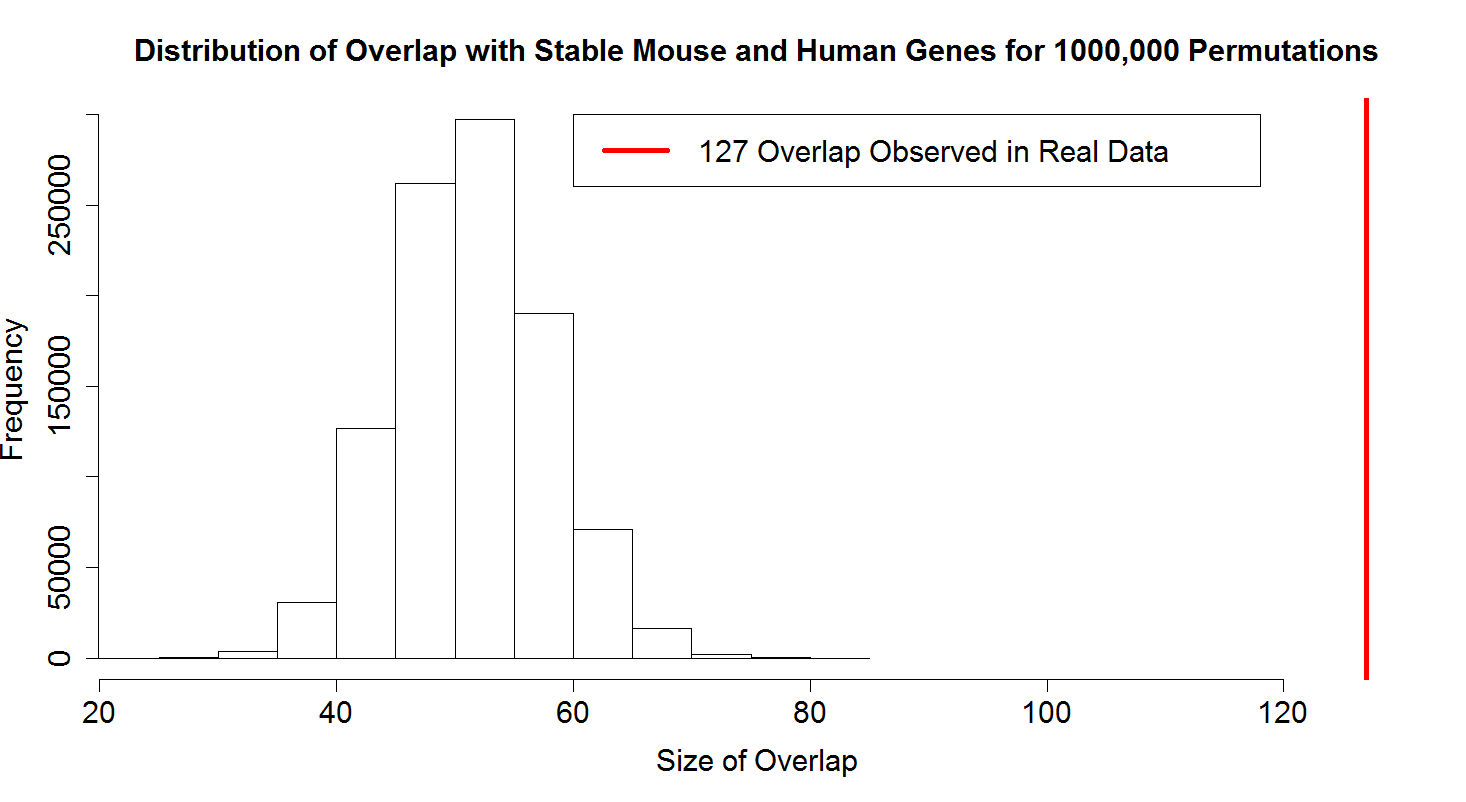


*Over-representation analysis of biological pathways in the human versus mouse comparison of stable genes.*

We performed over-representation analyses to look for functional enrichment of biological pathways to better understand the four gene lists resulting from the comparison of human versus mouse stable genes. We used the Investigate Gene Sets tool from the Gene Set Enrichment Analysis software from the Broad Institute (<http://www.broadinstitute.org/gsea/msigdb/annotate.jsp>) and used the Hallmark gene set terms (H) and computational gene set terms (C4) only in MSigDB. Statistically significant terms that had an False Discovery Rate q-value < 10^-25^ were retained.

For genes that were stable in both mouse and human embryos, the two most significant terms enriched was a list of MYC targets and housekeeping genes (see Table 7). We also saw pathways that control core metabolism (e.g. Metabolism of mRNA, RNA, Proteins) and translation.

**Table 7.** List of statistically significant terms from MSigDB for the stable genes that were in both mouse and human embryo data sets (FDR q-value < 10^-25^).

For genes that were stable in human embryos, but variable in mouse embryos (Table 8), we saw many disease-related terms e.g. Nasopharyngeal Carcinoma, Targets of Ewing’s sarcoma, Alzheimer’s Disease. Amongst the list of significant terms, there was some overlap with the terms enriched in the human-mouse stable gene list.

For genes that were stable in the mouse embryos but variable in the human embryos (Table 9), we saw the largest number of significant terms (with the same significance cut-off, q-value < 10^-25^). For this list we also saw many disease-related terms, as well as terms that were also common to the human-mouse stable gene list such as the housekeeping genes. However two terms that were unique to this set were related to pluripotency and embryonic stem cell activity, the Wong Embryonic Stem Cell Core set, and the Mueller PluriNet protein-protein network.

Based on the results of these analyses, it may be that genes that are different between mice and humans with respect to gene expression variability play a role in human-specific (or mouse-specific) diseases. They may also represent the parts of the transcriptome that are required for mouse-specific or human-specific embryonic development.

**Table 8.** List of statistically significant terms from MSigDB for genes that were stable in the human embryos but variable in the mouse embryos (FDR q-value < 10^-25^).

**Table 9.** List of statistically significant terms from MSigDB for the genes that were variable in the human embryos and stable in the mouse embryos (FDR q-value < 10^-25^).

**Table 9 (continued).** List of statistically significant terms from MSigDB for the genes that were variable in the human embryos and stable in the mouse embryos (FDR q-value < 10^-25^).

*Using gene expression variability to identify stage-specific variability markers.*

Using the same criteria that was applied to the human embryo data set, we identified sets of stage-specific variability markers in the Deng data set for each of the four developmental stages (Table 10).

**Table 10.** Number of stage-specific variability marker genes identified using the Deng mouse embryo data set.

| **Deng Data Set** | *4-cell* | *8-cell* | *16-cell* | *Late Blastocyst* |
| --- | --- | --- | --- | --- |
| **Number of Variability Markers** | 681 | 285 | 2 | 85 |

We see that there are many more markers detected from the mouse transcriptome than compared with the human embryo data set. This may be because the genetic variation is much more homogeneous in the embryos of the lab mice that were used than in human embryos, and therefore more genes satisfy the criteria we imposed to detect these variability markers.

The boxplots in Fig. 12 represent the expression values of the marker genes averaged over all cells for each stage and highlight how the expression average appears to be overall elevated for the specific stage, with decreased gene expression variability. We have omitted the distributions for the 16-cell stage markers because there were only two genes identified. To better inspect the single cell gene expression distribution profiles, these have been plotted in Figs. 13-16 for markers from the four developmental stages.

**Figure 12.** Boxplots show the expression distribution of genes that are potential stage-specific variability markers from the Deng mouse embryo data set.


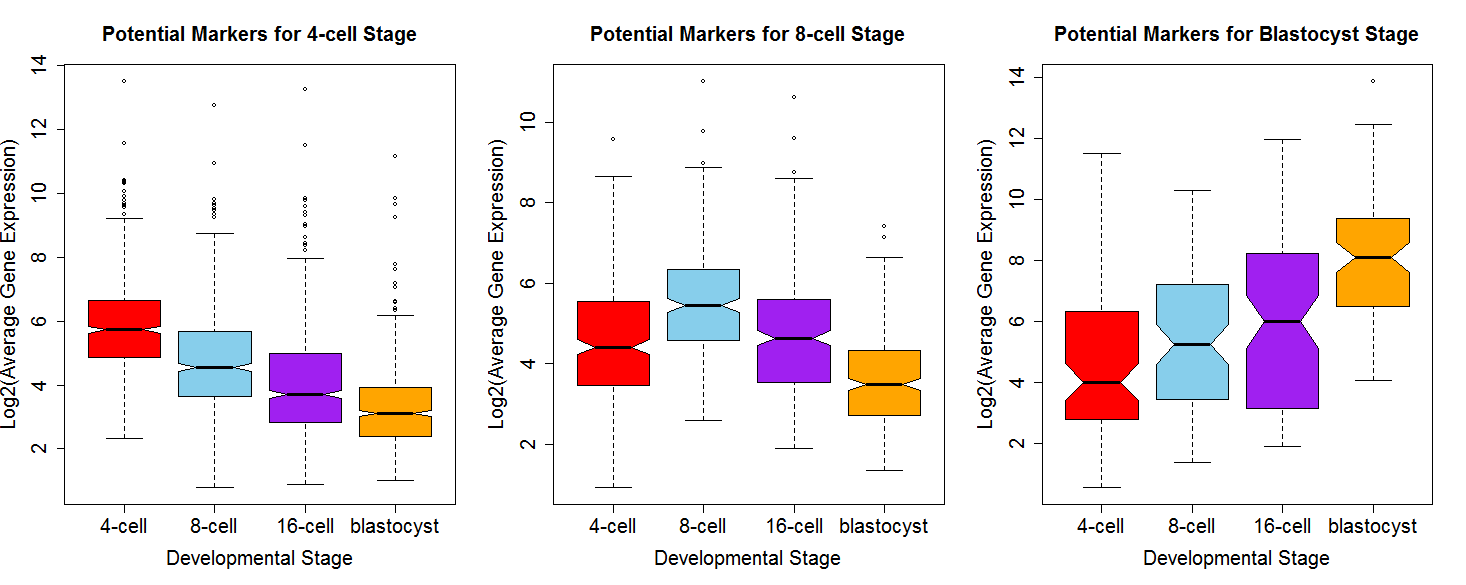


**Figure 13.** Twelve variability markers for the 4-cell stage from the Deng mouse embryo data set.


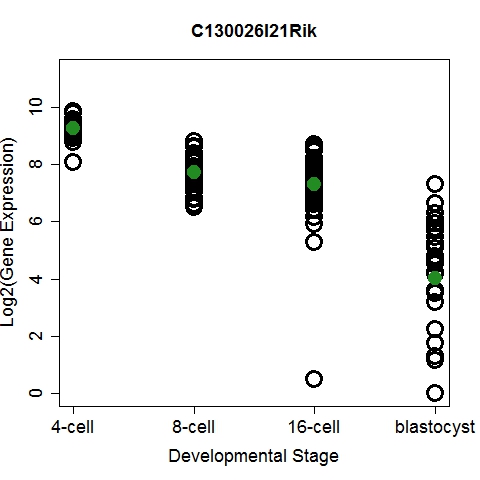

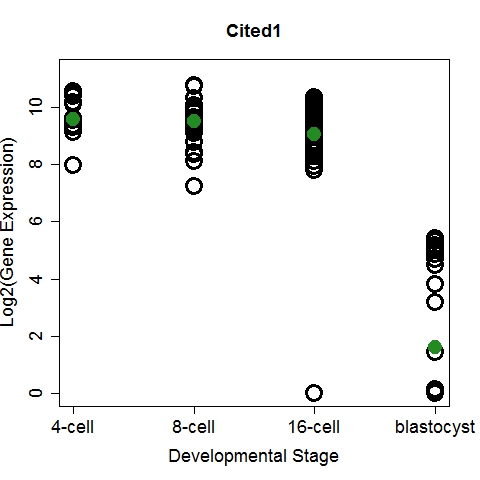

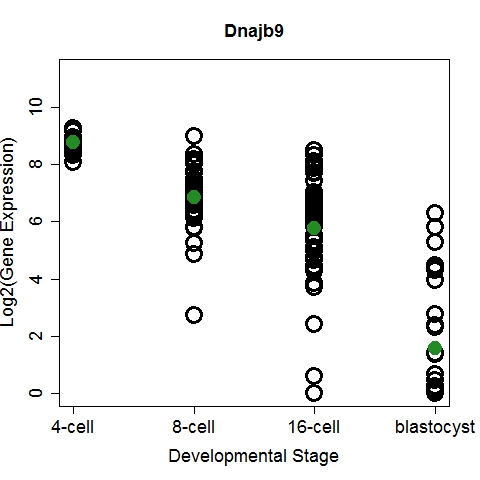

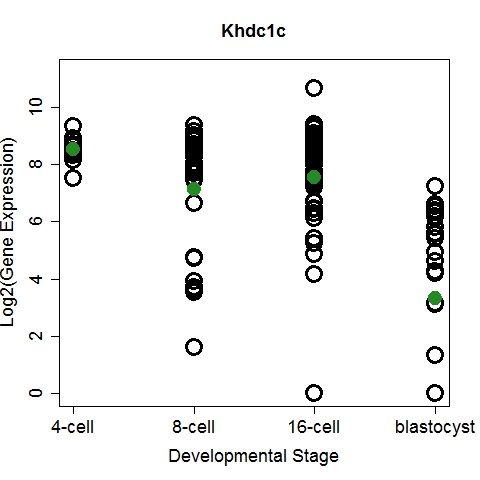

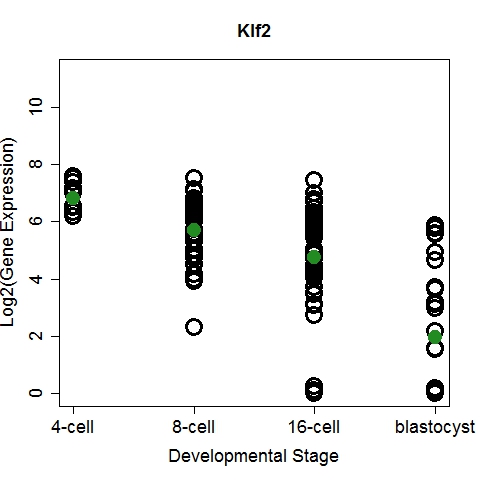

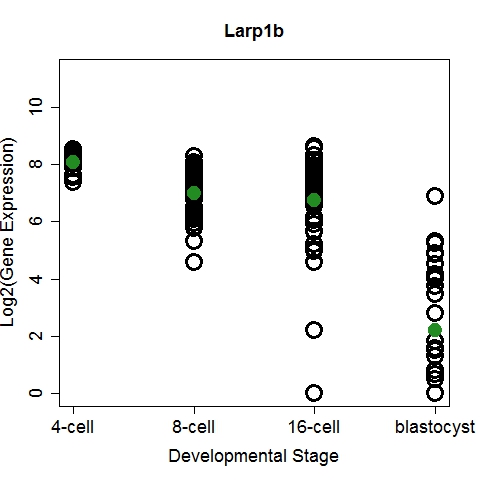

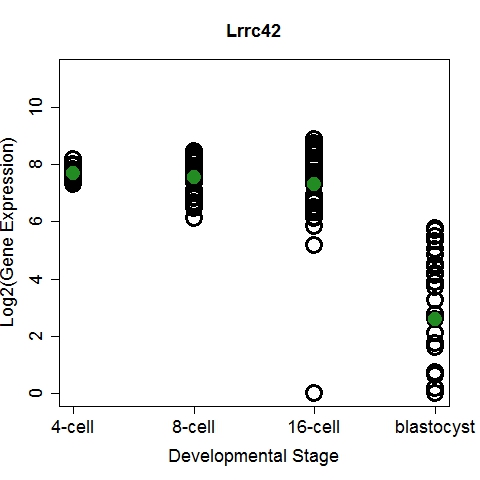

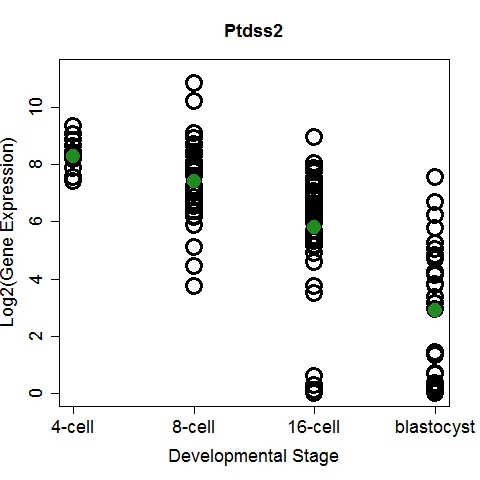

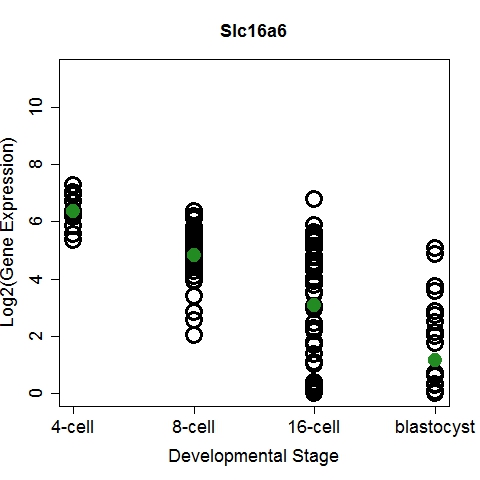

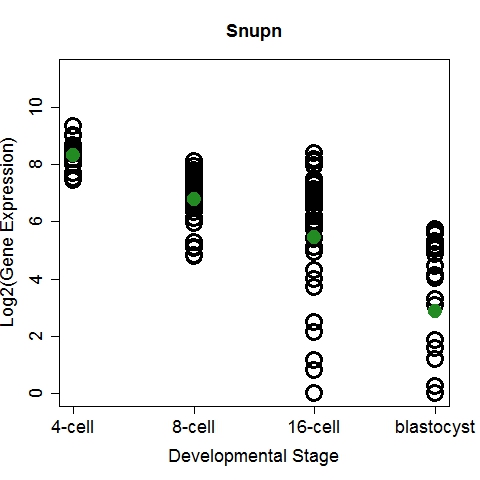

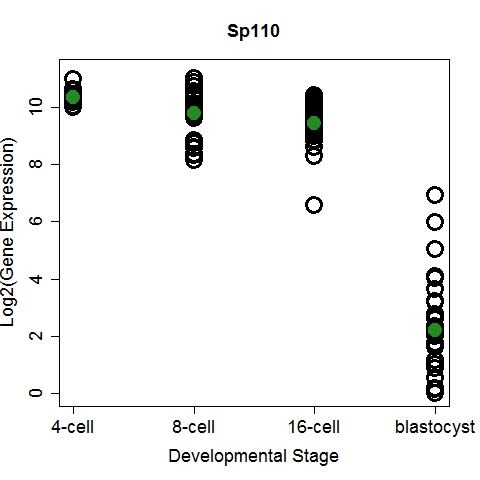

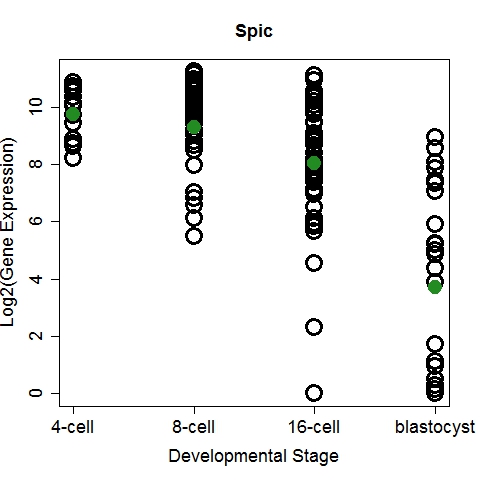


**Figure 14.** Twelve variability markers for the 8-cell stage from the Deng mouse embryo data set.


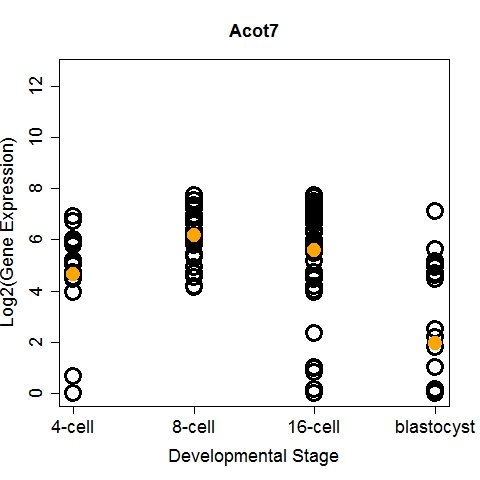

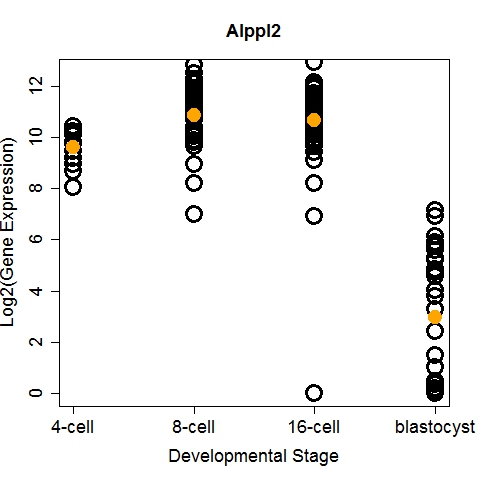

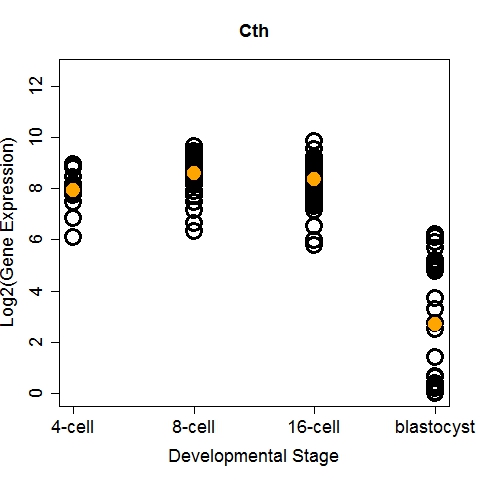

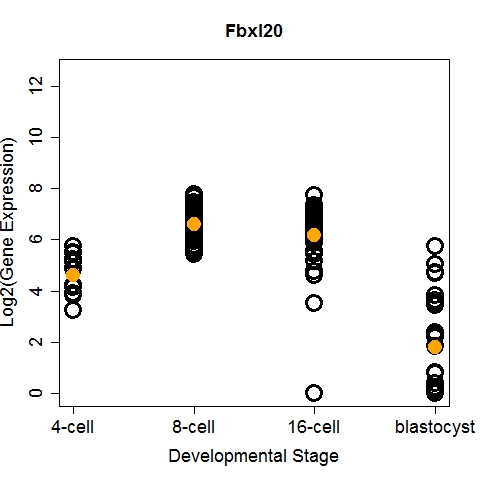

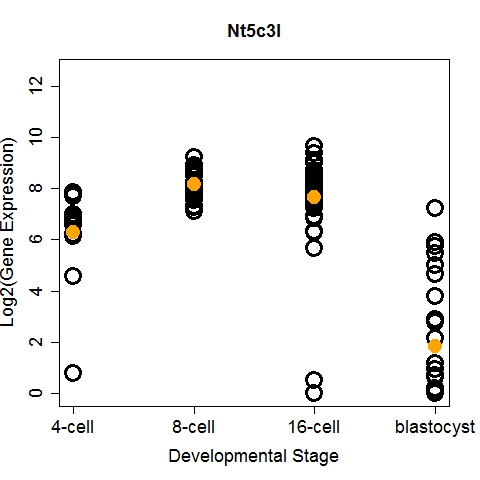

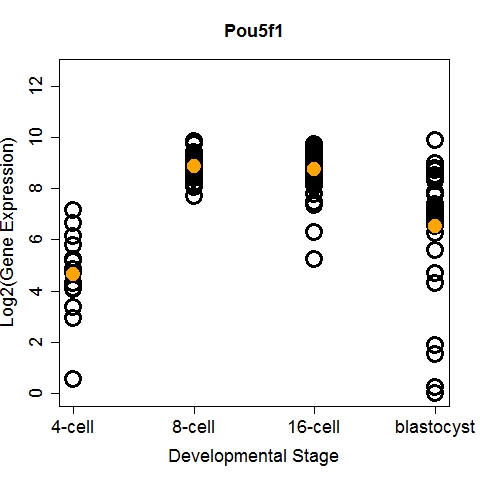

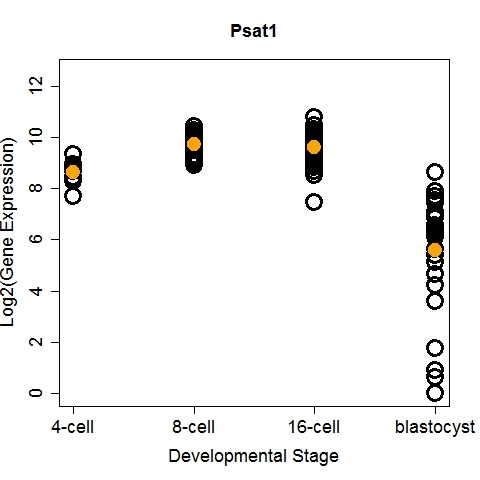

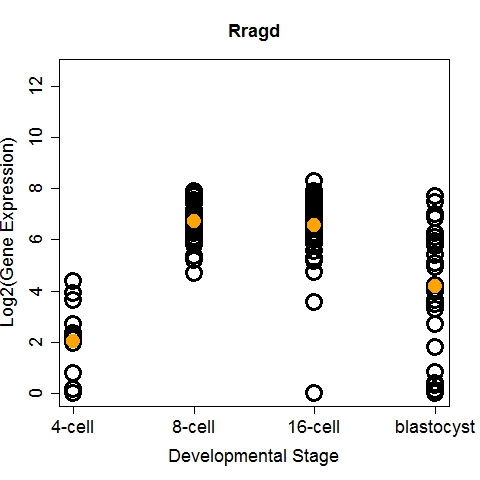

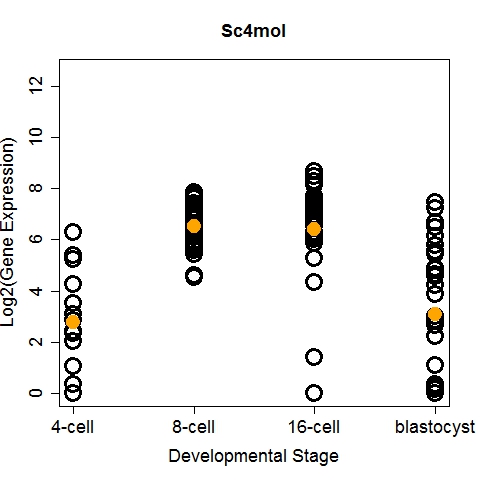

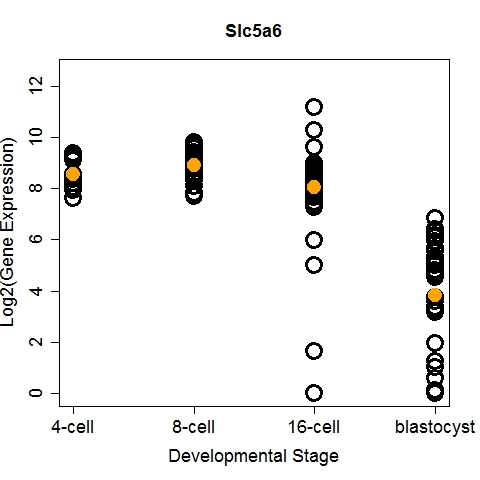

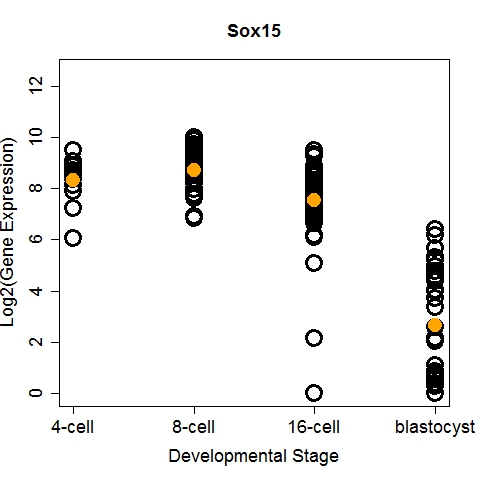

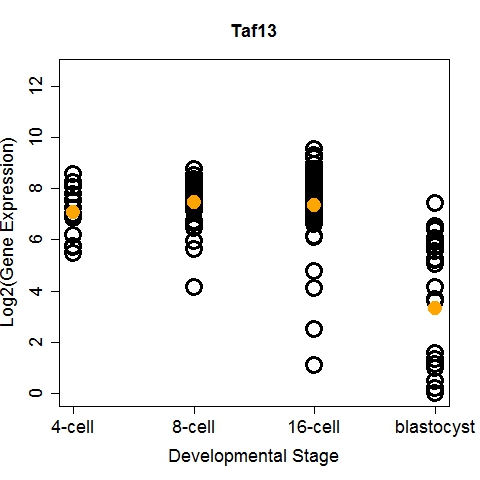


**Figure 15.** The two variability markers identified for the 16-cell stage from the Deng mouse embryo data set.


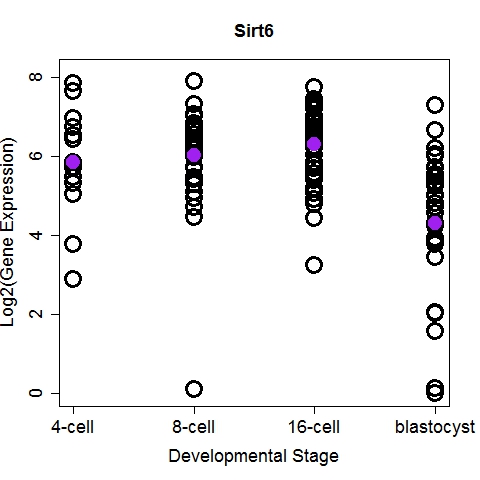

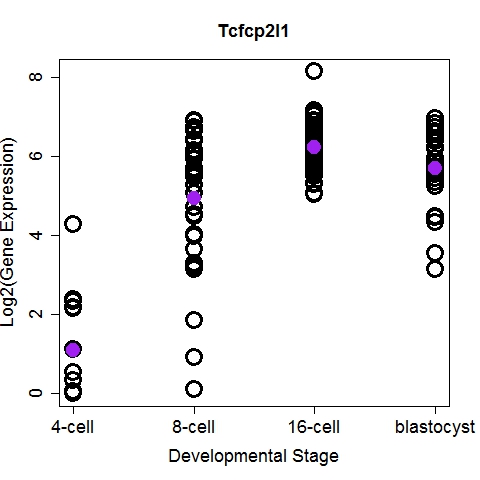


**Figure 16.** Twelve variability markers for the late blastocyst stage from the Deng mouse embryo data set.


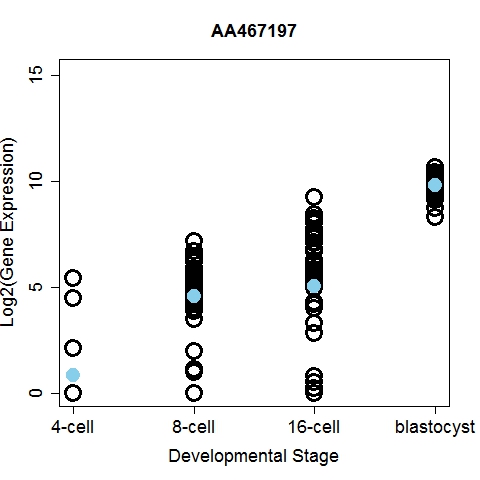

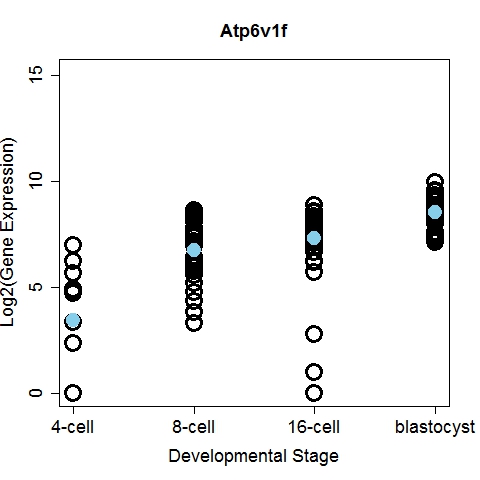

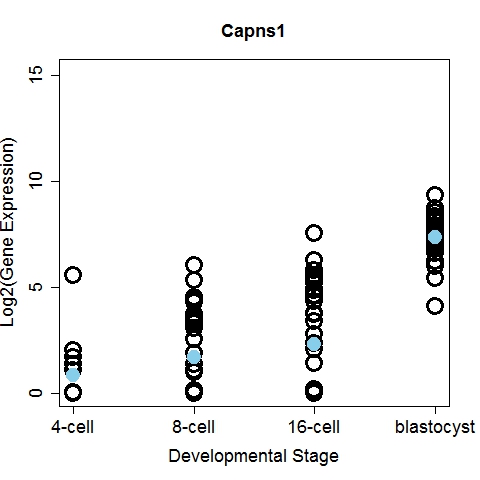

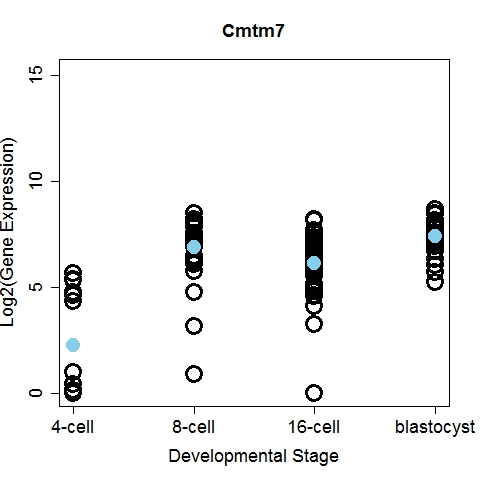

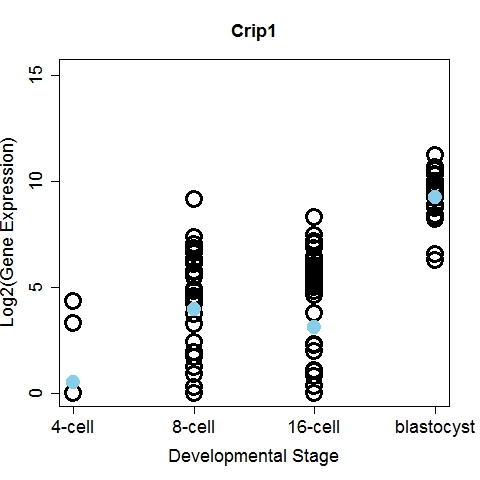

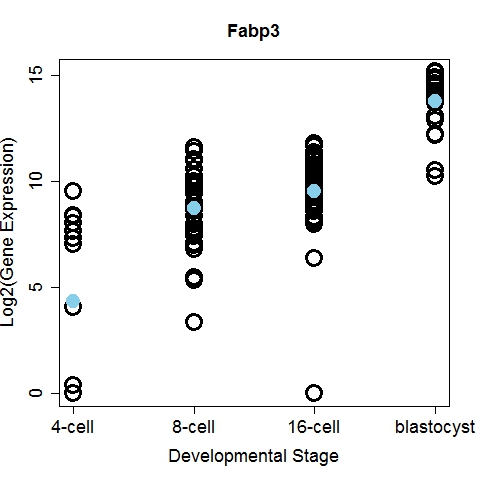

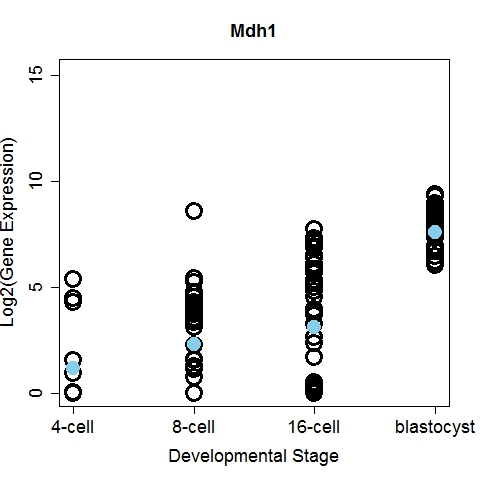

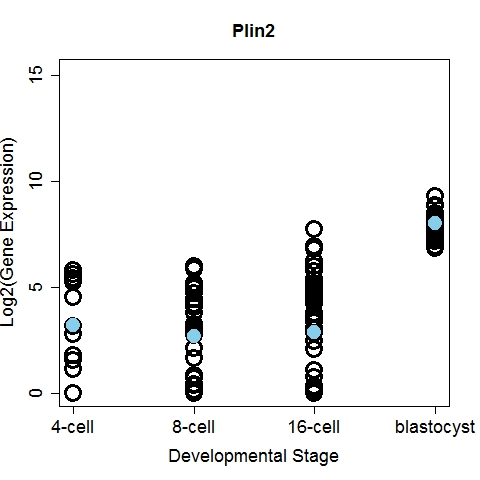

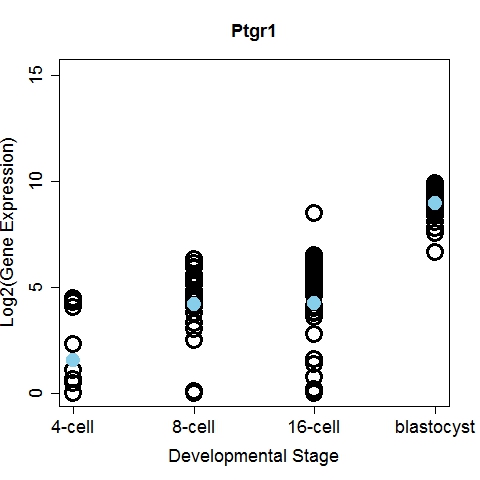

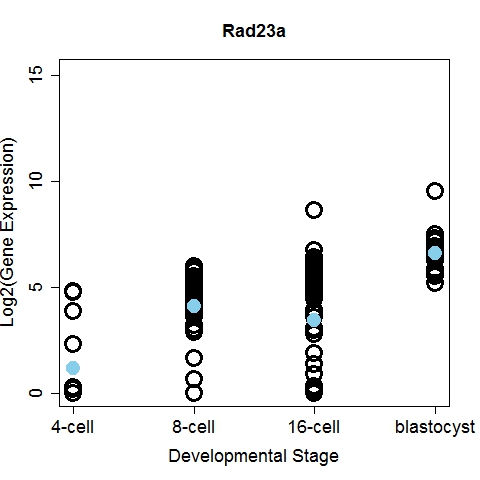

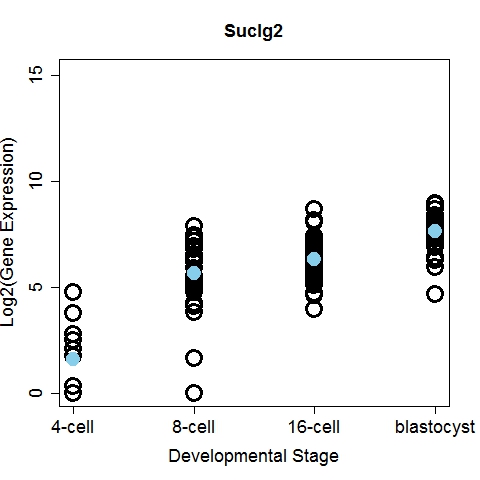

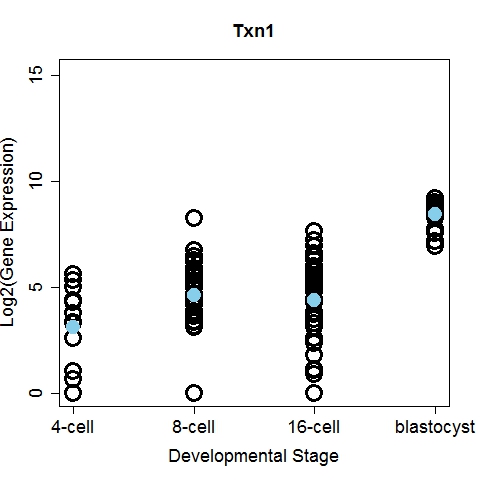


*The global distribution of intercellular gene expression variability also widens during mouse embryo development.*

**Figure 17.** Violin plots outline the distribution of inter-cellular expression variability for the Deng mouse embryo data set. We see that the distributions are widening as the embryos develop. The distribution of inter-embryo variability however remains relatively stable across all developmental stages.


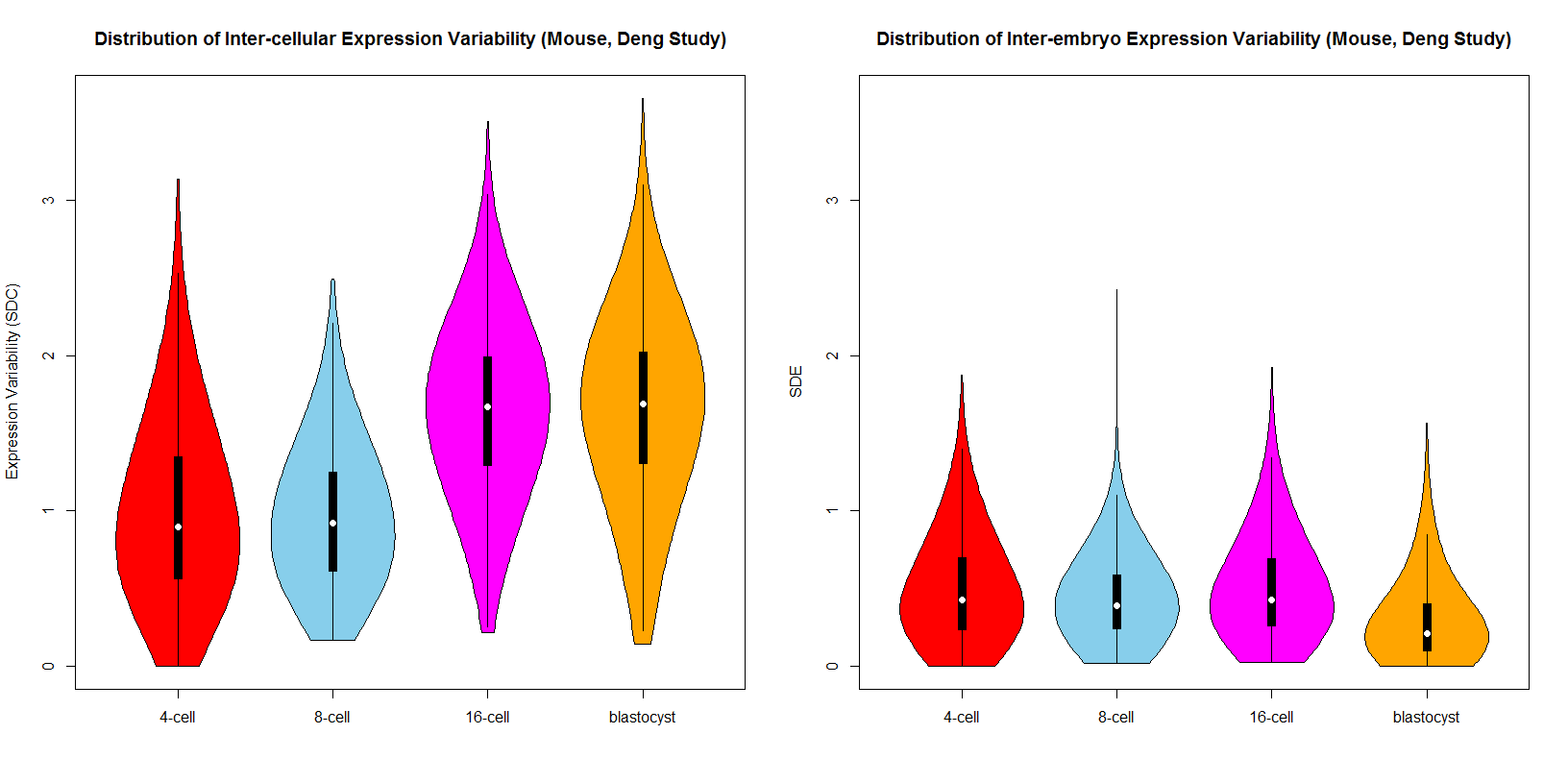


*Conclusion*

By applying our expression variability analysis, we were able to verify all key findings in the Deng mouse embryo data set that were found in the human embryo data set. Given that the Deng data set was profiled on a transcriptome-wide scale that was similar to the Yan data set, we also were able to find a statistically significant overlap in the stable genes from both human and mouse studies.
