# Supplementary material for: Variability of Gene Expression Identifies Transcriptional Regulators of Early Human Embryonic Development
Source: PLoS Genet. 2015 Aug 19;11(8):e1005428. doi: 10.1371/journal.pgen.1005428 (PMC4546122; doi:10.1371/journal.pgen.1005428)
Supplement: S1 Table — A subset of stable genes were also common to all stages. (DOCX) [file pgen.1005428.s016.docx]

**Table S1. The number of stable genes identified in each expression mode for a specific developmental stage.** A subset of stable genes were also common to all stages.

| ***Number of Stable Genes*** | **4-cell** | **8-cell** | **Morula** | **Blastocyst** | **Common to All Stages** |
| --- | --- | --- | --- | --- | --- |
| **Low Expression** | 291 | 277 | 284 | 318 | 229 |
| **Medium Expression** | 658 | 668 | 659 | 613 | 564 |
| **High Expression** | 6 | 10 | 12 | 24 | 4 |
| **Total per Stage** | 955 | 955 | 955 | 955 | 797 |
