# Supplementary material for: Variability of Gene Expression Identifies Transcriptional Regulators of Early Human Embryonic Development
Source: PLoS Genet. 2015 Aug 19;11(8):e1005428. doi: 10.1371/journal.pgen.1005428 (PMC4546122; doi:10.1371/journal.pgen.1005428)

**Table S2. Over-representation of IPA Function Annotation Terms for stable genes with low expression.** The IPA Function annotation terms that were enriched in the list of stable genes with low expression. Criteria for statistical significance was adjusted P-value < 0.05, number of molecules per term ≥ 10.


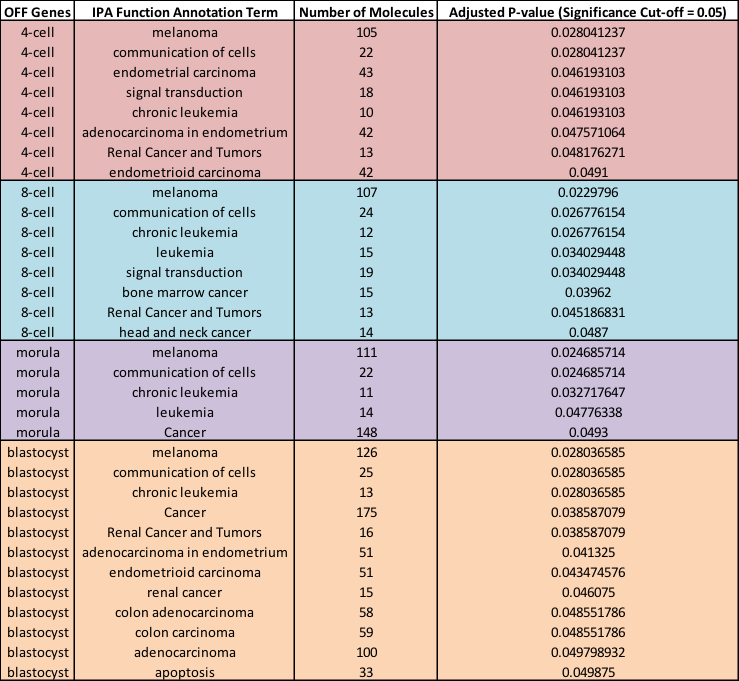

Supplement: S2 Table — The IPA Function annotation terms that were enriched in the list of stable genes with low expression. Criteria for statistical significance was adjusted P-value < 0.05, and number of molecules per term ≥ 10. (DOCX) [file pgen.1005428.s017.docx]
