# Supplementary material for: Variability of Gene Expression Identifies Transcriptional Regulators of Early Human Embryonic Development
Source: PLoS Genet. 2015 Aug 19;11(8):e1005428. doi: 10.1371/journal.pgen.1005428 (PMC4546122; doi:10.1371/journal.pgen.1005428)

**S3 Table. Over-representation of terms for stable genes with medium expression using IPA (A) Pathway Annotation terms and (B) Function Annotation terms**. **(A)** The IPA Pathway Annotation terms that were enriched in the list of stable genes with medium expression, where criteria for statistical significance was adjusted P-value < 0.01. **(B)** The IPA Function Annotation terms that were enriched in the list of stable genes with medium expression where criteria for statistical significance was adjusted P-value < 0.05, and number of molecules per term ≥ 10.

**(A)**


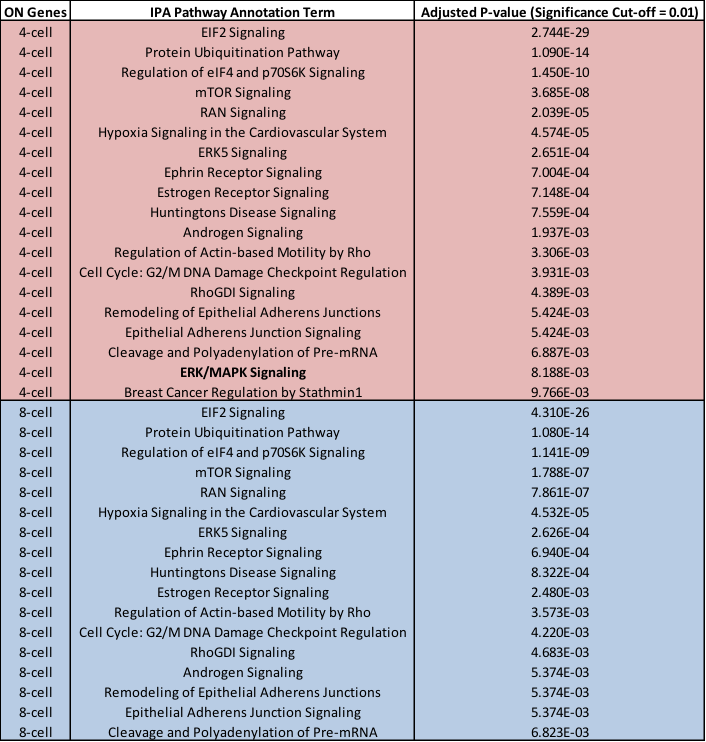


**(A, continued)**


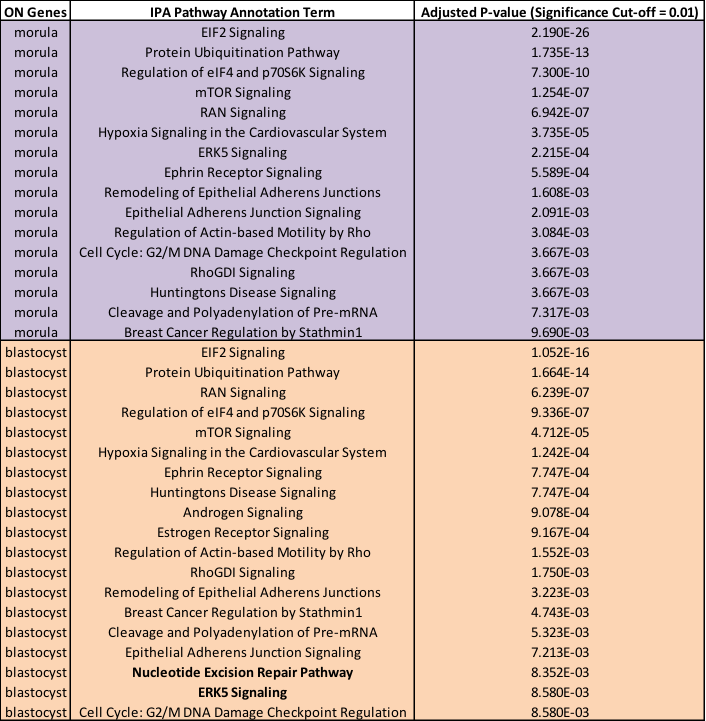


**(B)**


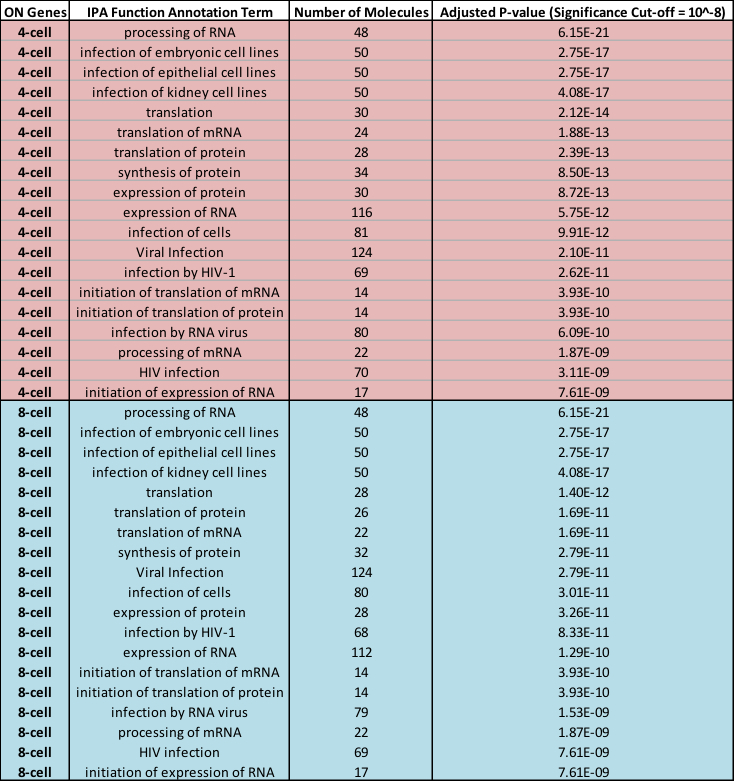


**(B, continued)**


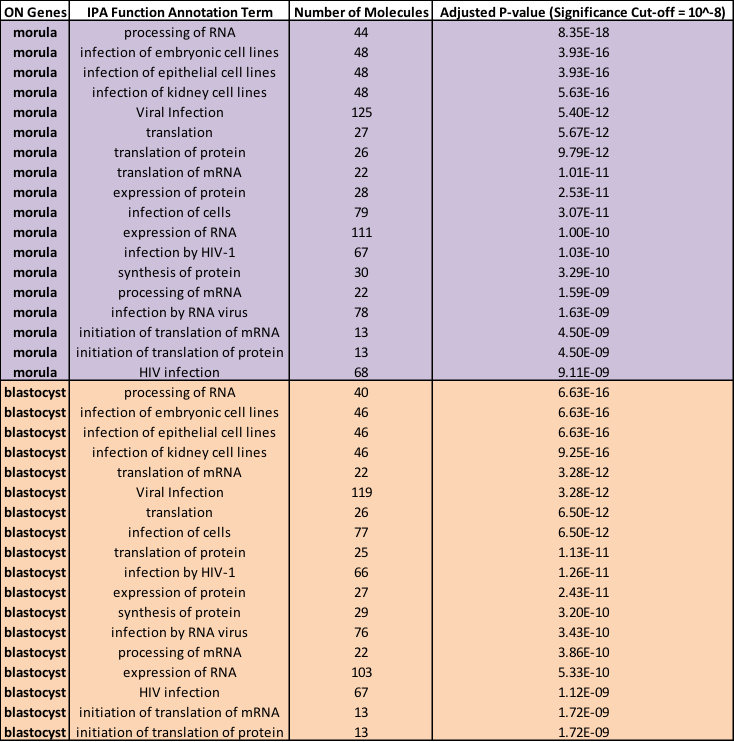

Supplement: S3 Table — (A) The IPA Pathway Annotation terms that were enriched in the list of stable genes with medium expression, where criteria for statistical significance was adjusted P-value < 0.01. (B) The IPA Function Annotation terms that were enriched in the list of stable genes with medium expression where criteria for statistical significance was adjusted P-value < 0.05, and number of molecules per term ≥ 10. (DOCX) [file pgen.1005428.s018.docx]
