# Supplementary material for: Variability of Gene Expression Identifies Transcriptional Regulators of Early Human Embryonic Development
Source: PLoS Genet. 2015 Aug 19;11(8):e1005428. doi: 10.1371/journal.pgen.1005428 (PMC4546122; doi:10.1371/journal.pgen.1005428)

**Table S4. Over-representation of IPA Pathway Annotation terms for stable genes with high expression**. The IPA Pathway Annotation terms that were enriched in the list of stable genes with high expression. Criteria for statistical significance was adjusted P-value < 0.01.


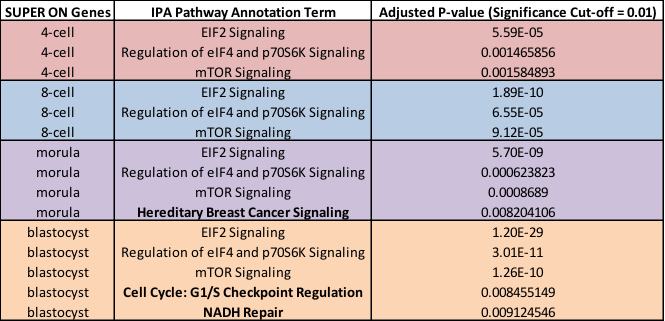

Supplement: S4 Table — The IPA Pathway Annotation terms that were enriched in the list of stable genes with high expression where criteria for statistical significance was adjusted P-value < 0.01. (DOCX) [file pgen.1005428.s019.docx]
