# Supplementary material for: Variability of Gene Expression Identifies Transcriptional Regulators of Early Human Embryonic Development
Source: PLoS Genet. 2015 Aug 19;11(8):e1005428. doi: 10.1371/journal.pgen.1005428 (PMC4546122; doi:10.1371/journal.pgen.1005428)

**S8 Table. Over-representation of IPA Pathway Annotation terms for genes at different levels of variability in the (A) 4-cell stage, (B) 8-cell stage, (C) morula stage, and (D) blastocyst stage**. The IPA Pathway Annotation terms that were enriched in genes for each level of variability identified (mixture 1 denotes the cluster of genes with the lowest level of variability, mixture 4 is the cluster with the highest level of variability). Criteria for statistical significance was adjusted P-value < 0.01.

**(A)**


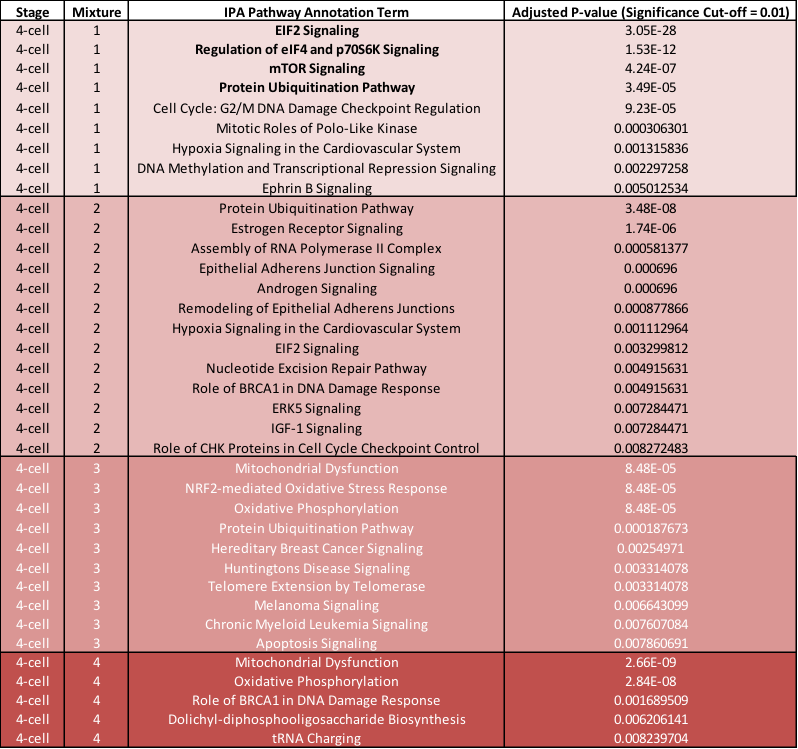


**(B)**


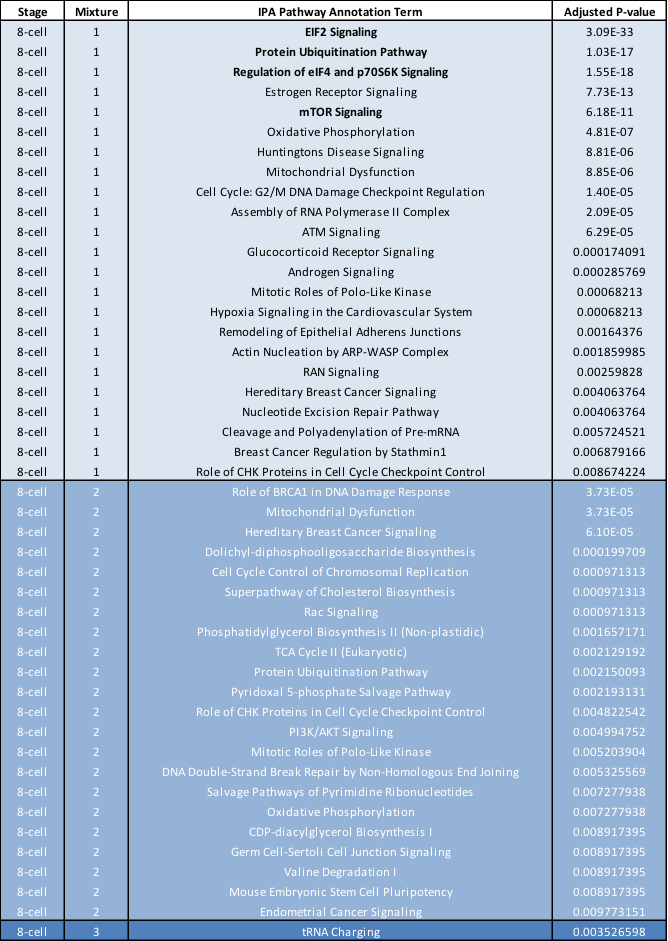


**(C)**


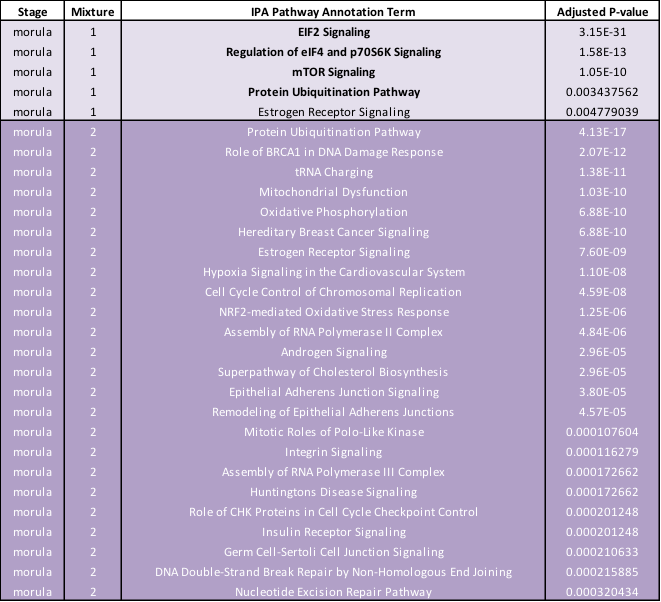


**(C, continued)**


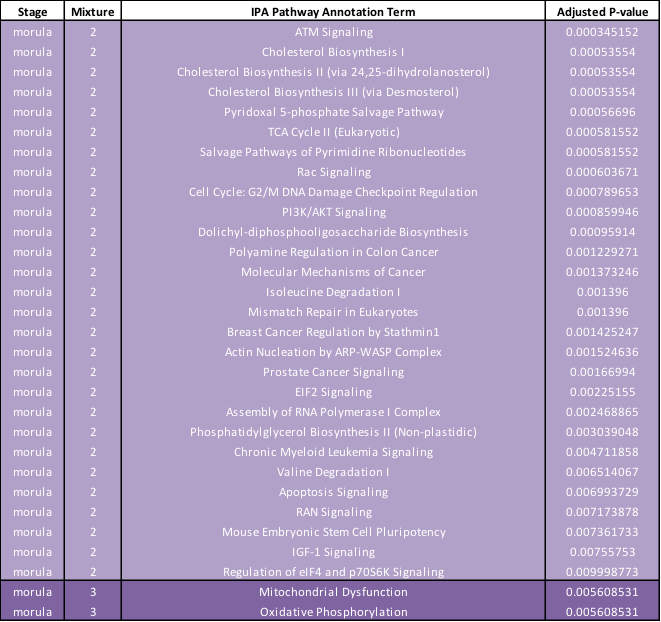


**(D)**


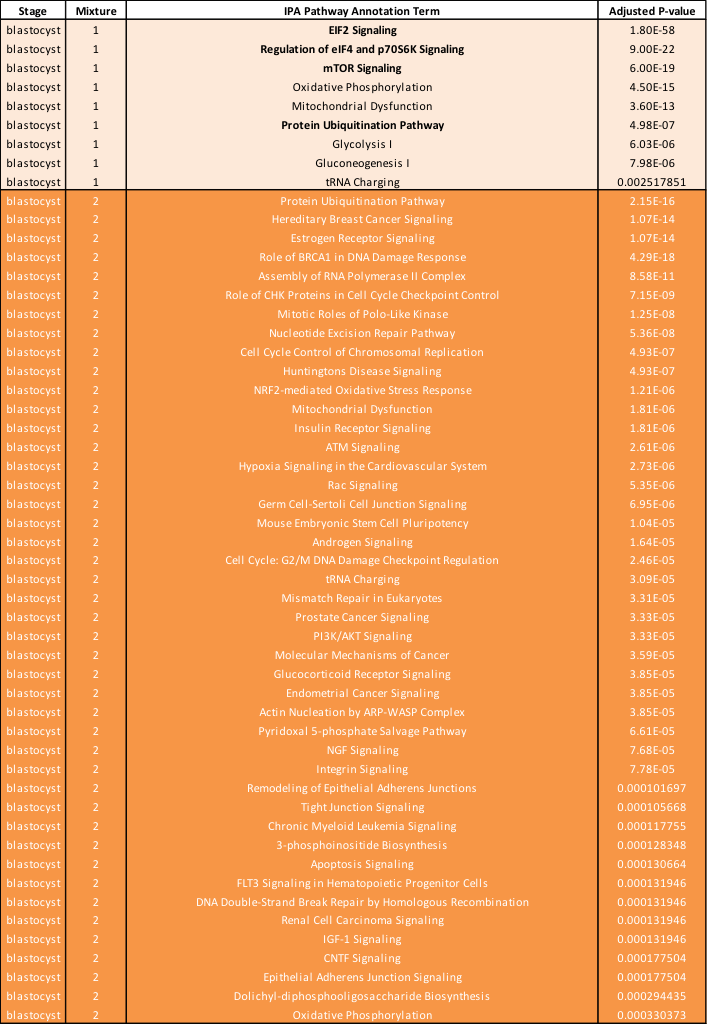


**(D)**


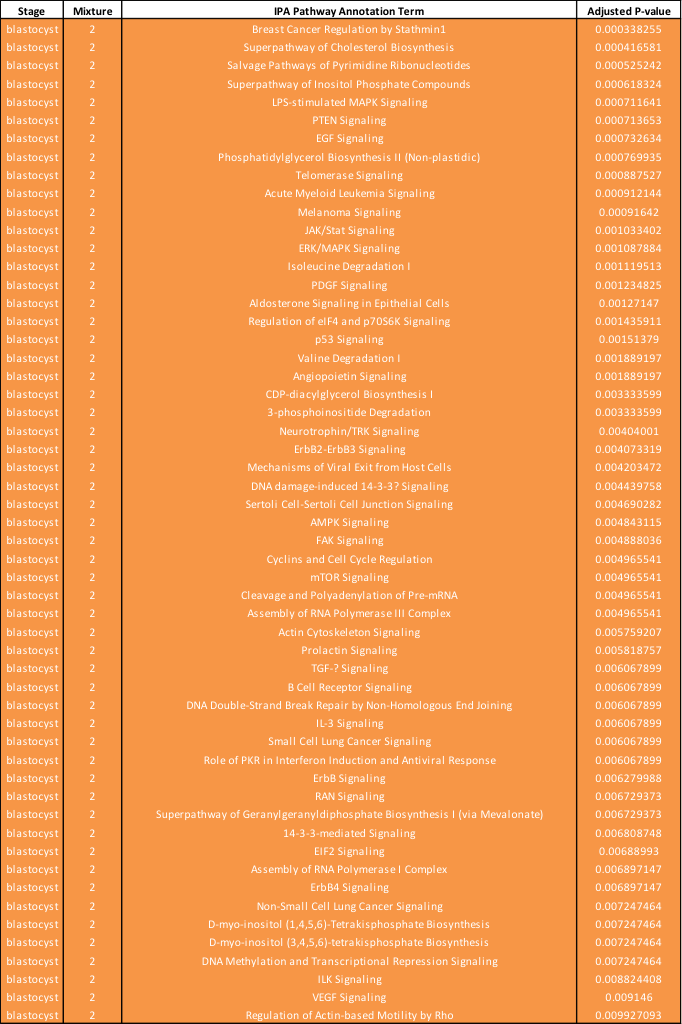

Supplement: S8 Table — The IPA Pathway Annotation terms that were enriched in genes for each level of variability identified (mixture 1 denotes the cluster of genes with the lowest level of variability, mixture 4 is the cluster with the highest level of variability). Criteria for statistical significance was adjusted P-value < 0.01. (DOCX) [file pgen.1005428.s023.docx]
