# Supplementary material for: Variability of Gene Expression Identifies Transcriptional Regulators of Early Human Embryonic Development
Source: PLoS Genet. 2015 Aug 19;11(8):e1005428. doi: 10.1371/journal.pgen.1005428 (PMC4546122; doi:10.1371/journal.pgen.1005428)
Supplement: S9 Table — (DOCX) [file pgen.1005428.s024.docx]

**Table S9.** The number of stable genes that are in common to all developmental stages and their overlap with the top 10% of ubiquitously expressed genes from de Jonge et al.

| Overlap between Common Stable Genes and Ubiquitously Expressed Genes | **Low Expression Stable Genes** | **Medium Expression Stable Genes** | **High Expression Stable Genes** |
| --- | --- | --- | --- |
| *Top 10% Ubiquitously Expressed Genes* | 0 | 198 (35%) | 4 (100%) |
| *Top 25% Ubiquitously Expressed Genes* | 4 (1.7%) | 310 (55%) | 4 (100%) |
| *Top 50% Ubiquitously Expressed Genes* | 24 (10%) | 372 (66%) | 4 (100%) |
| *Total Number of Genes Common to All Stages* | 229 | 564 | 4 |
