# Supplementary material for: Variability of Gene Expression Identifies Transcriptional Regulators of Early Human Embryonic Development
Source: PLoS Genet. 2015 Aug 19;11(8):e1005428. doi: 10.1371/journal.pgen.1005428 (PMC4546122; doi:10.1371/journal.pgen.1005428)
Supplement: S10 Table — (DOCX) [file pgen.1005428.s025.docx]

**Table S10.** The number of stable genes that are in common to all developmental stages and their overlap with the housekeeping genes identified by Eisenberg et al.

| Overlap between Common Stable Genes and Ubiquitously Expressed Genes | **Low Expression Stable Genes** | **Medium Expression Stable Genes** | **High Expression Stable Genes** |
| --- | --- | --- | --- |
| *List of Housekeeping Genes* | 5 (2.2%) | 342 (61%) | 1 (25%) |
| *Total Number of Genes Common to All Stages* | 229 | 564 | 4 |
