# Supplementary material for: Variability of Gene Expression Identifies Transcriptional Regulators of Early Human Embryonic Development
Source: PLoS Genet. 2015 Aug 19;11(8):e1005428. doi: 10.1371/journal.pgen.1005428 (PMC4546122; doi:10.1371/journal.pgen.1005428)
Supplement: S11 Table — (DOCX) [file pgen.1005428.s026.docx]

**Table S11. shRNA target sequences used in validation of *HDDC2*.**

| **shRNA identifier** | **Target sequence (21bp)** |
| --- | --- |
| Control (EGFP) | GCAAGCTGACCCTGAAGTTCAT |
| HDDC2 #1 | GCTAGACCAATGTGAAATGAT |
| HDDC2#2 | CCATTGTTGGTCTGTTGATTT |
