# Supplementary material for: Variability of Gene Expression Identifies Transcriptional Regulators of Early Human Embryonic Development
Source: PLoS Genet. 2015 Aug 19;11(8):e1005428. doi: 10.1371/journal.pgen.1005428 (PMC4546122; doi:10.1371/journal.pgen.1005428)
Supplement: S12 Table — (DOCX) [file pgen.1005428.s027.docx]

**Table S12. qPCR primers used in validation of *HDDC2*.**

| **qPCR target** | **Primer sequences (F+R)** |
| --- | --- |
| *GAPDH* | F - CTGGGCTACACTGAGCACC  R - AAGTGGTCGTTGAGGGCAATG |
| *HDDC2* | F - TGATATGGCAGAATGCATCG  R - GTCCTCTGGTAGGAGCTGGG |
| *NANOG* | F - TTTGTGGGCCTGAAGAAAACT  R - AGGGCTGTCCTGAATAAGCAG |
| *DNMT3B* | F - AGGGAAGACTCGATCCTCGTC  R - GTGTGTAGCTTAGCAGACTGG |
| *PAX6* | F – CAGCACCAGTGTCTACCAACCA  R - CAGATGTGAAGGAGGAAACCG |
