# Supplementary material for: Variability of Gene Expression Identifies Transcriptional Regulators of Early Human Embryonic Development
Source: PLoS Genet. 2015 Aug 19;11(8):e1005428. doi: 10.1371/journal.pgen.1005428 (PMC4546122; doi:10.1371/journal.pgen.1005428)
Supplement: S13 Table — (DOCX) [file pgen.1005428.s028.docx]

**Table S13. HDDC2 gRNA target sequences.**

| **gRNA identifier** | **Target sequence (20bp)** |
| --- | --- |
| HDDC2 #1 | CCACCGACCCCGGCTGGGCG |
| HDDC2#2 | GAAAGCGGAAGTGGGGGAGG |
| HDDC2#3 | CTCCGCCCAGCCGGGGTCGG |
